# Highly Active Oligoethylene Glycol Pleuromutilins via Systematic Linker Synthesis/One-Pot Attachment and a Microscale Solubility Method

**DOI:** 10.1021/acs.joc.4c02683

**Published:** 2024-12-18

**Authors:** Logan M. Breiner, Roman P. Slowinski, Andrew N. Lowell

**Affiliations:** † Department of Chemistry, Virginia Polytechnic Institute and State University (Virginia Tech), Blacksburg, Virginia 24061, United States; ‡ Center for Emerging, Zoonotic, and Arthropod-borne Pathogens, Virginia Polytechnic Institute and State University (Virginia Tech), Blacksburg, Virginia 24061, United States; § Department of Biochemistry, Virginia Polytechnic Institute and State University (Virginia Tech), Blacksburg, Virginia 24061, United States; ∥ Faculty of Health Sciences, Virginia Polytechnic Institute and State University (Virginia Tech), Blacksburg, Virginia 24061, United States

## Abstract

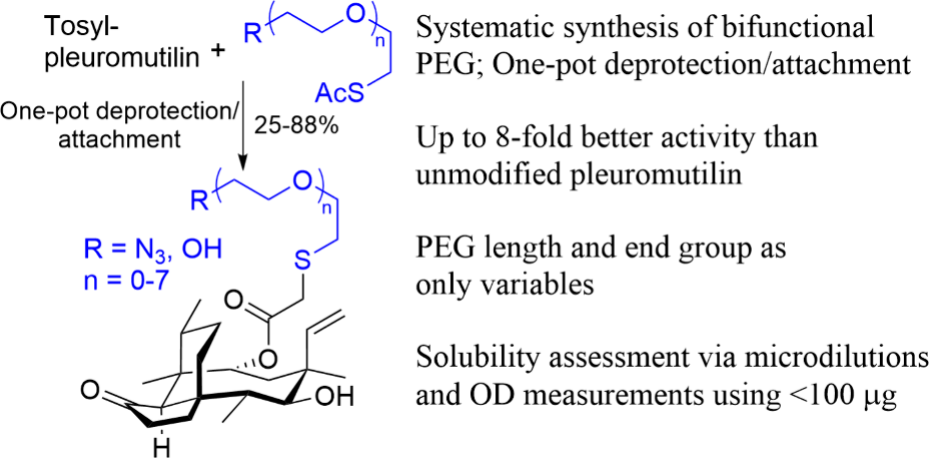

The semisynthetic derivatization of natural products is crucial for their continued development as antibiotics. While commercial pleuromutilin derivatives depend on amines for solubility, we demonstrate the high activity and solubility of oligoethylene glycol-substituted pleuromutilins achieved via a one-pot deprotection/attachment approach using thiolates protected as thioesters. The bifunctional linker synthesis is versatile and can be broadly applied to other chemistries. Antibacterial assays revealed this simple glycolate modification enhanced inhibition 4–8-fold relative to that of pleuromutilin. A new microscale solubility method is also introduced.

Antibiotics, particularly the vast majority derived from natural products, are outliers compared with typical small-molecule pharmaceuticals. In conventional medicinal chemistry, Lipinsky’s rule of five^1^ is used as a guidepost^2^ and further restrictions, such as Veber’s rule (<10 rotatable bonds and <140 Å^2^ total polar surface area), have been posited as additional indicators for drug-like molecules.^3^ While synthetically developed drug candidates seek to keep within these outlines, especially minimizing rotatable bonds to avoid conformational flexibility and thus lower potency, antibiotics routinely deviate from these rules.^4^ Because natural products are derived via fermentation, semisynthetic antibiotics based on them, such as azithromycin,^5^ tetracycline,^6^ and lefamulin,^7^ require careful planning and the use of highly orthogonal reactions. Ideally, semisynthetic derivatives can be achieved through simple and rapid diversification of available scaffolds and quickly result in new libraries of antimicrobial compounds.

Pleuromutilin (**1**, Figure 1), a diterpenoid fungal secondary metabolite,^8−10^ is a prime example of a successfully developed scaffold whose semisynthetic derivatives (**2** and **3**) have found use as clinical^11,12^ and veterinary^13^ antibiotics. Demonstrating potent activity against Gram-positive bacteria, fastidious Gram-negative bacteria, and mycobacteria,^14^ clinically relevant pleuromutilins contain a thioether substitution at C22 linking an amine to the natural product core. These modifications enable the formation of water-soluble salts, overcoming the poor water solubility of pleuromutilin itself (20 μg/mL).^15^ Despite their variance from the traditional parameters of pharmaceuticals, pleuromutilin derivatives have demonstrated a propensity for resistance avoidance^14^ and thus are prime targets for continued development.

**Figure 1:**
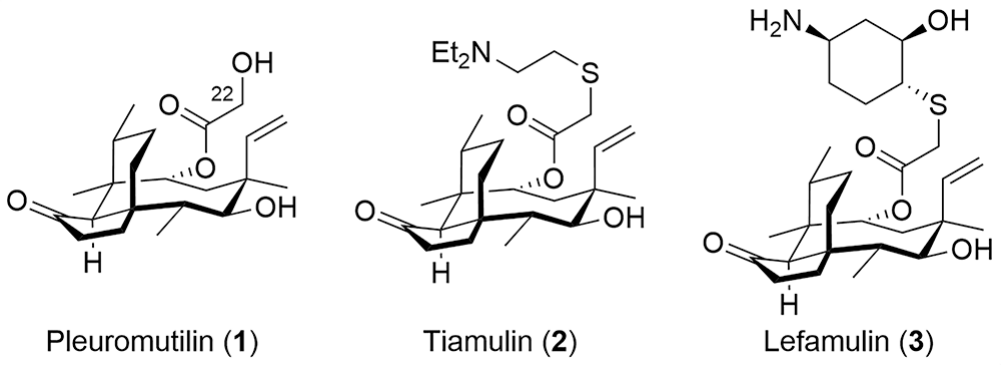
Pleuromutilin and its clinically relevant semisynthetic derivatives.

As part of ongoing efforts to synthesize conjugate antibiotics, we sought to functionalize pleuromutilin with varying length oligoethylene glycol (OEG) chains. Oligoethylene glycol chains are attractive linker substrates because they are inexpensive, readily available, biocompatible, and water-soluble.^16^ By using linkers starting with a thiol and terminating with an azide, these derivatives would mimic the C22 thiol connection of all clinically approved pleuromutilin derivatives, while also promoting orthogonal reaction conditions. The azide would facilitate copper-catalyzed azide alkyne cycloaddition reactions^17,18^ with other highly functionalized natural products. The excellent nucleophilicity of thiolate species would enable facile attachment to activated pleuromutilin.^19,20^ However, due to the ease with which thiols can be oxidized, this group would need to be protected prior to use.

While common,^21−26^ the use of linkers to attach two or more materials tends to be system specific and is rarely discussed systematically.^27−30^ We thus systemized the synthesis of azido/thiol-terminated OEGs and connected them to pleuromutilin using a one-pot deprotection and attachment strategy. To our surprise, these compounds were highly active in and of themselves, leading us to test them and hydroxy-terminated OEG derivatives against a series of pathogens.

OEGs from di- to pentaethylene glycol (**4**–**7**, respectively, Scheme 1) were first monofunctionalized as the tosylate (**8**–**11**) by using an excess^31^ of the OEGs. Treatment of **8**–**11** with sodium azide^32^ gave azido-hydroxy OEGs **12**–**15**. Initial displacement with azide was chosen over the thioacetate due to their relative stabilities. The remaining hydroxy moiety of **12**–**15** was again activated via tosylation^31^ to give **16**–**19**. Finally, displacement of the tosylate with thioacetate^33^ was performed, yielding thioesters **20**–**23**, respectively, masked thiols stable for storage and use as nucleophiles after *in situ* deprotection.

**Scheme 1:**
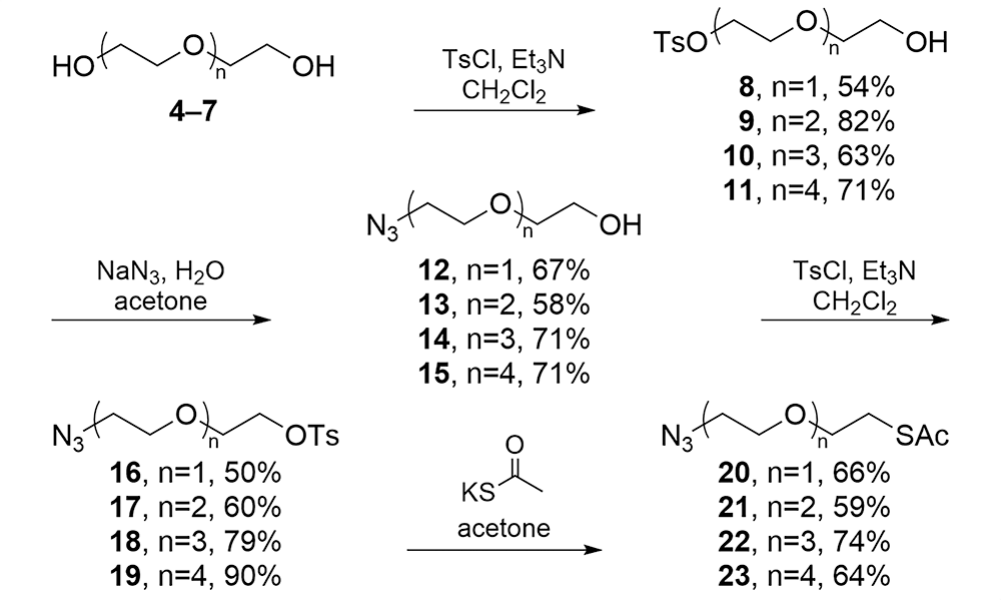
Synthesis of Azido-Terminated Bifunctional OEGs

While this process was straightforward, practical challenges arose due to the linear and highly flexible nature of the linkers. Purification via recrystallization was not possible, necessitating chromatographic methods, in which the OEG chains behaved poorly. Due to the large number of repeating ether functional groups, the behavior of the products was very similar to that of the starting materials, especially once the more polar hydroxy end groups were masked or transformed. Some OEGs also gave non-Gaussian distributions during chromatography, consistently eluting as multiple discrete peaks. Chromatographic separation only after the initial activation (to eliminate remaining OEGs and ditosylate byproducts) and after formation of the final thioacetate minimized waste while achieving purity. Complete consumption of intermediates in the intervening steps was ensured by using an excess of the requisite nucleophile or electrophile and the removal of excess reagents via extraction. Reactions were conducted under an inert atmosphere, and mixtures processed rapidly because of the propensity of OEGs to form organic peroxides;^34^ products and intermediates were stored cold and protected from light and oxygen.

To attach the functionalized OEGs to tosylpleuromutilin^18^ (**24**, Scheme 2), we created a one-pot procedure by adapting protocols for deprotection of a thioester^33^ and nucleophilic displacement using a thiolate.^19^ The one-pot procedure using sodium hydroxide proved to be convenient and effective, converting **20**–**23** into **29**–**32**, respectively (*n* = 1–4), in good yields (49–88%). The *n* = 0 compound (**28**) was synthesized from **24** via a multistep route to avoid the generation of low-molecular weight organic azides that would violate the azide heavy atom rule and potentially be explosively unstable to friction, shock, and heat.^35^ First, the tosylate of **24** was displaced by using a thiolate generated from β-mercaptoethanol and potassium *tert*-butoxide to give **25**. Hydroxy-terminated **25** was then activated as tosylate **26**, which was subsequently displaced with azide to furnish **28**, completing the series of azido-terminated C22-OEG pleuromutilins for which *n* = 0–4. Contrasting with the free linkers, **28**–**32** behaved well during chromatography, likely because the pleuromutilin core added nonpolar functionality that enabled better separation. The direct attachment of the azido group to C22 (**27**, not shown) was synthesized previously by direct azide displacement of **24**.^18^

**Scheme 2:**
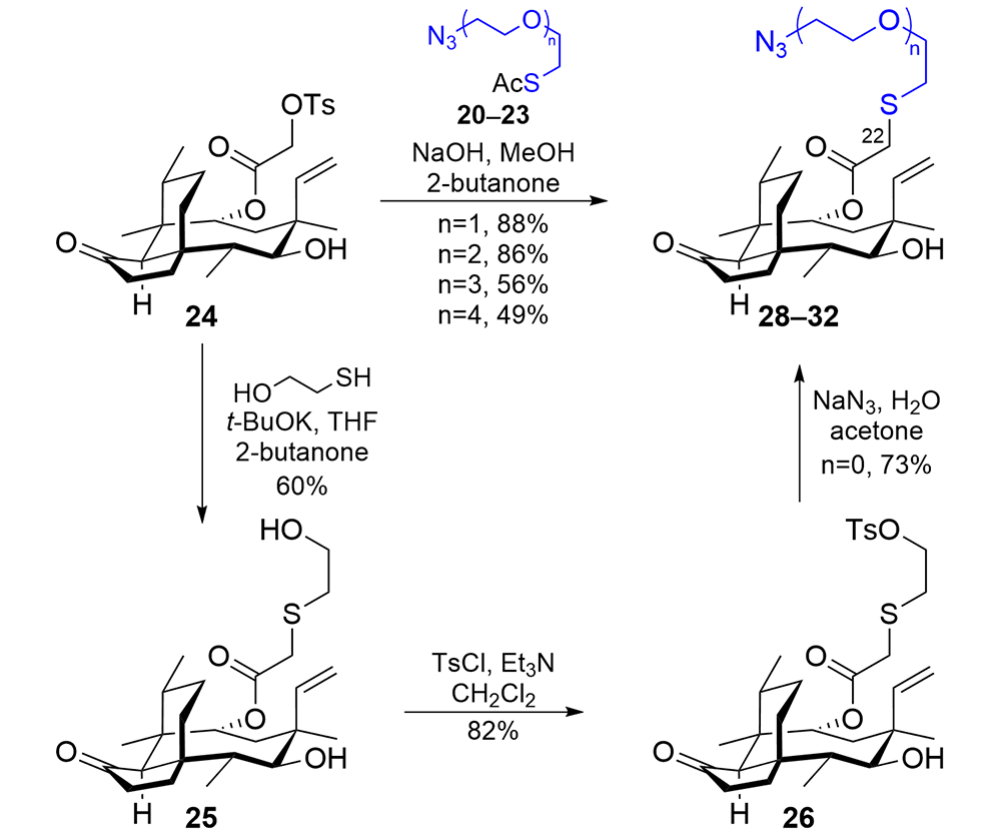
Incorporation of Azido-Terminated OEGs onto the Pleuromutilin Scaffold

To determine if the antimicrobial activity of PEG-derived pleuromutilins was due to the functionalized azido terminus or an inherent property of the OEG, we synthesized hydroxy-terminated OEG chains and attached them to pleuromutilin (Scheme 3). Activation using tosyl chloride in the presence of excess OEGs proceeded as described above for **8**–**11** and was expanded to include OEG6 (**35**) and OEG8 (**36**) to better assess activity as a function of an increase in OEG length. Displacement using potassium thioacetate furnished hydroxy- and thioester-terminated OEGs **37**–**42**. Using a similar one-pot protocol, **37**–**42** were deprotected and appended onto **24**, giving **43**–**48**, respectively. This work shows a straightforward way to make highly active azido- and hydroxy-terminated PEGs of various lengths with masked thiolate nucleophiles broadly suitable for use in linker chemistry and demonstrates their utility by directly attaching them via the S_N_2 reaction to activated natural products.

**Scheme 3:**
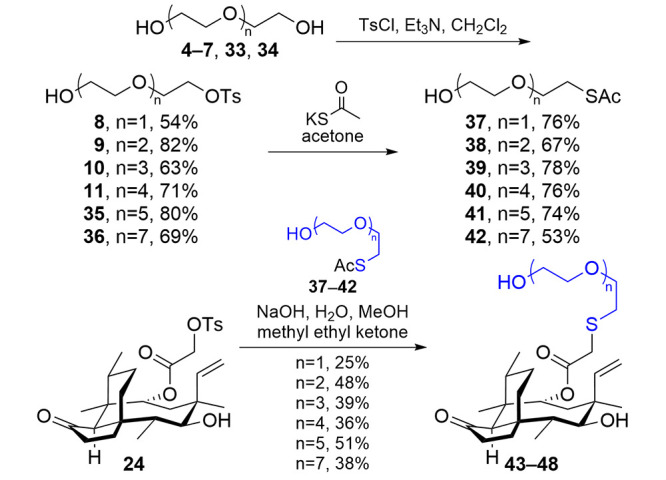
Synthesis of Hydroxy-Terminated Monofunctional OEGs and Their Incorporation onto the Pleuromutilin Scaffold

Both series of OEG–pleuromutilin derivatives showed enhanced activity against Gram-positive bacteria relative to that of pleuromutilin (**1**, Table 1), with the activity of the azido-terminated compounds (**28**–**32**) generally being greater than that of the hydroxy-terminated compounds (**25** and **43**–**48**). While the azido group directly attached to the C22 position (**27**)^18^ did not significantly improve activity, shorter azido OEG chains (**28** and **29**) showed the greatest improvement in activity (8-fold improvement), with longer chain lengths (**29**–**32**) showing a smaller improvement in activity (4-fold improvement). While activity was good against *Staphylococcus aureus* strains and vancomycin resistant enterococcus (VRE), all compounds were inactive against *Enterococcus faecalis* (>100 μM). This general trend held true for the hydroxyl OEG–pleuromutilins (**25** and **43**–**48**) with the addition of the longer linkers, especially OEG8 **48**, demonstrating the size limits of OEG functionalization at this site. The exception is against VRE, for which all pleuromutilin–OEG derivatives of both the N_3_ and OH varieties showed equal activity (4-fold potency compared to **1**) except for **28** (8-fold potency), indicating that the size cap had not been reached.

**Table 1:** Antibacterial Activity of the OEG–Pleuromutilin Derivatives

compound^a^	MRSA 43300	*S. aureus* 6538P	VRE	*E. coli* MC1061	*E. coli* ΔTolC
**27**	1.56	1.56	12.5	12.5	1.56
**28**	0.391	0.391	1.56	12.5	0.195
**29**	0.391	0.781	3.12	25	0.391
**30**	0.781	0.781	3.12	25	0.391
**31**	0.781	0.781	3.12	50	0.391
**32**	0.781	0.781	3.12	25	0.391
**25**	0.781	0.781	3.12	3.12	0.391
**43**	0.781	0.781	3.12	6.25	0.391
**44**	1.56	0.781	3.12	12.5	0.391
**45**	1.56	0.781	3.12	12.5	0.781
**46**	1.56	0.781	3.12	12.5	0.781
**47**	1.56	0.781	3.12	25	0.781
**48**	3.12	1.56	3.12	25	1.56
**1**	3.12	3.12	12.5	3.12	3.12

a Minimum inhibitory concentrations in micromolar. *E. faecalis*, *A. baumannii*, and *K. pneumoniae* were also tested, but the activity was >100 μM.

Pleuromutilin–OEG derivatives were inactive against Gram-negative pathogens *Acinetobacter baumannii* and *Klebsiella pneumoniae* and were less active than **1** in *Escherichia coli* MC1061, with the same overall trend of activity decreasing with an increase in chain length. However, the hydroxy series (**25** and **43**–**48**) performed better than the azide series (**28**–**32**) at equivalent lengths, a reversal of the trend seen in Gram-positive *S. aureus*, which suggests the penetration properties of hydroxy-capped OEG are more favorable than those of the azido-capped variants.^36^ In *E. coli* with a TolC knockout (ΔTolC), all azide-terminated pleuromutilin–OEG derivatives were highly potent (8-fold increase over **1**) as were the shorter members of the hydroxy-terminated series. On the basis of these results, pleuromutilin–OEG derivatives appear to be more easily recognized as substrates for the AcrAB–TolC efflux pump, likely because of their resemblance to non-ionic surfactants.^37^

Efflux notwithstanding, inclusion of long, highly flexible OEG chains improved rather than worsened activity. Generally, compound optimization in medicinal chemistry seeks to reduce the number of rotatable bonds, preventing the molecule from sampling multiple conformational arrangements in the binding site, thus reducing binding efficiency, and better enabling cell membrane transit.^3^ The reversal of this trend for these compounds may be because they have enhanced uptake due to their amphiphilic nature with the hydrophobic pleuromutilin core and the hydrophilic OEG chains directly enhancing penetration through or transport across the cell membrane.^38^ Another explanation may be that the binding of pleuromutilin–OEG derivatives to their target sites is more entropically favorable compared to that of **1**. Highly hydrophobic molecules, such as **1**, must be contained within an ordered hydrogen bonding network, an entropically unfavorable state that is partially reduced once the molecule binds its target site, effectively shielding a portion of itself from the organized solvent.^39,40^ The OEG chains may further disrupt the hydrogen-bonding network, increasing the level of shielding and making the binding process even more entropically favorable. Another alternative, especially with regard to shorter chains having a higher efficacy, is that the OEG chains may have their own discrete binding interactions within the ribosome.

During the antimicrobial assays, an increase in optical density was observed at higher concentrations of the pleuromutilin–OEG derivatives, specifically, a visible precipitate that was morphologically distinct from the growing bacteria. Coupled with precipitation observed over a concentration range of 50–100 μM in the pleuromutilin control (a value in accord with its reported solubility),^15^ this serendipitous finding suggested that we could use optical density readings to gauge solubility. Overall, we anticipated that introducing hydrophilic OEG chains onto a hydrophobic molecule, such as pleuromutilin, would enhance the water solubility of the resulting derivatives. A PEG functionalization strategy has been used previously to create water-soluble prodrugs,^41^ including an example with a pleuromutilin derivative,^42^ and PEG conjugation can confer additional benefits,^43^ such as increasing the extent of drug circulation in the blood and preventing its excretion, and preventing immune recognition of biologics. Our approach differs in using low-molecular weight OEGs, which are incorporated into the drug itself when it binds its target, and our focus on antibiotics.

Azide-terminated compounds **28**–**32** were less soluble at shorter chain lengths, with solubility increasing as the chain length increased, showing that an increase in the number of hydrogen bond-accepting ether groups tracks with increased solubility. The hydroxy-terminated series (**25** and **43**–**48**) all showed solubilities of >200 μM, a minimum of a 4-fold increase relative to that of pleuromutilin (Table 2). This result indicates that the presence of a more flexible terminal hydrogen-bond-donating group is critical for increased solubility. As a common problem with pleuromutilin derivatives is their low solubility, which leads to poor bioavailability,^44^ this facile method of functionalization may provide a way to create potent pleuromutilin-derived antibiotics with better pharmacokinetics.^45^ Furthermore, these results indicate that customary amine functionality, such as in **2** and **3**, may not be necessary for bioavailability.

**Table 2:**
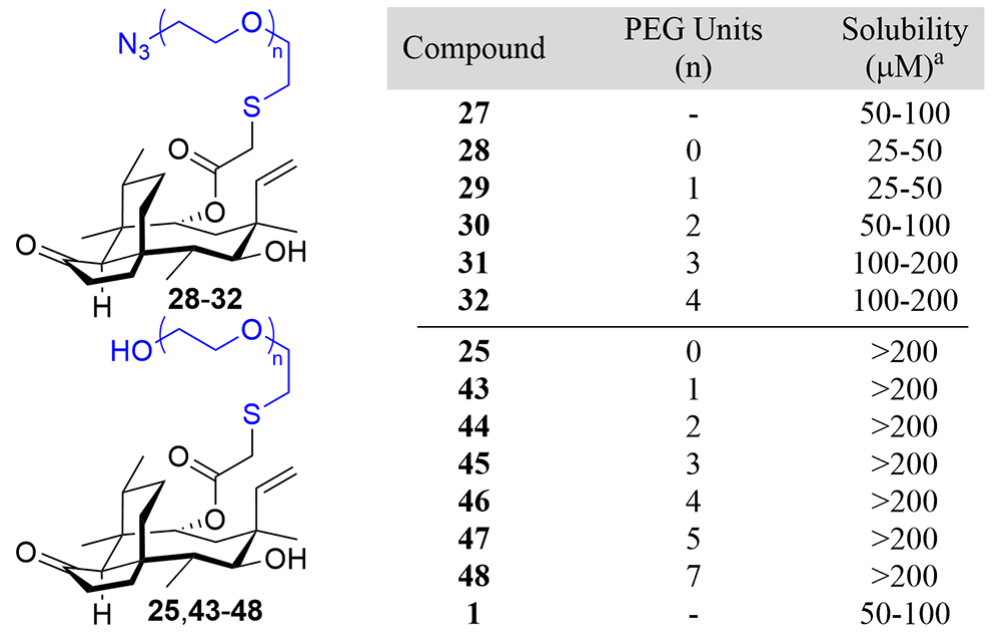
Solubility Ranges of Pleuromutilin–OEG Derivatives

a In 2.5% (v/v) DMSO/aqueous media. Concentrated solutions of the derivatives were serially diluted in 100% DMSO and then mixed with aqueous media to achieve the desired concentrations. After incubation (37 °C for 1 day), the optical density was assessed using a plate reader. Listed ranges are between the soluble and insoluble wells.

While there are a variety of methods for determining solubility, they are often complicated and require substantial quantities of material.^46^ By utilizing an ultraviolet–visible (UV–vis) plate reader, the aqueous solubility of antibiotics was determined in a straightforward manner simultaneously with the minimum inhibitory concentrations. This method differs from other assay plate methods^47,48^ in that only small amounts (∼50 μg) of the valuable compound are needed, it is facile to conduct because it requires no evaporation stage, and it can be applied alongside other assays.

Small quantities are highly desirable when working with a precious material. For example, LogP measurements are so material and labor intensive^49^ that calculated values are commonly used. The efficiency of this assay could be further increased by using 384- or 1536-well plates, requiring even less material. It could also be used for non-aqueous solvent systems, provided volatility is controlled. This method could also be extended to non-drug-like molecules where large quantities are undesirable, such as for highly toxic or explosive compounds.

The drawbacks of this assay are that a cosolvent was required for the initial administration of the dissolved derivatives to the test wells. In this case, DMSO was used, resulting in a final DMSO concentration of 2.5%. The test concentrations are dictated by the saturation concentration of the material in the cosolvent, although reversal of the solvents could make this method applicable to non-organic soluble materials. Another drawback is that the solubility measurements are obtained as ranges and not as specific values; however, once an initial range is determined, additional assays with more precise dilutions could be used to give more accurate measurements. Lastly, supersaturation is a potential concern; however, the precipitation of compounds resistant to crystallization with another solvent is an established method, and the design of this assay mirrors that technique.

In summary, we have demonstrated that azido- and hydroxy-terminated OEG linkers bearing a masked thiol can be efficiently prepared and straightforwardly attached to the natural product pleuromutilin using a one-pot deprotection/attachment procedure. The resulting OEG–pleuromutilin derivatives have enhanced activity relative to the natural product as well as increased water solubility indicating that amine-containing side chains may not be necessary for high activity with this class of natural products. Solubility was determined using serial dilutions in a microplate and visualized with a commonly available UV–vis plate reader, a new method that required only small amounts of the compound (<100 μg). These findings and ongoing collaborations^18^ enable the continued semisynthetic development of pleuromutilin for antibiotic lead discovery.

## Data Availability

The data underlying this study are available in the published article and its Supporting Information.

## Supporting Information Available

Synthetic procedures, assay details, and ^1^H and ^13^C nuclear magnetic resonance spectra (PDF)
