# Supplementary material for: Highly Active Oligoethylene Glycol Pleuromutilins via Systematic Linker Synthesis/One-Pot Attachment and a Microscale Solubility Method
Source: J Org Chem. 2024 Dec 18;90(1):919–24. doi: 10.1021/acs.joc.4c02683 (PMC11731290; doi:10.1021/acs.joc.4c02683)
Supplement: Supplementary file 1 — jo4c02683_si_001.pdf [file jo4c02683_si_001.pdf]

Highly active oligoethylene glycol pleuromutilins via systematic linker synthesis/one-pot attachment and a microscale solubility method

Logan M. Breiner[1,2], Roman P. Slowinski[1,3], and Andrew N. Lowell[1,2,4] *

Supplementary Information

1 Department of Chemistry, Virginia Polytechnic Institute and State University (Virginia Tech), Blacksburg, Virginia 24061, United States

2 Center for Emerging, Zoonotic, and Arthropod-borne Pathogens, Virginia Polytechnic Institute and State University (Virginia Tech), Blacksburg, VA 24061, United States

3 Department of Biochemistry, Virginia Polytechnic Institute and State University (Virginia Tech), Blacksburg, Virginia 24061, United States

4 Faculty of Health Sciences, Virginia Polytechnic Institute and State University (Virginia Tech), Blacksburg, VA 24061, United States

*Email: alowell@vt.edu

Table of Contents

Title Page S1

Table of Contents S1

General Experimental S2-S3

Synthetic Procedures and Characterization for Compounds **8** – **26**, **28** – **32**, **35** – **48** S3-S12

1H and 13C{1H} NMR Spectra S13-S39

References S40

Synthesis

Unless otherwise noted, chemical reagents and solvents were purchased from EMD Millipore, Oakwood Chemical, Sigma-Aldrich, and Thermo Fisher Scientific. Unless otherwise specified, all reactions were carried out under an atmosphere of dry nitrogen in dried glassware. Commercially available starting materials and reagents were used as received or purified prior to use if necessary. Triethylamine was distilled from calcium hydride, and pyridine was distilled from potassium hydroxide. Pleuromutilin was purchased as a commercial compound from TRC Canada. Analytical thin layer chromatography was performed Supelco 0.25 mm silica gel 60 F_{254} plates. Visualization was accomplished by irradiation with a 254 nm UV lamp or by staining with an aqueous solution of ceric ammonium molybdate, an acidified ethanolic solution of *p*-anisaldehyde, or a basified solution of potassium permanganate. Chromatography was performed using a forced flow of the indicated solvent system on SiliCycle SiliaFlash P60 silica gel (40-63 µm) or via automated flash chromatography using a Biotage Selekt system with SiliCycle SiliaSep or Teledyne RediSepRf gold chromatography columns. Deionized water was obtained from the house deionized water system.

^{1}H NMR spectra were recorded on a Bruker Avance II 500 MHz spectrometer or an Agilent U4-DD2 400 MHz spectrometer. Chemical shifts are reported in parts per million from tetramethylsilane (0 ppm) using the solvent resonance as an internal standard ($CDCl_3$ 7.26 ppm). Data are reported as follows: chemical shift, multiplicity (s=singlet, d=doublet, t=triplet, q=quartet, m=multiplet, br=broad), coupling constants, and number of protons. Proton decoupled ^{13}C NMR were recorded on a Bruker Avance II 500 MHz (126 MHz) spectrometer or an Agilent U4-DD2 400 MHz (101 MHz) spectrometer. Chemical shifts are reported in ppm from tetramethylsilane (0 ppm) using the solvent resonance as an internal standard ($CDCl_3$ 7.26 ppm). High resolution mass spectra were obtained on an Agilent Technologies 6220 TOF LC/MS or a Waters Synapt Q-TOF G2 or Thermo Exploris 120 HESI Orbitrap MS in the Department of Chemistry and the VT-Mass Spectrometry Incubator at the Virginia Polytechnic Institute and State University. Specific rotations were obtained on a Jasco P-2000 polarimeter.

<u>Broth Microdilution Assays</u>

Media and solutions were autoclaved or sterile filtered prior to use and manipulations were carried out in a laminar flow hood. Antibacterial testing was performed in polypropylene 96-well flat bottom plates in triplicate. The minimal inhibitory concentration (MIC) of pleuromutilin derivatives was assessed against *Staphylococcus aureus* DHS 8810, methicillin-resistant *S. aureus* (MRSA) ATCC 43300, *Enterococcus faecalis* ATCC 29212, vancomycin-resistant *Enterococcus*, *Escherichia coli* ΔTolC, *E. coli* MC1061, *Klebsiella pneumoniae* ATCC 29665, and *Acinetobacter baumannii* ATCC 17978, using serial dilutions. Pleuromutilin was used as a positive control and the vehicle as a negative control. Stock solutions of each compound were prepared at 8 mM in DMSO and serially diluted in DMSO to create master plates. From the master plates, 5 µL of each dilution was applied to test plates using a Bravo automated liquid transfer system (Agilent). Bacteria previously grown overnight (37 °C, 120 – 200 RPM), in LB broth were diluted to an OD600 of 0.05 in cation-adjusted Mueller-Hinton broth, grown to an OD600 of ~0.5, and diluted to an OD600 of 0.004 in cation-adjusted Mueller-Hinton broth. These diluted cultures were applied to the test plates (195 µL per test well) resulting in final concentrations of the test compounds of 200-0.0977 µM. The plates were incubated at 37 °C for 16–18 h, except for VRE and *E. faecalis*, which were incubated at 37 °C for 24 h. Inhibition was determined by measuring the optical density at 600 nm (OD600) with a

Cytation 3 plate reader (BioTek). The optical density measurements were normalized to the positive and negative controls. The MIC was the lowest concentration that inhibited >90% of growth.

Solubility Determination

Sample preparation for determination of solubility was as described in the Broth Microdilution Assay section above. The compounds were serially diluted in DMSO and the dilutions were transferred (5 µL) to 96-well plates and diluted (200 µL) with bacterial seeded cation-adjusted Mueller-Hinton broth. The concentrations of the test compounds on the plates were from 200-0.0977 µM. The plates were incubated at 37 °C for 16-24 hours depending on the test organism. OD600 measurements were taken on a Cytation 3 plate reader (BioTek). Solubility of the compounds was determined by visually assessing where precipitation for high-concentration wells ended for each compound and reporting a range based on the lowest-concentration well with precipitation and its neighboring well with no precipitation.

Syntheses and Characterizations of Compounds:

General Method A, Preparation of Monotosylate Hydroxy OEGs: To a round-bottomed flask equipped with a stirbar was added CH_2Cl_2 (0.2 M), triethylamine (2.1 equiv.) and an oligoethylene glycol (4 equiv.). With stirring, *p*-toluenesulfonyl chloride (1 equiv.) was added in one portion. The solution was allowed to stir for 12 h. The reaction was washed with aqueous solutions of 1 M $KHSO_4$ (50 mL) and saturated $NaHCO_3$ (50 mL). The organic layer was dried with Na_2SO_4 and concentrated. The residue was purified using column chromatography (SiO_2) with the indicated solvent mobile phase to afford the specific monotosylate hydroxy OEG.

General Method B, Preparation of Azido Hydroxy OEGs: Caution! Extended exposure of basic azides to dichloromethane can generate potentially explosive diazidomethane. All halogenated solvent should be scrupulously removed prior to creating the azido hydroxy OEGs. When using NaN_3, a Teflon spatula should be used to avoid sparking, acidic solutions for extraction should not be used to avoid generating toxic HN_3 gas, and care should be taken during weighing to avoid breathing NaN_3 dust. To a round-bottomed flask equipped with a stirbar and a reflux condenser was added a monotosylate hydroxy OEG prepared using Method A (1 equiv.), NaN_3 (2 equiv.), acetone (4 mL) and water (4 mL). With stirring, the mixture was brought to reflux and stirred for 15 h. The reaction was diluted with aqueous saturated $NaHCO_3$ (7 mL), and extracted with EtOAc (3x20 mL). The combined organics were dried with Na_2SO_4 and concentrated to afford the specific azido hydroxy OEG.

General Method C, Preparation of Azido Tosylate OEGs: To a round-bottomed flask equipped with a stirbar was added an azido hydroxy OEG prepared using Method B (1 equiv.), CH_2Cl_2 (20 mL), triethylamine (2 equiv.), and tosyl chloride (1.15 equiv.). The mixture was stirred at rt for 24 h. The reaction was quenched with 1 M aqueous $KHSO_4$ (20 mL), the layers were separated, and the organic layer was washed with saturated aqueous $NaHCO_3$ (20 mL), dried with Na_2SO_4, and concentrated to afford the specific azido tosylate OEG.

General Method D, Preparation of Azido Thioacetate OEGs: To a round-bottomed flask equipped with a stirbar and was added an azido tosylate OEG prepared using Method C (1 equiv.), acetone (25 mL), and KSAc (1.4 equiv.). The mixture was stirred at rt for 9 h. The mixture was concentrated and the resulting oily salt was digested with CH_2Cl_2 (15 mL). The suspension was filtered through Celite (CH_2Cl_2), and the filtrate was concentrated to afford the specific azido thioacetate OEG.

General Method E, Preparation of Hydroxy Thioacetate OEGs: To a round-bottomed flask equipped with a stirbar was added a monotosyl hydroxy OEG prepared using Method A (1 equiv.), acetone (25 mL), and potassium thioacetate (1.5 equiv.). The mixture was stirred for 24 h. The mixture was concentrated and the resulting oily salt was triturated in CH_2Cl_2 (20 mL). The suspension was filtered through Celite (CH_2Cl_2), and the filtrate was concentrated to afford the specific hydroxy thioacetate OEG.

2-(2-Hydroxyethoxy)ethyl 4-methylbenzenesulfonate (8): Prepared from **4** according to the general method A (2.00 g TsCl, 10.5 mmol) and purified using automated flash chromatography (SiliCycle 40g SiO_2 column, 0-10% CH_3OH in CH_2Cl_2) to give **8** (1.46 g, 54%) as a colorless oil: ^1H NMR (500 MHz, $CDCl_3$) δ 7.83 – 7.81 (m, 2H), 7.39 – 7.35 (m, 2H), 4.22 – 4.20 (m, 2H), 3.72 – 3.66 (m, 4H), 3.56 – 3.53 (m, 2H), 2.46 (s, 3H), 2.08 (s, 1H); $^{13}C\{^1H\}$ NMR (126 MHz, $CDCl_3$) δ 145.0, 133.0, 129.9, 127.0, 72.5, 69.2, 68.6, 61.6, 21.7; HRMS (ESI) m/z: $[M + Na]^+$ Calcd for $C_{13}H_{20}O_6SNa$ 283.0611; Found 283.0612.

2-(2-(2-Hydroxyethoxy)ethoxy)ethyl 4-methylbenzenesulfonate (9): Prepared from **5** according to the general method A (2.02 g TsCl, 10.6 mmol) and purified using automated flash chromatography (SiliCycle 40g SiO_2 column, 4% CH_3OH in CH_2Cl_2) to give **9** (2.64 g, 82%) as a colorless oil: ^1H NMR (400 MHz, $CDCl_3$) δ 7.80 (dt, *J* = 8.6, 2.0 Hz, 2H), 7.36 – 7.32 (m, 2H), 4.18 – 4.15 (m, 2H), 3.73 – 3.68 (m, 4H), 3.61 (br s, 4H), 3.59 – 3.56 (m, 2H), 2.44 (s, 3H), 2.08 (br s, 1H); $^{13}C\{^1H\}$ NMR (101 MHz, $CDCl_3$) δ 145.0, 133.1, 130.0, 128.1, 72.6, 70.9, 70.5, 69.3, 68.8, 61.9, 21.8; HRMS (ESI) m/z: $[M + Na]^+$ Calcd for $C_{13}H_{20}O_6SNa$ 327.0872; Found 327.0869.

2-(2-(2-(2-Hydroxyethoxy)ethoxy)ethoxy)ethyl 4-methylbenzenesulfonate (10): Prepared from **6** according to the general method A (2.02 g TsCl, 10.6 mmol) and purified using automated flash chromatography (SiliCycle 40g SiO_2 column, 4% CH_3OH in CH_2Cl_2) to give **10** (2.29 g, 63%) as a colorless oil: ^1H NMR (500 MHz, $CDCl_3$) δ 7.83 – 7.79 (m, 2H), 7.35 (d, *J* = 8.1 Hz, 2H), 4.19 – 4.14 (m, 2H), 3.73 – 3.68 (m, 4H), 3.68 – 3.62 (m, 4H), 3.62 – 3.59 (m, *J* = 4.1 Hz, 6H), 2.49 (s, 1H), 2.45 (s, 3H); $^{13}C\{^1H\}$ NMR (126 MHz, $CDCl_3$) δ 144.8, 132.9, 129.8, 128.0, 72.5, 70.7, 70.6, 70.5, 70.3, 69.3, 68.7, 61.7, 21.6; HRMS (ESI) m/z: $[M + Na]^+$ Calcd for $C_{15}H_{24}O_7SNa$ 371.1134; Found 371.1144.

14-Hydroxy-3,6,9,12-tetraoxatetradecyl 4-methylbenzenesulfonate (11): Prepared from **7** according to the general method A (1.00 g TsCl, 5.25 mmol) and purified using automated flash chromatography (SiliCycle 40g SiO_2 column, 4% CH_3OH in CH_2Cl_2) to give **11** (1.46 g, 71%) as a colorless oil: ^1H NMR (500 MHz, $CDCl_3$) δ 7.82 – 7.79 (m, 2H), 7.35 (d, *J* = 8.0 Hz, 2H), 4.17 (dd, *J* = 5.5, 4.2 Hz, 2H), 3.74 – 3.60 (m, 14H), 3.60 (s, 4H), 2.71 (t, *J* = 6.2 Hz, 1H), 2.46 (s, 3H); $^{13}C\{^1H\}$ NMR (126 MHz, $CDCl_3$) δ 144.9, 133.0, 129.9, 128.1, 72.6, 70.8, 70.68, 70.66, 70.62, 70.58, 70.4, 69.4, 68.8, 61.8, 21.8; HRMS (ESI) m/z: $[M + Na]^+$ Calcd for $C_{17}H_{28}O_8SNa$ 415.1397; Found 415.1395.

2-(2-Azidoethoxy)ethan-1-ol (12): Prepared from **8** (1.33 g, 5.11 mmol) according to the general method B to give **12** (0.450 g, 67%) as a colorless oil. Spectral data were in accord with those previously reported.[1]

2-(2-(2-Azidoethoxy)ethoxy)ethan-1-ol (13): Prepared from **9** (2.64 g, 8.67 mmol) according to the general method B to give **13** (0.886 g, 58%) as a colorless oil. Spectral data were in accord with those previously reported.[1]

2-(2-(2-(2-Azidoethoxy)ethoxy)ethoxy)ethan-1-ol (14): Prepared from **10** (2.16 g, 6.20 mmol) according to the general method B to give **14** (0.966 g, 71%) as a colorless oil. Spectral data were in accord with those previously reported.[1]

14-Azido-3,6,9,12-tetraoxatetradecan-1-ol (15): Prepared from **11** (1.60 g, 4.065 mmol) according to the general method B to give **15** (0.763 g, 71% yield) as a colorless oil. Spectral data were in accord with those previously reported.[2]

2-(2-Azidoethoxy)ethyl 4-methylbenzenesulfonate (16): Prepared from **12** (0.41 g, 3.13 mmol) according to the general method C and purified using automated flash chromatography (SiliCycle 40g SiO_2 column, 12-40-80% EtOAc in hexanes)to give **16** (0.490 g, 50%) as a yellow oil, and used in the next step without purification. Spectral data were in accord with those previously reported.[2]

2-(2-(2-Azidoethoxy)ethoxy)ethyl 4-methylbenzenesulfonate (17): Prepared from **13** (0.866 g, 5.1 mmol) according to the general method C and purified using automated flash chromatography (SiliCycle 40g SiO_2 column, 12-40-80% EtOAc in hexanes)to give **17** (1.00 g, 60%) as a yellow oil, and used in the next step without purification. Spectral data were in accord with those previously reported.[2]

2-(2-(2-(2-Azidoethoxy)ethoxy)ethoxy)ethyl 4-methylbenzenesulfonate (18): Prepared from **14** (0.966 g, 4.4 mmol) according to the general method C and purified using automated flash chromatography (SiliCycle 40g SiO_2 column, 12-55-100% EtOAc in hexanes)to give **18** (1.30 g, 79%) as a yellow oil, and used in the next step without purification. Spectral data were in accord with those previously reported.[2]

14-Azido-3,6,9,12-tetraoxatetradecyl 4-methylbenzenesulfonate (19): Prepared from **15** (0.763 g, 2.90 mmol) according to the general method C and purified using automated flash chromatography (SiliCycle 40g SiO_2 column, 0-10% CH_3OH in CH_2Cl_2) to give **19** (0.979 g, 90%) as a yellow oil, and used in the next step without purification. Spectral data were in accord with those previously reported.[2]

***S*-(2-(2-Azidoethoxy)ethyl) ethanethioate (20)**: Prepared from **16** (0.490 g, 1.72 mmol) according to the general method D and purified using automated flash chromatography (SiliCycle 40g SiO_2 column, 12-100% EtOAc in Hexanes) to give **20** (0.220 g, 66%) as a yellow oil. Spectral data were in accord with those previously reported.[3]

***S*-(2-(2-(2-Azidoethoxy)ethoxy)ethyl) ethanethioate (21)**: Prepared from **17** (1.00 g, 3.0 mmol) according to the general method D and purified using automated flash chromatography (SiliCycle 40g SiO_2 column, 12-100% EtOAc in Hexanes) to give **21** (0.419 g, 59%) as a yellow oil. ^{1}H NMR (400 MHz, $CDCl_3$) δ 3.67 – 3.64 (m, 2H), 3.63 – 3.61 (m, 4H), 3.59 (t, *J* = 6.4 Hz, 2H), 3.39 – 3.34 (m, 2H), 3.07 (t, *J* = 6.4 Hz, 2H), 2.31 (s, 3H); $^{13}C\{^1H\}$ NMR (101 MHz, $CDCl_3$) δ 195.6, 70.7, 70.5, 70.2, 69.9, 50.8, 30.7, 29.0; HRMS (ESI) m/z: $[M + Na]^+$ Calcd for $C_8H_{15}N_3O_3SNa$ 256.0726; Found 256.0728.

***S*-(2-(2-(2-(2-Azidoethoxy)ethoxy)ethoxy)ethyl) ethanethioate (22)**: Prepared from **18** (1.30 g, 3.48 mmol) according to the general method D and purified using automated flash chromatography (SiliCycle 40g SiO_2 column, 12-100% EtOAc in Hexanes) to give **22** (0.719 g, 74%) as a yellow oil. ^{1}H NMR (400 MHz, $CDCl_3$) δ 3.68 – 3.66 (m, 2H), 3.65 (br s, 4H), 3.64 – 3.60 (m, 4H), 3.59 (t, *J* = 6.4 Hz, 2H), 3.38 (dd, *J* = 5.6, 4.6 Hz, 2H), 3.08 (t, *J* = 6.5 Hz, 2H), 2.32 (s, 3H); $^{13}C\{^1H\}$ NMR (101 MHz, $CDCl_3$) δ 195.6, 70.8 (2C), 70.7, 70.4, 70.2, 69.9, 50.8, 30.7, 28.9; HRMS (ESI) m/z: $[M + Na]^+$ Calcd for $C_{10}H_{19}N_3O_4SNa$ 300.0988; Found 300.0994.

***S*-(14-Azido-3,6,9,12-tetraoxatetradecyl) ethanethioate (23)**: Prepared from **19** (0.979 g, 2.35 mmol) according to the general method D and purified using automated flash chromatography (SiliCycle 40g SiO_2 column, 20-100% EtOAc/Hexanes) to give **23** (0.483 g, 64%) as a yellow oil. ^{1}H NMR (400 MHz, $CDCl_3$) δ 3.68 – 3.66 (m, 2H), 3.66 – 3.64 (m, 8H), 3.64 – 3.61 (m, 4H), 3.59 (t, *J* = 6.5 Hz, 2H), 3.38 (dd, *J* = 5.5, 4.6

Hz, 2H), 3.08 (t, *J* = 6.5 Hz, 2H), 2.32 (s, *J* = 0.6 Hz, 3H); $^{13}C\{^1H\}$ NMR (101 MHz, $CDCl_3$) δ 195.6, 70.83, 70.80, 70.77 (2C), 70.6, 70.4, 70.2, 69.9, 50.8, 30.7, 29.0; HRMS (ESI) m/z: $[M + Na]^+$ Calcd for $C_{12}H_{23}N_3O_5SNa$ 344.1250; Found 344.1253.

(3a*R*,4*R*,5*R*,7*S*,8*S*,9*R*,9a*S*,12*R*)-8-Hydroxy-4,7,9,12-tetramethyl-3-oxo-7-vinyldecahydro-4,9a-propanocyclopenta[8]annulen-5-yl 2-(tosyloxy)acetate (24): Synthesis and characterization as previously reported.[4]

(3a*R*,4*R*,5*R*,7*S*,8*S*,9*R*,9a*S*,12*R*)-8-Hydroxy-4,7,9,12-tetramethyl-3-oxo-7-vinyldecahydro-4,9a-propanocyclopenta[8]annulen-5-yl 2-((2-hydroxyethyl)thio)acetate (25): To a N_2 sparged, 1.0 M solution of *t*-BuOK in THF (1.00 mL, 1.00 mmol), freshly distilled β-mercaptoethanol (0.132 mL, 1.88 mmol) was added resulting in a white suspension. After stirring for 30 min, a N_2 sparged solution of **24** (0.5065 mg, 0.9508 mmol) in methyl ethyl ketone (4.0 mL + 0.3 mL rinse) was added. The white suspension was stirred overnight. The mixture was diluted with one third saturated aqueous Na_2CO_3 (10 mL) and extracted with CH_2Cl_2 (3x20 mL). The combined organic layers were dried with Na_2SO_4 and concentrated. The residue was purified using automated flash chromatography (SiliCycle 25g SiO_2 column, 22-31% EtOAc in CH_2Cl_2) to give **25** (248 mg, 60%) as a colorless oil: ^1H NMR (500 MHz, $CDCl_3$) δ 6.46 (dd, *J* = 17.4, 11.0 Hz, 1H), 5.75 (d, *J* = 8.5 Hz, 1H), 5.35 (dd, *J* = 11.0, 1.5 Hz, 1H), 5.20 (dd, *J* = 17.4, 1.6 Hz, 1H), 3.76 – 3.72 (m, 2H), 3.35 (dd, *J* = 10.8, 6.6 Hz, 1H), 3.18 (s, 1H), 3.18 (s, 1H), 2.78 (dd, *J* = 6.1, 5.2 Hz, 2H), 2.7 – 2.68 (m, 1H), 2.33 (p, *J* = 6.9 Hz, 1H), 2.30 – 2.14 (m, 2H), 2.12 – 2.05 (m, 2H), 1.79 – 1.73 (m, 1H), 1.69 – 1.61 (m, 2H), 1.58 – 1.51 (m, 1H), 1.49 (dd, *J* = 10.7, 2.3 Hz, 1H), 1.48 – 1.42 (m, 1H), 1.44 (s, 3H), 1.39 – 1.34 (m, 1H), 1.31 (d, *J* = 16.1 Hz, 1H), 1.16 (s, 3H), 1.12 (td, *J* = 14.2, 4.6 Hz, 1H), 0.87 (d, *J* = 7.0 Hz, 3H) , 0.72 (d, *J* = 7.0 Hz, 3H); $^{13}C\{^1H\}$ NMR (126 MHz, $CDCl_3$) δ 217.3, 169.8, 139.0, 117.5, 74.7, 69.8, 60.6, 58.2, 45.6, 44.9, 44.0, 41.8, 36.8, 36.4, 36.1, 34.6, 34.4, 30.5, 26.9, 26.4, 24.9, 17.0, 15.0, 11.7; HRMS (ESI) m/z: $[M + Na]^+$ Calcd for $C_{24}H_{38}O_5SNa$ 461.2332; Found 461.2339; $[\alpha]_D^{27}$ = +23.4 (*c* 1.13, CH_3OH).

(3a*R*,4*R*,5*R*,7*S*,8*S*,9*R*,9a*S*,12*R*)-8-Hydroxy-4,7,9,12-tetramethyl-3-oxo-7-vinyldecahydro-4,9a-propanocyclopenta[8]annulen-5-yl 2-((2-azidoethyl)thio)acetate (28): To a stirring solution of **25** (0.404 g, 0.922 mmol) and tosyl chloride (0.246 g, 1.29 mmol) in CH_2Cl_2 (3.0 mL), triethylamine (0.260 mL, 1.86 mmol) was added. The mixture was stirred overnight. The mixture was diluted with CH_2Cl_2 (10 mL), washed sequentially with 0.3 M aqueous $KHSO_4$ (10 mL), saturated aqueous $NaHCO_3$ (10 mL), and brine (10 mL), dried with Na_2SO_4, and concentrated to afford **26** as an off-white amorphous solid (451 mg, 82% yield). This solution of **26** (451 mg, 0.760 mmol) and NaN_3 (80.2 mg, 1.23 mmol) in acetone (4 mL) and water (1.5 mL) was heated at reflux for 4.5 h. The mixture was cooled and concentrated. The residue was dissolved in EtOAc, washed with water (2x10 mL) and brine (5 mL), dried over Na_2SO_4, and concentrated. The residue was purified using automated flash chromatography (SiliCycle 12 g SiO_2 column, 40% EtOAc in hexanes) to give **28** (256 mg, 73%) as a colorless oil (By NMR, compound **28** exists as two rotomeric forms in a 4:1 ratio, believed to be due to favorable orbital interactions of the azide with the alkene.[5] ^1H NMR peaks have been reported as decimal amounts where peaks are resolvable, and ^{13}C NMR peaks from the rotomer have been marked with an asterisk): ^1H NMR (500 MHz, $CDCl_3$) δ 6.50 (dd, *J* = 17.4, 11.0 Hz, 0.8H), 6.49 (dd, *J* = 17.3, 11.0 Hz, 0.2H) 5.78 (d, *J* = 8.4, Hz, 1H), 5.382 (dd, *J* = 11.0, 1.6 Hz, 0.2H) 5.378 (dd, *J* = 11.0, 1.5 Hz, 0.8H), 5.24 (dd, *J* = 17.4, 1.5 Hz, 1H), 3.69 – 3.64 (m, 0.4H), 3.51 (td, *J* = 6.8, 1.3 Hz, 1.6H), 3.38 (d, *J* = 6.6 Hz, 1H), 3.21 (s, 1.6H), 3.20 (s, 0.4H), 2.99 – 2.95 (m, 0.4H), 2.84 – 2.81 (m, 1.6H), 2.37 (m, 1H), 2.32 – 2.17 (m, 2H), 2.15 – 2.09 (m, 2H), 1.80 (dq, *J* = 14.5, 3.2 Hz, 1H), 1.73 – 1.64 (m, 2H), 1.62 – 1.53 (m, 2H), 1.51 – 1.45 (m, 1H), 1.47 (s, 3H), 1.43 – 1.37 (m, 1H), 1.342 (d, *J* = 16.1 Hz, 0.8H), 1.340 (d, *J* = 16.1 Hz, 0.2H), 1.20 (s, 3H), 1.16 (td, *J* = 14.2, 4.5 Hz, 1H), 0.90 (d, *J* = 7.0 Hz, 3H), 0.758 (dd, *J* = 7.0 Hz,

2.4H), 0.753 (dd, J = 7.0 Hz, 0.6H); $^{13}C\{^1H\}$ NMR (101 MHz, $CDCl_3$) δ 217.0, 168.8, 139.2, 139.1*, 117.5*, 117.4, 74.8, 69.7*, 69.6, 58.3, 50.8, 45.6, 45.0, 44.1, 42.7*, 41.9, 36.9, 36.2, 34.8*, 34.60*, 34.58, 34.5, 31.9, 30.6, 27.0, 26.5, 25.0, 17.0, 15.0, 11.6; HRMS (ESI) m/z: $[M + Na]^+$ Calcd for $C_{24}H_{37}N_3O_4SNa$ 486.2397; Found 486.2390; $[\alpha]_D^{24}$ = +25.6 (c 4.28, CH_3OH).

(3a*R*,4*R*,5*R*,7*S*,8*S*,9*R*,9a*S*,12*R*)-8-Hydroxy-4,7,9,12-tetramethyl-3-oxo-7-vinyldecahydro-4,9a-propanocyclopenta[8]annulen-5-yl 2-((2-(2-azidoethoxy)ethyl)thio)acetate (29): A solution of **20** (0.209 g, 1.10 mmol) in MeOH (1.0 mL) was sparged with N_2 for 15 min and a sparged solution of 10 N NaOH in H_2O (0.11 mL, 1.1 mmol) was added. The mixture was stirred for 40 min, and then added to a stirring, sparged solution of **24** (0.533 g, 1.00 mmol) in methyl ethyl ketone (4.1 mL). The mixture stirred at 60 °C (oil bath) for 24 h. After cooling, the mixture was concentrated, the residue was taken up in water (15 mL) and EtOAc (15 mL), and the layers were separated. The aqueous layer was extracted with EtOAc (3x15 mL) and the combined organic layers were dried over Na_2SO_4 and concentrated. The residue was purified using automated flash chromatography (SiliCycle 12 g SiO_2 column, 30% EtOAc in hexanes) to give **29** (447 mg, 88%) as a colorless oil: ^1H NMR (500 MHz, $CDCl_3$) δ 6.48 (dd, J = 17.4, 11.0 Hz, 1H), 5.74 (d, J = 8.4 Hz, 1H), 5.34 (dd, J = 11.0, 1.6 Hz, 1H), 5.20 (dd, J = 17.4, 1.6 Hz, 1H), 3.68 (td, J = 6.5, 1.6 Hz, 2H), 3.63 (dd, J = 5.5, 4.6 Hz, 2H), 3.38 (dd, J = 5.6, 4.5 Hz, 2H), 3.36 – 3.33 (m, 1H), 3.21 (s, 2H), 2.81 (t, J = 6.4 Hz, 2H), 2.33 (p, J = 7.0 Hz, 1H), 2.30 – 2.14 (m, 2H), 2.11 – 2.05 (m, 2H), 1.76 (dq, J = 14.5, 3.1 Hz, 1H), 1.69 – 1.61 (m, 2H), 1.59 – 1.50 (m, 1H), 1.48 (br s, 1H), 1.48 – 1.42 (m, 1H), 1.45 (s, 3H), 1.39 – 1.34 (m, 1H), 1.32 (d, J = 16.1 Hz, 1H), 1.16 (s, 3H), 1.16 – 1.08 (m, 1H), 0.87 (d, J = 7.0 Hz, 3H), 0.73 (d, J = 7.0 Hz, 3H); $^{13}C\{^1H\}$ NMR (126 MHz, $CDCl_3$) δ 217.2, 169.0, 139.0, 117.3, 74.6, 70.7, 69.8, 69.2, 58.2, 50.7, 45.5, 44.7, 43.9, 41.8, 36.8, 36.0, 34.7, 34.5, 31.9, 30.4, 26.8, 26.3, 24.8, 16.9, 14.9, 11.6; HRMS (ESI) m/z: $[M + Na]^+$ Calcd for $C_{26}H_{41}N_3O_5SNa$ 530.2659; Found 530.2673; $[\alpha]_D^{24}$ = +23.8 (c 2.22, CH_3OH).

(3a*R*,4*R*,5*R*,7*S*,8*S*,9*R*,9a*S*,12*R*)-8-Hydroxy-4,7,9,12-tetramethyl-3-oxo-7-vinyldecahydro-4,9a-propanocyclopenta[8]annulen-5-yl 2-((2-(2-(2-azidoethoxy)ethoxy)ethyl)thio)acetate (30): A solution of **21** (0.263 g, 1.13 mmol) in MeOH (1.0 mL) was sparged with N_2 for 15 minutes and a sparged solution of 10 N NaOH in H_2O (0.11 mL, 1.1 mmol) was added. The mixture was stirred for 40 min and then added to a stirring, sparged solution of **24** (0.536 g, 1.00 mmol) in methyl ethyl ketone (4.1 mL). The mixture was heated to 60 °C (oil bath) and stirred for 24 h. After cooling, the mixture was concentrated. The residue was taken up in water (15 mL) and EtOAc (15 mL) and the layers were separated. The aqueous layer was extracted with EtOAc (3x15 mL) and the combined organic layers were dried over Na_2SO_4 and concentrated. The residue was purified using automated flash chromatography (SiliCycle 12 g SiO_2 column, 40% EtOAc in hexanes) to give **30** (477 mg, 86%) as a colorless oil. ^1H NMR (500 MHz, $CDCl_3$) δ 6.48 (dd, J = 17.4, 11.0 Hz, 1H), 5.75 (d, J = 8.5 Hz, 1H), 5.34 (dd, J = 11.0, 1.4 Hz, 1H), 5.20 (dd, J = 17.4, 1.3 Hz, 1H), 3.70 – 3.61 (m, 8H), 3.41 – 3.37 (m, 2H), 3.36 (d, J = 6.5 Hz, 1H), 3.21 (s, 2H), 2.82 – 2.79 (m, 2H), 2.34 (p, J = 7.0 Hz, 1H), 2.30 – 2.15 (m, 2H), 2.12 – 2.05 (m, 2H), 1.77 (dq, J = 14.6, 3.2 Hz, 1H), 1.70 – 1.62 (m, 2H), 1.61 – 1.50 (m, 2H), 1.48 – 1.42 (m, 1H), 1.45 (s, 3H), 1.37 (dq, J = 14.1, 3.5 Hz, 1H), 1.33 (d, J = 16.1 Hz, 1H), 1.17 (s, 3H), 1.13 (td, J = 14.2, 4.4 Hz, 1H), 0.87 (d, J = 7.0 Hz, 3H), 0.74 (d, J = 7.0, 3H); $^{13}C\{^1H\}$ NMR (126 MHz, $CDCl_3$) δ 217.3, 169.1, 139.2, 117.4, 74.7, 70.8 (2C), 70.5, 70.2, 69.3, 58.3, 50.8, 45.6, 44.9, 44.0, 41.9, 36.9, 36.1, 34.8, 34.6, 32.0, 30.5, 27.0, 26.5, 25.0, 17.0, 15.0, 11.6; HRMS (ESI) m/z: $[M + Na]^+$ Calcd for $C_{28}H_{45}N_3O_6SNa$ 574.2921; Found 574.2922; $[\alpha]_D^{23}$ = +22.7 (c 1.57, CH_3OH).

(3a*R*,4*R*,5*R*,7*S*,8*S*,9*R*,9a*S*,12*R*)-8-Hydroxy-4,7,9,12-tetramethyl-3-oxo-7-vinyldecahydro-4,9a-propanocyclopenta[8]annulen-5-yl 1-azido-3,6,9-trioxa-12-thiatetradecan-14-oate (31): A solution of **22**

(0.310 g, 1.12 mmol) in MeOH (0.65 mL) was sparged with N_2 for 15 min and a sparged solution of 10 N NaOH in H_2O (0.11 mL, 1.1 mmol) was added. The mixture was stirred for 40 min and then added to a stirring, sparged solution of **24** (0.530 g, 0.995 mmol) in methyl ethyl ketone (4.1 mL). The mixture was heated to 60 °C (oil bath) and stirred for 24 h. After cooling, the mixture was concentrated, the residue was taken up in water (10 mL) and CH_2Cl_2 (15 mL), and the layers were separated. The aqueous layer was extracted with CH_2Cl_2 (1x15 mL) and EtOAc (15 mL). The combined organic layers were dried over Na_2SO_4 and concentrated. The residue was purified using automated flash chromatography (SiliCycle 12 g SiO_2 column, 50% EtOAc in hexanes) to give **31** (331 mg, 56%) as a colorless oil. ^{1}H NMR (500 MHz, $CDCl_3$) δ 6.48 (dd, *J* = 17.4, 11.0 Hz, 1H), 5.74 (d, *J* = 8.4 Hz, 1H), 5.34 (dd, *J* = 11.0, 1.6 Hz, 1H), 5.20 (dd, *J* = 17.4, 1.6 Hz, 1H), 3.69 – 3.60 (m, 12H), 3.39 (dd, *J* = 5.6, 4.6 Hz, 2H), 3.36 (d, *J* = 6.6 Hz, 1H), 3.21 (s, 2H), 2.80 (t, *J* = 6.5 Hz, 2H), 2.34 (p, *J* = 7.0 Hz, 1H), 2.30 – 2.15 (m, 2H), 2.11 – 2.05 (m, 2H), 1.77 (dq, *J* = 14.5, 3.1 Hz, 1H), 1.70 – 1.63 (m, 2H), 1.62 – 1.50 (m, 2H), 1.48 – 1.42 (m, 1H), 1.45 (s, 3H), 1.40 – 1.35 (m, 1H), 1.33 (d, *J* = 16.0 Hz, 1H), 1.17 (s, 3H), 1.16 – 1.09 (m, 1H), 0.87 (d, *J* = 7.0 Hz, 3H), 0.73 (d, *J* = 7.0 Hz, 3H). ^{13}C{^{1}H} NMR (126 MHz, $CDCl_3$) δ 217.2, 169.0, 139.0, 117.3, 74.6, 70.71, 70.67, 70.64, 70.56, 70.3, 70.1, 69.2, 58.2, 50.7, 45.5, 44.7, 43.9, 41.8, 36.8, 36.0, 34.7, 34.5, 31.9, 30.4, 26.8, 26.3, 24.8, 16.9, 14.9, 11.6; HRMS (ESI) m/z: $[M + Na]^+$ Calcd for $C_{30}H_{49}N_3O_7SNa$ 618.3183; Found 618.3181; $[\alpha]_D^{23}$ = +21.6 (*c* 1.70, CH_3OH).

(3a*R*,4*R*,5*R*,7*S*,8*S*,9*R*,9a*S*,12*R*)-8-Hydroxy-4,7,9,12-tetramethyl-3-oxo-7-vinyldecahydro-4,9a-propanocyclopenta[8]annulen-5-yl 1-azido-3,6,9,12-tetraoxa-15-thiaheptadecan-17-oate (32): A solution of **23** (0.176 g, 0.548 mmol) in MeOH (0.50 mL) was sparged with N_2 for 15 minutes and a solution of 2 N NaOH in EtOH (0.27 mL) was added. The mixture was stirred for 40 min, and then the solution was added to a stirring, sparged solution of **24** (0.266 g, 0.499 mmol) in methyl ethyl ketone (2.0 mL). After stirring at rt overnight, the mixture was concentrated, the residue was taken up in water (10 mL) and CH_2Cl_2 (10 mL), and the layers were separated. The aqueous layer was extracted with CH_2Cl_2 (3x10 mL). The combined organic layers were diluted with EtOAc (5 mL), dried over Na_2SO_4, and concentrated. The residue was purified using automated flash chromatography (SiliCycle 12 g SiO_2 column, 1%, then 3%, then 5% CH_3OH in CH_2Cl_2) to give **32** (157 mg, 49%) as a colorless oil. ^{1}H NMR (400 MHz, $CDCl_3$) δ 6.44 (dd, *J* = 17.4, 11.0 Hz, 1H), 5.71 (d, *J* = 8.5 Hz, 1H), 5.31 (dd, *J* = 11.0, 1.6 Hz, 1H), 5.17 (dd, *J* = 17.4, 1.6 Hz, 1H), 3.67 – 3.55 (m, 16H), 3.35 (dd, *J* = 5.6, 4.6 Hz, 2H), 3.37 – 3.31 (m, 1H), 3.17 (s, 2H), 2.76 (t, *J* = 6.6 Hz, 2H), 2.31 (p, *J* = 7.3 Hz, 1H), 2.27 – 2.19 (m, 1H), 2.19 – 2.11 (m, 1H), 2.09 – 2.01 (m, 2H), 1.74 (dq, *J* = 14.3, 3.0 Hz, 1H), 1.68 – 1.58 (m, 2H), 1.57 – 1.47 (m, 1H), 1.46 – 1.38 (m, 1H), 1.45 (br s, 1H), 1.42 (s, 3H), 1.37 – 1.30 (m, 1H), 1.30 (d, *J* = 1.60 Hz, 1H), 1.14 (s, 3H), 1.14 – 1.05 (m, 1H), 0.85 (d, *J* = 7.0 Hz, 3H), 0.70 (d, *J* = 6.9 Hz, 3H). ^{13}C{^{1}H} NMR (101 MHz, $CDCl_3$) δ 217.1, 169.0, 139.2, 117.3, 74.7, 70.80, 70.77, 70.73 (2C), 70.69, 70.6, 70.4, 70.1, 69.3, 58.3, 50.8, 45.6, 44.9, 44.0, 41.9, 36.9, 36.1, 34.8, 34.6, 32.0, 30.5, 27.0, 26.5, 24.9, 16.9, 15.0, 11.6; HRMS (ESI) m/z: $[M + Na]^+$ Calcd for $C_{32}H_{53}N_3O_8SNa$ 662.3446; Found 662.3451; $[\alpha]_D^{23}$ = +19.8 (*c* 2.91, CH_3OH).

17-Hydroxy-3,6,9,12,15-pentaoxaheptadecyl 4-methylbenzenesulfonate (35): Prepared from **33** according to the general method A (1.00 g TsCl, 5.25 mmol) and purified using automated flash chromatography (SiliCycle 40g SiO_2 column, 5% CH_3OH in CH_2Cl_2) to give **35** (1.84 g, 80%) as a colorless oil: ^{1}H NMR (500 MHz, $CDCl_3$) δ 7.80 (d, *J* = 8.3 Hz, 2H), 7.35 (d, *J* = 8.0 Hz, 2H), 4.17 – 4.15 (m, 2H), 3.72 (dd, *J* = 5.6, 4.0 Hz, 2H), 3.70 – 3.60 (m, 16H), 3.59 (br s, 4H), 2.64 (t, *J* = 6.2 Hz, 1H), 2.45 (s, 3H); ^{13}C{^{1}H} NMR (126 MHz, $CDCl_3$) δ 144.8, 133.0, 129.8, 128.0, 72.5, 70.7, 70.60 (2C), 70.56, 70.55, 70.53, 70.51, 70.3, 69.3, 68.7, 61.7, 21.6; HRMS (ESI) m/z: $[M + Na]^+$ Calcd for $C_{19}H_{32}O_9SNa$ 459.1659; Found 459.1665.

23-Hydroxy-3,6,9,12,15,18,21-heptaoxatricosyl 4-methylbenzenesulfonate (36): Prepared from **34** according to the general method A (0.250 g TsCl, 1.31 mmol) and purified using automated flash chromatography (SiliCycle 40g SiO_2 column, 6% CH_3OH in CH_2Cl_2) to give **36** (0.474 g, 69%) as a colorless oil: ^1H NMR (500 MHz, $CDCl_3$) δ 7.79 – 7.76 (m, 2H), 7.34 – 7.30 (m, 2H), 4.15 – 4.11 (m, 2H), 3.71 – 3.68 (m, 2H), 3.67 – 3.57 (m, 24H), 3.56 (s, 4H), 2.65 (t, *J* = 6.2 Hz, 1H), 2.43 (s, 3H); $^{13}C\{^1H\}$ NMR (126 MHz, $CDCl_3$) δ 144.9, 133.0, 129.9, 128.0, 72.6, 70.8, 70.7 (2C), 70.63 (7C), 70.58, 70.4, 69.3, 68.7, 61.8, 21.7; HRMS (ESI) m/z: $[M + Na]^+$ Calcd for $C_{19}H_{32}O_9SNa$ 547.2183; Found 547.2186.

***S*-(2-(2-Hydroxyethoxy)ethyl) ethanethioate (37)**: Prepared from **8** according to the general method E and purified using automated flash chromatography (SiliCycle 40g SiO_2 column, 50-100% EtOAc in Hexanes) to give **37** (240 mg, 76%) as a yellow oil. ^1H NMR (500 MHz, $CDCl_3$) δ 3.72 (q, *J* = 4.8 Hz, 2H), 3.62 (t, *J* = 6.3 Hz, 2H), 3.59 – 3.56 (m, 2H), 3.10 (t, *J* = 6.3 Hz, 2H), 2.34 (s, 3H), 2.34 – 2.32 (m, 1H); $^{13}C\{^1H\}$ NMR (126 MHz, $CDCl_3$) δ 195.7, 72.1, 69.7, 61.7, 30.7, 29.0; HRMS (ESI) m/z: $[M + Na]^+$ Calcd for $C_6H_{12}O_3SNa$ 187.0399; Found 187.0391.

***S*-(2-(2-(2-Hydroxyethoxy)ethoxy)ethyl) ethanethioate (38)**: Prepared from **9** according to the general method E and purified using automated flash chromatography (SiliCycle 40g SiO_2 column, 50-100% EtOAc in Hexanes) to give **38** (231 mg, 67%) as a yellow oil. ^1H NMR (500 MHz, $CDCl_3$) δ 3.73 (t, *J* = 4.4 Hz, 2H), 3.67 – 3.62 (m, 4H), 3.62 – 3.59 (m, 4H), 3.10 (t, *J* = 6.5 Hz, 2H), 2.57 (s, 1H), 2.34 (s, 3H); $^{13}C\{^1H\}$ NMR (126 MHz, $CDCl_3$) δ 195.7, 72.6, 70.41, 70.37, 69.8, 61.8, 30.6, 28.8; HRMS (ESI) m/z: $[M + Na]^+$ Calcd for $C_8H_{16}O_4SNa$ 231.0661; Found 231.0655.

***S*-(2-(2-(2-(2-Hydroxyethoxy)ethoxy)ethoxy)ethyl) ethanethioate (39)**: Prepared from **10** according to the general method E and purified using automated flash chromatography (SiliCycle 40g SiO_2 column, 12-100% acetone in EtOAc) to give **39** (284 mg, 78%) as a yellow oil. ^1H NMR (400 MHz, $cdcl_3$) δ 3.75 – 3.70 (m, 2H), 3.70 – 3.61 (m, 10H), 3.60 (t, *J* = 6.5 Hz, 2H), 3.09 (t, *J* = 6.5 Hz, 2H), 2.47 – 2.42 (m, 1H), 2.33 (s, 3H); $^{13}C\{^1H\}$ NMR (101 MHz, $cdcl_3$) δ 195.6, 72.6, 70.8, 70.6, 70.5, 70.4, 69.9, 61.8, 30.6, 28.9; HRMS (ESI) m/z: $[M + Na]^+$ Calcd for $C_{10}H_{20}O_5SNa$ 275.0924; Found 275.0924.

***S*-(14-Hydroxy-3,6,9,12-tetraoxatetradecyl) ethanethioate (40)**: Prepared from **11** according to the general method E and purified using automated flash chromatography (SiliCycle 40g SiO_2 column, 12-100% acetone in EtOAc) to give **40** (288 mg, 76%). ^1H NMR (400 MHz, $cdcl_3$) δ 3.73 – 3.68 (m, 2H), 3.68 – 3.59 (m, 14H), 3.58 (t, *J* = 6.4 Hz, 2H), 3.07 (t, *J* = 6.4 Hz, 2H), 2.62 (t, *J* = 6.2 Hz, 1H), 2.31 (s, 3H); $^{13}C\{^1H\}$ NMR (101 MHz, $cdcl_3$) δ 195.7, 72.6, 70.73 (2C), 70.69, 70.62, 70.5, 70.4, 69.9, 61.8, 30.7, 28.9; HRMS (ESI) m/z: $[M + Na]^+$ Calcd for $C_{12}H_{24}O_6SNa$ 319.1186; Found 319.1186.

***S*-(17-Hydroxy-3,6,9,12,15-pentaoxaheptadecyl) ethanethioate (41)**: Prepared from **35** according to the general method E and purified using automated flash chromatography (SiliCycle 40g SiO_2 column, 12-100% acetone in EtOAc) to give **41** (293 mg, 74%). ^1H NMR (400 MHz, $cdcl_3$) δ 3.70 (td, *J* = 6.0, 4.4 Hz, 2H), 3.67 – 3.59 (m, 18H), 3.57 (t, *J* = 6.5 Hz, 2H), 3.07 (t, *J* = 6.5 Hz, 2H), 2.65 (t, *J* = 6.2 Hz, 1H), 2.31 (s, 3H); $^{13}C\{^1H\}$ NMR (101 MHz, $cdcl_3$) δ 195.6, 72.6, 70.72, 70.71, 70.67 (2C), 70.65, 70.60, 70.44, 70.39, 69.8, 61.8, 30.6, 28.9; HRMS (ESI) m/z: $[M + H]^+$ Calcd for $C_{14}H_{28}O_7SNa$ 341.1628; Found 341.1629.

***S*-(23-Hydroxy-3,6,9,12,15,18,21-heptaoxatricosyl) ethanethioate (42)**: Prepared from **36** according to the general method E and purified using automated flash chromatography (SiliCycle 40g SiO_2 column, 12-100% acetone in EtOAc) to give **42** (195 mg, 53%) ^1H NMR (400 MHz, $CDCl_3$) δ 3.72 – 3.68 (m, 2H), 3.66 – 3.59 (m, 26H), 3.58 (t, *J* = 6.4 Hz, 2H), 3.07 (t, *J* = 6.4 Hz, 2H), 2.66 (t, *J* = 6.2 Hz, 1H), 2.32 (s, 3H); $^{13}C\{^1H\}$

NMR (101 MHz, cdcl$_3$) δ 195.6, 72.6, 70.73 (2C), 70.68 (6C), 70.65, 70.61, 69.9, 61.8, 30.7, 28.9; HRMS (ESI) m/z: $[M + Na]^+$ Calcd for $C_{18}H_{36}O_9SNa$ 451.1972; Found 451.1972.

(3a*R*,4*R*,5*R*,7*S*,8*S*,9*R*,9a*S*,12*R*)-8-Hydroxy-4,7,9,12-tetramethyl-3-oxo-7-vinyldecahydro-4,9a-propanocyclopenta[8]annulen-5-yl 2-((2-(2-hydroxyethoxy)ethyl)thio)acetate (43): A solution of **37** (86.4 mg, 0.526 mmol) in MeOH (0.5 mL) was sparged with N_2 for 15 min and a sparged solution of 10 N NaOH in H_2O (0.05 mL, 0.5 mmol) was added. The mixture was stirred for 1 h, and then added to a stirring, sparged solution of **24** (0.250 g, 0.470 mmol) in methyl ethyl ketone (2.0 mL). The mixture was heated to 60 °C (oil bath) and stirred for 24 h. The mixture was concentrated, the residue was taken up in water (5 mL) and EtOAc (10 mL), and the phases were separated. The aqueous layer was extracted with EtOAc (3x10 mL) and the combined organic layers were dried with Na_2SO_4 and concentrated. The residue was purified using automated flash chromatography (SiliCycle 12 g SiO_2 column, 50% EtOAc in hexanes) to give **43** (58.9 mg, 25%) as a colorless oil: ^{1}H NMR (500 MHz, CDCl$_3$) δ 6.47 (dd, *J* = 17.4, 11.0 Hz, 1H), 5.74 (d, *J* = 8.5 Hz, 1H), 5.34 (dd, *J* = 11.0, 1.6 Hz, 1H), 5.19 (dd, *J* = 17.4, 1.6 Hz, 1H), 3.72 – 3.70 (m, 2H), 3.68 (td, *J* = 6.2, 2.1 Hz, 2H), 3.55 (dd, *J* = 4.8, 3.9 Hz, 2H), 3.35 (dd, *J* = 10.2, 6.5 Hz, 1H), 3.23 (d, *J* = 14.8 Hz, 1H), 3.19 (d, *J* = 14.8 Hz, 1H), 2.79 (td, *J* = 6.2, 1.9 Hz, 2H), 2.37 (br s, 1H), 2.33 (p, *J* = 7.0 Hz, 1H), 2.29 – 2.23 (m, 1H), 2.22 – 2.13 (m, 1H), 2.11 – 2.05 (m, 2H), 1.76 (dq, *J* = 14.5, 3.2 Hz, 1H), 1.69 – 1.60 (m, 2H), 1.59 – 1.48 (m, 2H), 1.47 – 1.41 (m, 1H), 1.44 (s, 3H), 1.36 (dq, *J* = 14.1, 3.4 Hz, 1H), 1.32 (d, *J* = 16.1 Hz, 1H), 1.16 (s, 3H), 1.12 (td, *J* = 14.2, 4.6 Hz, 1H), 0.86 (d, *J* = 7.0 Hz, 3H), 0.72 (d, *J* = 7.0 Hz, 3H); ^{13}C{^{1}H} NMR (126 MHz, CDCl$_3$) δ 217.3, 169.2, 139.1, 117.4, 74.7, 72.2, 70.5, 69.4, 61.8, 58.3, 45.6, 44.8, 44.0, 41.8, 36.8, 36.1, 34.7, 34.6, 32.1, 30.5, 26.9, 26.4, 24.9, 17.0, 15.0, 11.7; HRMS (ESI) m/z: $[M + Na]^+$ Calcd for $C_{26}H_{42}O_6SNa$ 505.2594; Found 505.2597; $[\alpha]_D^{26}$ = +22.8 (*c* 1.11, CH_3OH).

(3a*R*,4*R*,5*R*,7*S*,8*S*,9*R*,9a*S*,12*R*)-8-Hydroxy-4,7,9,12-tetramethyl-3-oxo-7-vinyldecahydro-4,9a-propanocyclopenta[8]annulen-5-yl 2-((2-(2-(2-hydroxyethoxy)ethoxy)ethyl)thio)acetate (44): A solution of **38** (0.111 g, 0.532 mmol) in MeOH (0.50 mL) was sparged with N_2 for 15 minutes and a spared solution of 10 N NaOH in H_2O (0.052 mL, 0.52 mmol) was added. The mixture was stirred for 40 min, and then added to a stirring, sparged solution of **24** (0.254 g, 0.476 mmol) in methyl ethyl ketone (2.0 mL). The mixture was heated to 60 °C (oil bath) and stirred for 48 h. The mixture was concentrated, the residue was taken up in water (5 mL) and EtOAc (10 mL), and the layers were separated. The aqueous layer was extracted with EtOAc (3x10 mL) and the combined organics were dried over Na_2SO_4 and concentrated. The residue was purified using automated flash chromatography (SiliCycle 12 g SiO_2 column, 75% EtOAc in CH_2Cl_2) to give **44** (125.9 mg, 48%) as a colorless oil: ^{1}H NMR (500 MHz, CDCl$_3$) δ 6.46 (dd, *J* = 17.3, 11.1 Hz, 1H), 5.73 (d, *J* = 8.4 Hz, 1H), 5.33 (dd, *J* = 10.9, 1.6 Hz, 1H), 5.19 (dd, *J* = 17.4, 1.5 Hz, 1H), 3.73 – 3.70 (m, *J* = 4.9, 3.1, 1.8 Hz, 2H), 3.69 – 3.63 (m, 4H), 3.62 – 3.57 (m, 4H), 3.34 (t, *J* = 8.1 Hz, 1H), 3.21 (s, 2H), 2.79 (t, *J* = 6.5 Hz, 2H), 2.53 (br s, 1H), 2.33 (dt, *J* = 7.0 Hz, 1H), 2.29 – 2.13 (m, 2H), 2.10 – 2.04 (m, 2H), 1.75 (dq, *J* = 14.6, 3.2 Hz, 1H), 1.68 – 1.59 (m, 2H), 1.58 – 1.47 (m, 2H), 1.47 – 1.41 (m, 1H), 1.44 (s, 3H), 1.35 (dq, *J* = 14.2, 3.0 Hz, 1H), 1.31 (d, *J* = 16.1 Hz, 1H), 1.15 (s, 3H), 1.15 – 1.08 (m, 1H), 0.86 (dd, *J* = 7.0 Hz, 3H), 0.72 (dd, *J* = 7.0 Hz, 3H); ^{13}C{^{1}H} NMR (126 MHz, CDCl$_3$) δ 217.3, 169.1, 139.1, 117.4, 74.7, 72.6, 70.6, 70.41, 70.38, 69.4, 61.8, 58.3, 45.5, 44.8, 44.0, 41.8, 36.8, 36.1, 34.7, 34.6, 31.9, 30.5, 26.9, 26.4, 24.9, 17.0, 15.0, 11.6; HRMS (ESI) m/z: $[M + Na]^+$ Calcd for $C_{28}H_{46}O_7SNa$ 549.2856; Found 549.2855; $[\alpha]_D^{26}$ = +21.8 (*c* 1.31, CH_3OH).

(3a*R*,4*R*,5*R*,7*S*,8*S*,9*R*,9a*S*,12*R*)-8-Hydroxy-4,7,9,12-tetramethyl-3-oxo-7-vinyldecahydro-4,9a-propanocyclopenta[8]annulen-5-yl 1-hydroxy-3,6,9-trioxa-12-thiatetradecan-14-oate (45): A solution of

39 (0.1325 g, 0.525 mmol) in MeOH (0.50 mL) was sparged with N_2 for 15 minutes and a sparged solution of 10 N NaOH in H_2O (0.095 mL, 0.95 mmol) was added. The mixture was stirred for 15 min and then added to a stirring, sparged solution of **24** (0.253 g, 0.476 mmol) in methyl ethyl ketone (2.0 mL). The mixture was stirred at rt for 18 h, and then at 60 °C (oil bath) for 24 h. The mixture was concentrated, the residue was taken up in water (5 mL) and EtOAc (10 mL), and the layers were separated. The aqueous layer was extracted with EtOAc (4x10 mL) and the combined organic layers were washed with brine (10 mL), dried over Na_2SO_4 and concentrated. The residue was purified using automated flash chromatography (SiliCycle 12 g SiO_2 column, 5-10% CH_3OH in CH_2Cl_2) to give **45** (105.2 mg, 39%) as a colorless oil: ^{1}H NMR (500 MHz, $CDCl_3$) δ 6.46 (dd, *J* = 17.4, 11.0 Hz, 1H), 5.73 (d, *J* = 8.4 Hz, 1H), 5.33 (dd, *J* = 11.0, 1.5 Hz, 1H), 5.19 (dd, *J* = 17.4, 1.6 Hz, 1H), 3.73 – 3.69 (m, 2H), 3.68 – 3.57 (m, 12H), 3.34 (dd, *J* = 10.5, 6.5 Hz, 1H), 3.20 (s, 2H), 2.79 (t, *J* = 6.5 Hz, 2H), 2.75 (br s, 1H), 2.32 (p, *J* = 7.0 Hz, 1H), 2.29 – 2.14 (m, 2H), 2.11 – 2.03 (m, 2H), 1.75 (dq, *J* = 14.5, 3.1 Hz, 1H), 1.68 – 1.59 (m, 2H), 1.58 – 1.47 (m, 2H), 1.47 – 1.42 (m, 1H), 1.44 (s, 3H), 1.37 – 1.32 (m, 1H), 1.31 (d, *J* = 16.0 Hz, 1H), 1.15 (s, 3H), 1.15 – 1.08 (m, 1H), 0.86 (d, *J* = 7.0 Hz, 3H), 0.72 (d, *J* = 7.0 Hz, 3H); ^{13}C{^{1}H} NMR (126 MHz, $CDCl_3$) δ 217.3, 169.1, 139.1, 117.4, 74.7, 72.6, 70.7, 70.60, 70.57, 70.37, 70.35, 69.3, 61.8, 58.3, 45.5, 44.8, 44.0, 41.8, 36.9, 36.0, 34.7, 34.6, 31.9, 30.5, 26.9, 26.4, 24.9, 17.0, 15.0, 11.6; HRMS (ESI) m/z: $[M + Na]^+$ Calcd for $C_{30}H_{50}O_8SNa$ 593.3119; Found 593.3131; $[\alpha]_D^{26}$ = +21.0 (*c* 1.20, CH_3OH).

(3a*R*,4*R*,5*R*,7*S*,8*S*,9*R*,9a*S*,12*R*)-8-Hydroxy-4,7,9,12-tetramethyl-3-oxo-7-vinyldecahydro-4,9a-propanocyclopenta[8]annulen-5-yl 1-hydroxy-3,6,9,12-tetraoxa-15-thiaheptadecan-17-oate (46): A solution of **40** (0.153 g, 0.517 mmol) in THF (0.50 mL) was sparged with N_2 for 15 minutes and a sparged solution of 10 N NaOH in H_2O (0.095 mL, 0.95 mmol) was added. The mixture was stirred for 30 min and then added to a stirring, sparged solution of **24** (0.255 g, 0.479 mmol) in methyl ethyl ketone (2.0 mL). A noticeable amount of the sodium salt of deprotected **40** became insoluble in the THF/water mixture and failed to be transferred. The resulting mixture was stirred at 60 °C (oil bath) for 48 h. The mixture was concentrated, the residue was taken up in water (5 mL) and EtOAc (10 mL), and the layers were separated. The aqueous layer was extracted with EtOAc (4x10 mL) and the combined organics were washed with brine, dried over Na_2SO_4, and concentrated. The residue was purified using automated flash chromatography (SiliCycle 12 g SiO_2 column, 1-12% (20:1 CH_3OH:conc. $NH_4OH_{(aq)}$) in CH_2Cl_2) to give **46** (104.6 mg, 36%) as a colorless oil: ^{1}H NMR (500 MHz, $CDCl_3$) δ 6.48 (dd, *J* = 17.4, 11.0 Hz, 1H), 5.74 (d, *J* = 8.5 Hz, 1H), 5.34 (dd, *J* = 11.0, 1.5 Hz, 1H), 5.20 (dd, *J* = 17.5, 1.6 Hz, 1H), 3.72 (q, *J* = 4.9 Hz, 2H), 3.69 – 3.60 (m, 16H), 3.35 (dd, *J* = 10.8, 6.6 Hz, 1H), 3.21 (s, 2H), 2.79 (t, *J* = 6.5 Hz, 2H), 2.68 (t, *J* = 6.4 Hz, 1H), 2.37 – 2.31 (m, 1H), 2.30 – 2.15 (m, 2H), 2.11 – 2.05 (m, 2H), 1.77 (dq, *J* = 14.5, 3.2 Hz, 1H), 1.69 – 1.61 (m, 2H), 1.60 – 1.50 (m, 1H), 1.49 – 1.42 (m, 1H), 1.47 (s, 1H), 1.45 (s, 3H), 1.40 – 1.34 (m, 1H), 1.33 (d, *J* = 16.1 Hz, 1H), 1.17 (s, 3H), 1.16 – 1.09 (m, 1H), 0.87 (d, *J* = 7.0 Hz, 3H), 0.73 (d, *J* = 7.0 Hz, 3H); ^{13}C{^{1}H} NMR (126 MHz, $CDCl_3$) δ 217.3, 169.1, 139.1, 117.4, 74.7, 72.6, 70.72, 70.70, 70.68, 70.65 (2C), 70.4 (2C), 69.3, 61.9, 58.3, 45.6, 44.9, 44.0, 41.9, 36.9, 36.1, 34.8, 34.6, 32.0, 30.6, 27.0, 26.4, 25.0, 17.0, 15.0, 11.7; HRMS (ESI) m/z: $[M + Na]^+$ Calcd for $C_{32}H_{54}O_9SNa$ 637.3381; Found 637.3391; $[\alpha]_D^{26}$ = +22 (*c* 0.40, CH_3OH).

(3a*R*,4*R*,5*R*,7*S*,8*S*,9*R*,9a*S*,12*R*)-8-Hydroxy-4,7,9,12-tetramethyl-3-oxo-7-vinyldecahydro-4,9a-propanocyclopenta[8]annulen-5-yl 1-hydroxy-3,6,9,12,15-pentaoxa-18-thiaicosan-20-oate (47): To a solution of **41** (0.0750 g, 0.220 mmol) in N_2 sparged MeOH (0.20 mL) was added a sparged solution of 10 N NaOH in H_2O (0.095 mL, 0.95 mmol). The mixture was stirred for 45 min and then a sparged solution of **24** (0.1010 g, 0.1896 mmol) in methyl ethyl ketone (0.8 mL + 0.5 mL wash) was added resulting in a faintly yellow solution that was stirred at rt for 120 h. The mixture was concentrated, the residue was taken up

in water (5 mL) and EtOAc (10 mL), and the layers were separated. The aqueous layer was extracted with EtOAc (5x10 mL) and the combined organic layers were washed with brine, dried over Na_2SO_4, and concentrated. The residue was purified using automated flash chromatography (SiliCycle 12 g SiO_2 column, 1-12% (20:1 CH_3OH:conc. $NH_4OH_{(aq)}$) in CH_2Cl_2) to give **47** (63.5 mg, 51%) as a colorless oil: ^{1}H NMR (500 MHz, $CDCl_3$) δ 6.48 (dd, *J* = 17.4, 11.0 Hz, 1H), 5.75 (d, *J* = 8.5 Hz, 1H), 5.35 (dd, *J* = 11.0, 1.6 Hz, 1H), 5.20 (dd, *J* = 17.4, 1.6 Hz, 1H), 3.74 – 3.70 (m, 2H), 3.69 – 3.60 (m, 20H), 3.36 (dd, *J* = 10.9, 6.5 Hz, 1H), 3.21 (s, 2H), 2.80 (t, *J* = 6.6 Hz, 2H), 2.76 (br s, 1H), 2.37 – 2.31 (m, 1H), 2.30 – 2.16 (m, 2H), 2.12 – 2.05 (m, 2H), 1.77 (dq, *J* = 14.4, 2.9 Hz, 1H) 1.69 – 1.61 (m, 2H), 1.55 (qd, *J* = 13.3, 3.5 Hz, 1H), 1.48 – 1.42 (m, 1H), 1.47 (s, 1H), 1.45 (s, 3H) 1.40 – 1.35 (m, 1H), 1.33 (d, *J* = 16.1 Hz, 1H), 1.17 (s, 3H), 1.13 (td, *J* = 14.3, 4.6 Hz, 1H), 0.88 (d, *J* = 7.1 Hz, 3H), 0.73 (d, *J* = 7.0 Hz, 3H); $^{13}C\{^1H\}$ NMR (126 MHz, $CDCl_3$) δ 217.3, 169.1, 139.1, 117.4, 74.7, 72.7, 70.73 (2C), 70.68 (2C), 70.66, 70.65 (2C), 70.4 (2C), 69.3, 61.9, 58.3, 45.6, 44.9, 44.0, 41.9, 36.9, 36.1, 34.8, 34.6, 32.0, 30.6, 27.0, 26.5, 25.0, 17.0, 15.0, 11.7; HRMS (ESI) m/z: $[M + Na]^+$ Calcd for $C_{34}H_{58}O_{10}SNa$ 681.3643; Found 681.3640; $[\alpha]_D^{26}$ = +23 (*c* 0.25, CH_3OH).

(3a*R*,4*R*,5*R*,7*S*,8*S*,9*R*,9a*S*,12*R*)-8-Hydroxy-4,7,9,12-tetramethyl-3-oxo-7-vinyldecahydro-4,9a-propanocyclopenta[8]annulen-5-yl 1-hydroxy-3,6,9,12,15,18,21-heptaoxa-24-thiahexacosan-26-oate (48): To a solution of **42** (0.0890 g, 0.208 mmol) in N_2 sparged MeOH (0.20 mL) was added a sparged solution of 10 N NaOH in H_2O (0.040 mL, 0.40 mmol). The mixture was stirred for 1.5 h and then a sparged solution of **24** (0.1022 g, 0.1919 mmol) in methyl ethyl ketone (0.8 mL + 0.3 mL wash) was added resulting in a faintly yellow solution that was stirred at rt for 96 h. The mixture was concentrated, the residue was taken up in water (5 mL) and EtOAc (10 mL), and the layers were separated. The aqueous layer was extracted with EtOAc (7x10 mL) and the combined organic layers were washed with brine, dried over Na_2SO_4, and concentrated. The residue was purified using automated flash chromatography (SiliCycle 12 g SiO_2 column, 1-12% (20:1 CH_3OH:conc. $NH_4OH_{(aq)}$) in CH_2Cl_2) to give **48** (54.2 mg, 38%) as a colorless oil: ^{1}H NMR (500 MHz, $CDCl_3$) δ 6.48 (dd, *J* = 17.4, 11.0 Hz, 1H), 5.75 (d, *J* = 8.5 Hz, 1H), 5.35 (dd, *J* = 11.0, 1.6 Hz, 1H), 5.20 (dd, *J* = 17.4, 1.6 Hz, 1H), 3.74 – 3.70 (m, 2H), 3.68 – 3.59 (m, 28H), 3.36 (dd, *J* = 10.8, 6.5 Hz, 1H), 3.21 (s, 2H), 2.98 (br s, 1H), 2.79 (t, *J* = 6.6 Hz, 2H), 2.37 – 2.31 (m, 1H), 2.30 – 2.15 (m, 2H), 2.12 – 2.05 (m, 2H), 1.77 (dq, *J* = 14.5, 2.9 Hz, 1H), 1.67 – 1.62 (m, 2H), 1.55 (qd, *J* = 13.4, 3.6 Hz, 1H), 1.49 – 1.42 (m, *J* = 14.2 Hz, 2H), 1.45 (s, 3H), 1.40 – 1.35 (m, 1H), 1.33 (d, *J* = 16.1 Hz, 1H), 1.17 (s, 3H), 1.16 – 1.09 (m, 1H), 0.88 (d, *J* = 7.0 Hz, 3H), 0.73 (d, *J* = 7.0 Hz, 3H); $^{13}C\{^1H\}$ NMR (126 MHz, $CDCl_3$) δ 217.4, 169.1, 139.1, 117.4, 74.7, 72.7, 70.71 (2C), 70.67 (4C), 70.64 (4C), 70.61, 70.43, 70.36, 69.3, 61.8, 58.3, 45.6, 44.9, 44.0, 41.9, 36.9, 36.1, 34.8, 34.6, 32.0, 30.6, 27.0, 26.5, 25.0, 17.0, 15.0, 11.7; HRMS (ESI) m/z: $[M + Na]^+$ Calcd for $C_{38}H_{66}O_{12}SNa$ 769.4167; Found 769.4167; $[\alpha]_D^{27}$ = +29 (*c* 0.13, CH_3OH).

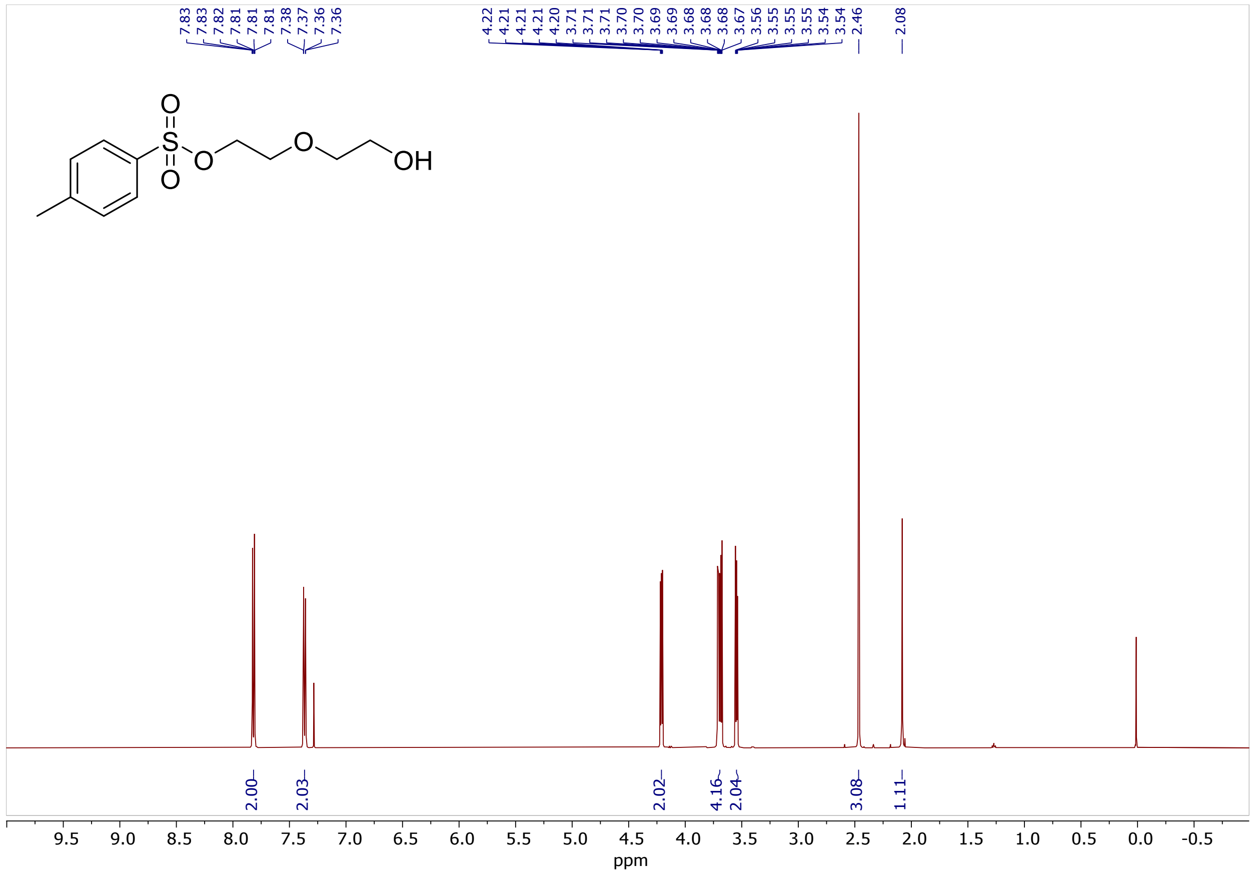

^{1}H-NMR (500 MHz, $CDCl_3$) spectrum of **8**

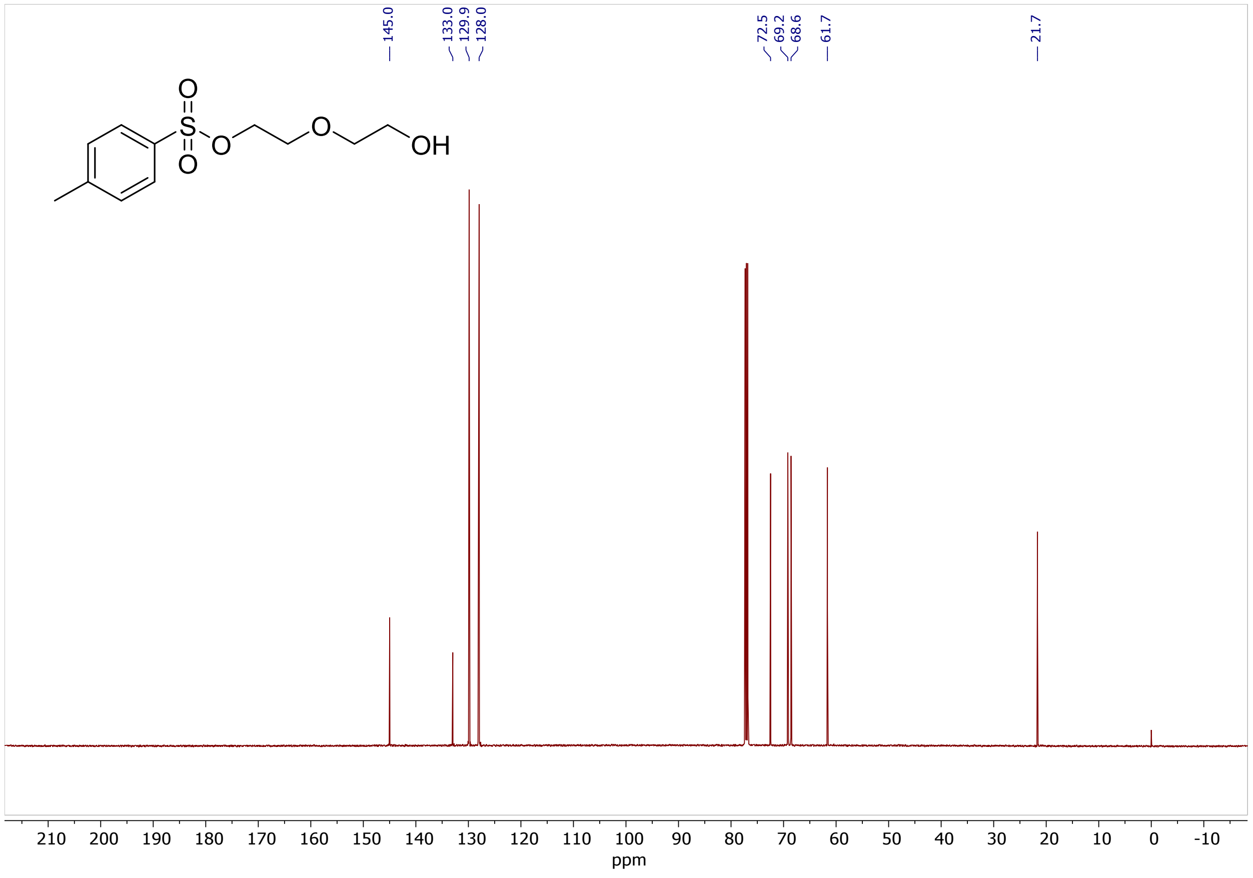

$^{13}C\{^{1}H\}$-NMR (126 MHz, $CDCl_3$) spectrum of **8**

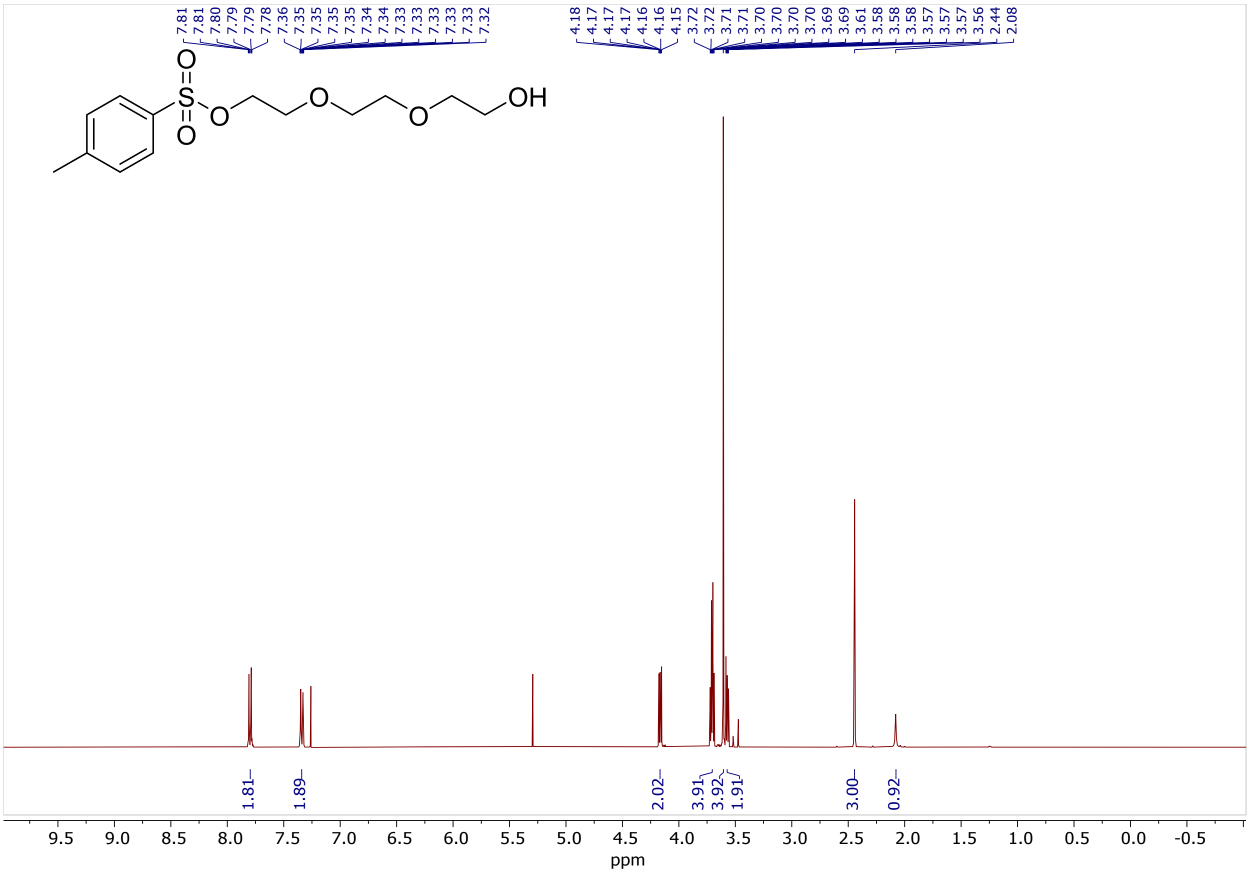

^{1}H-NMR (400 MHz, $CDCl_3$) spectrum of **9**

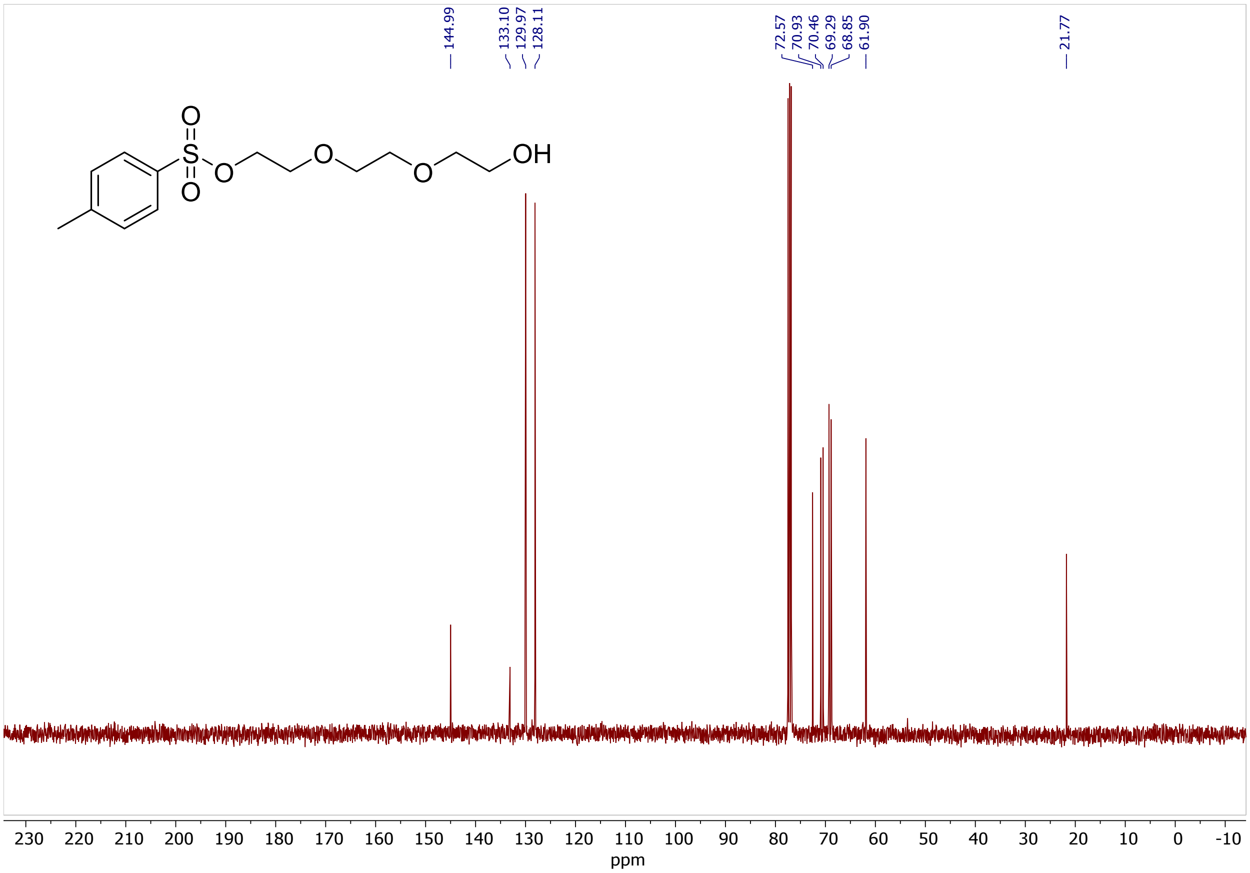

^{13}C{^{1}H}-NMR (101 MHz, $CDCl_3$) spectrum of **9**

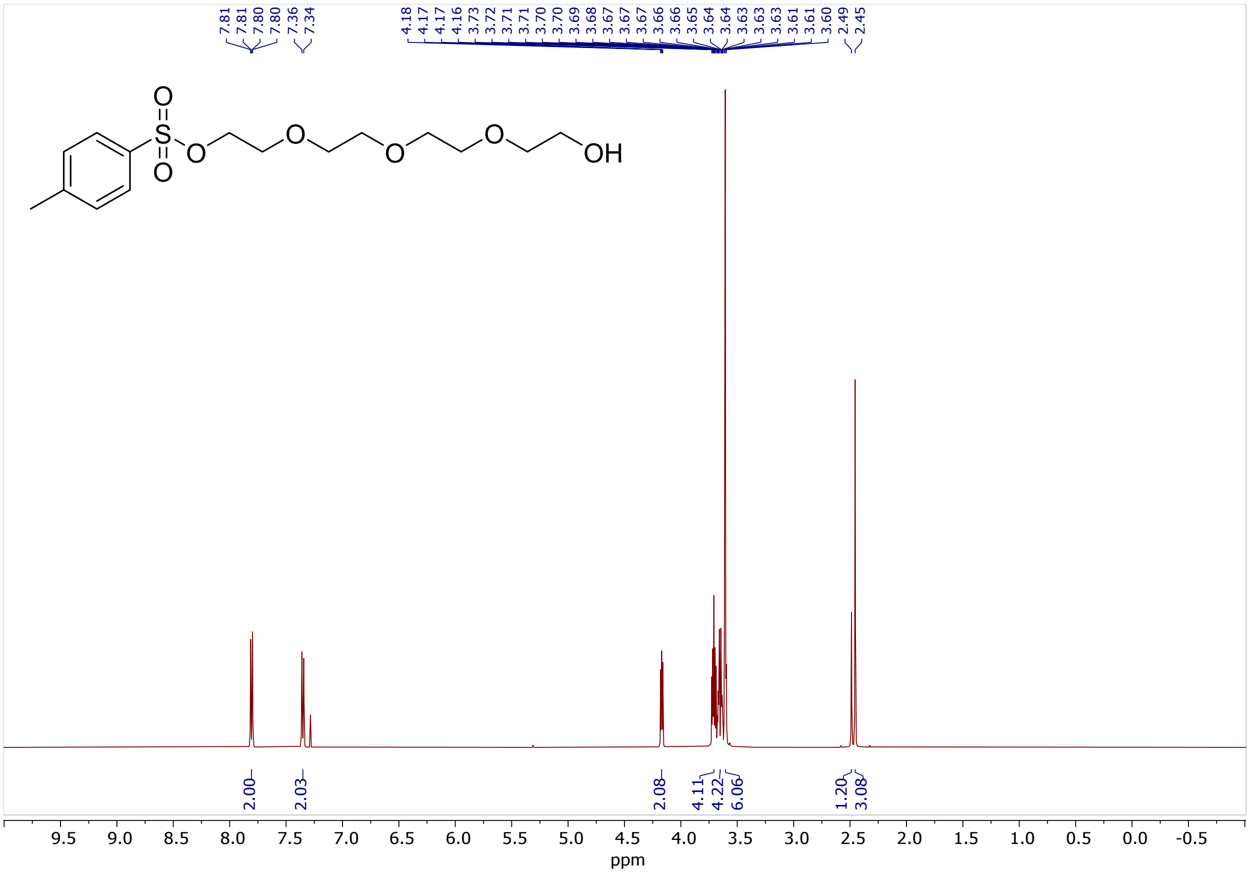

^{1}H-NMR (500 MHz, $CDCl_3$) spectrum of **10**

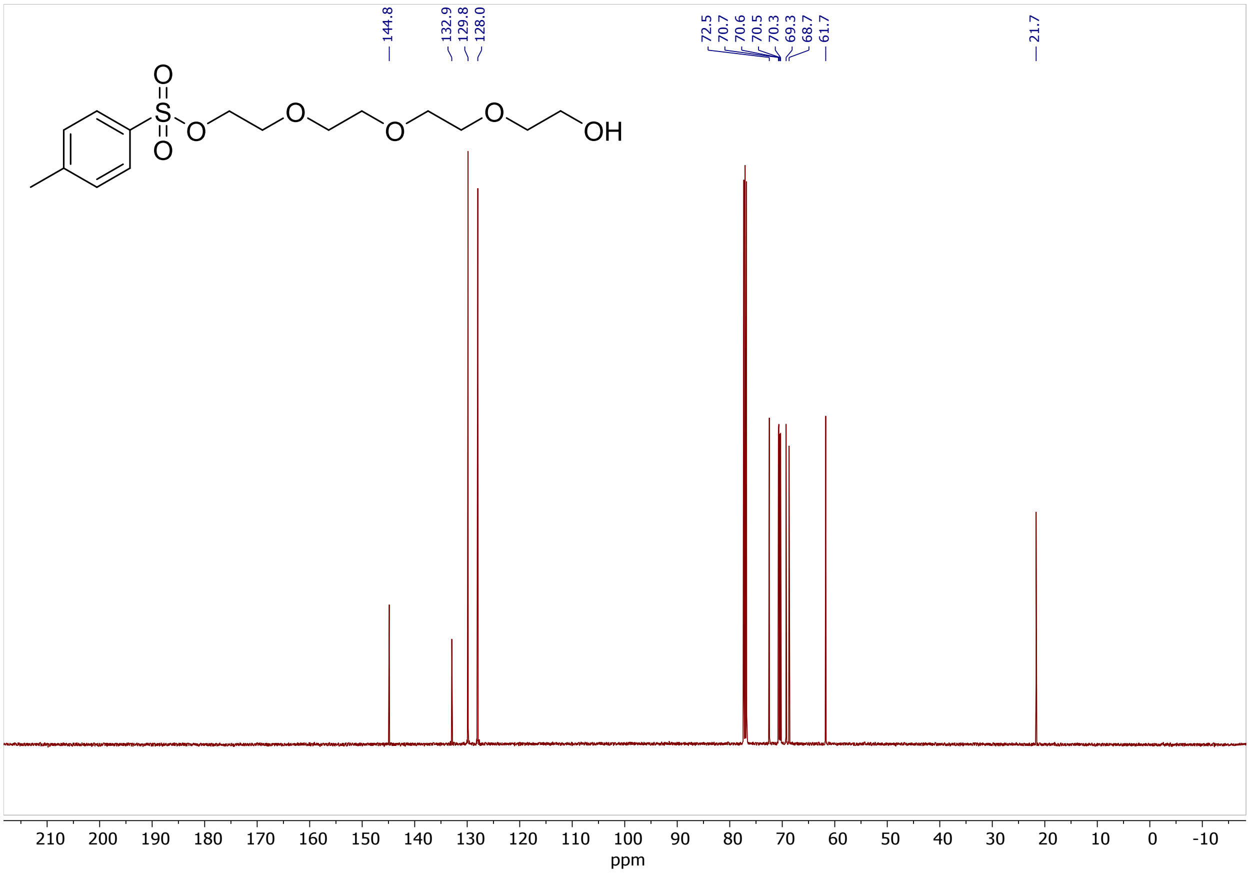

^{13}C{^{1}H}-NMR (126 MHz, $CDCl_3$) spectrum of **10**

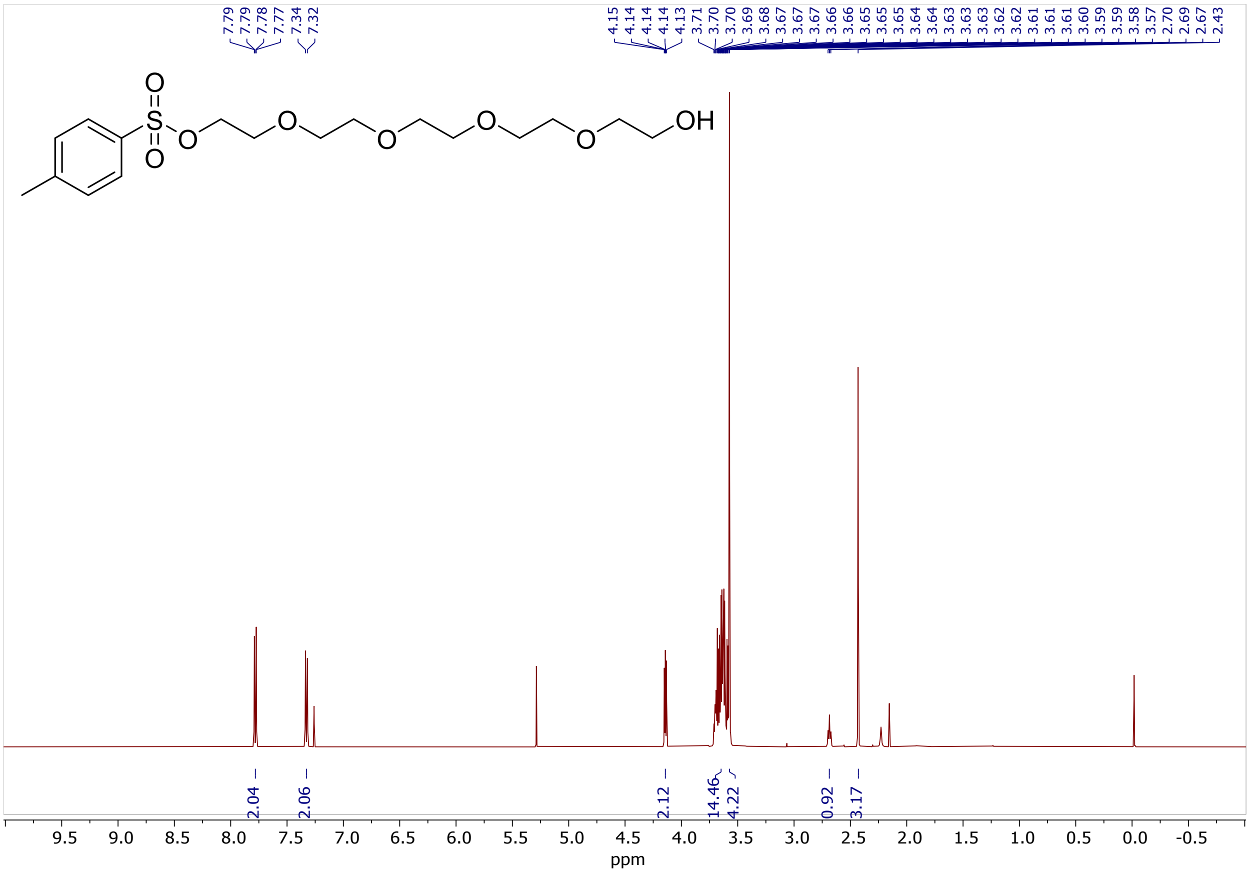

^{1}H-NMR (500 MHz, $CDCl_3$) spectrum of **11** (trace CH_2Cl_2, acetone)

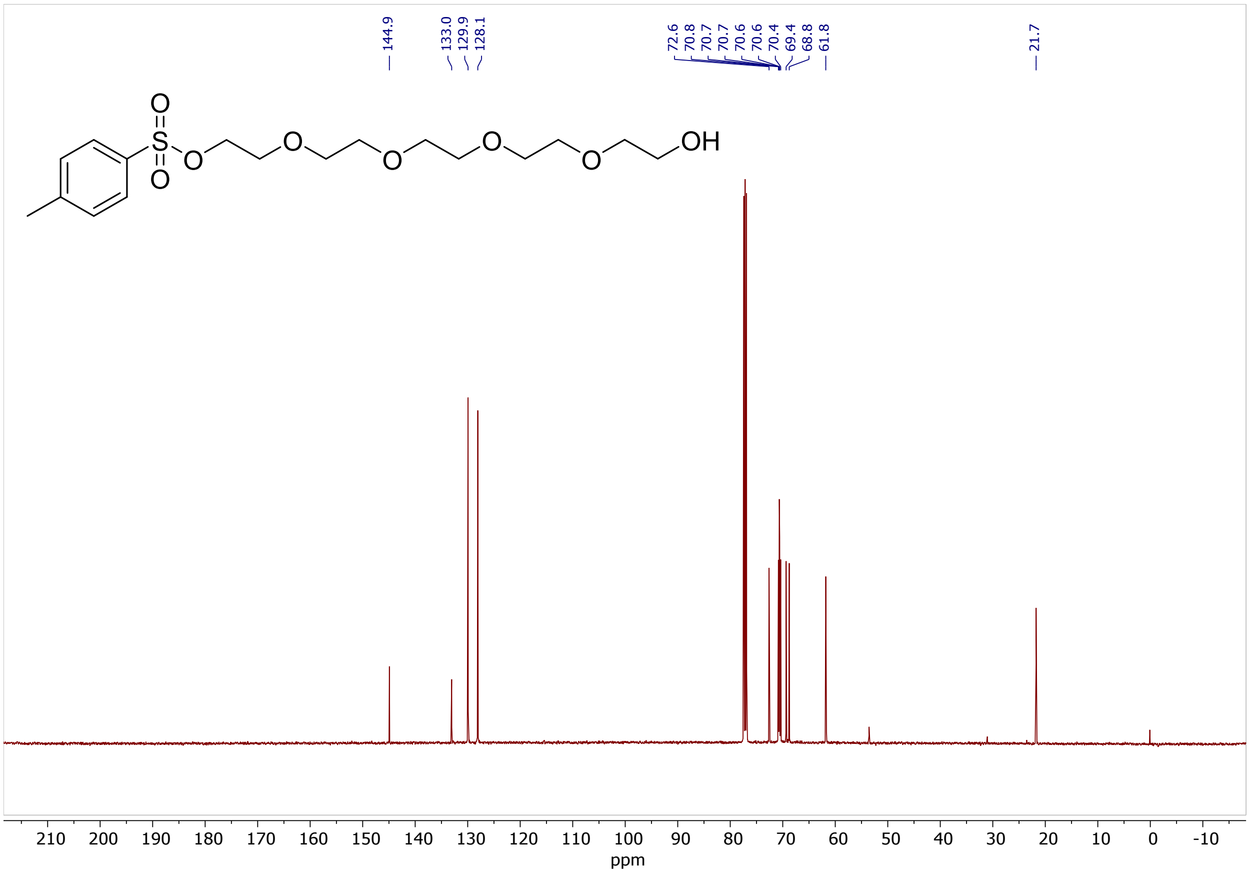

$^{13}C\{^{1}H\}$-NMR (126 MHz, $CDCl_3$) spectrum of **11** (trace CH_2Cl_2, acetone)

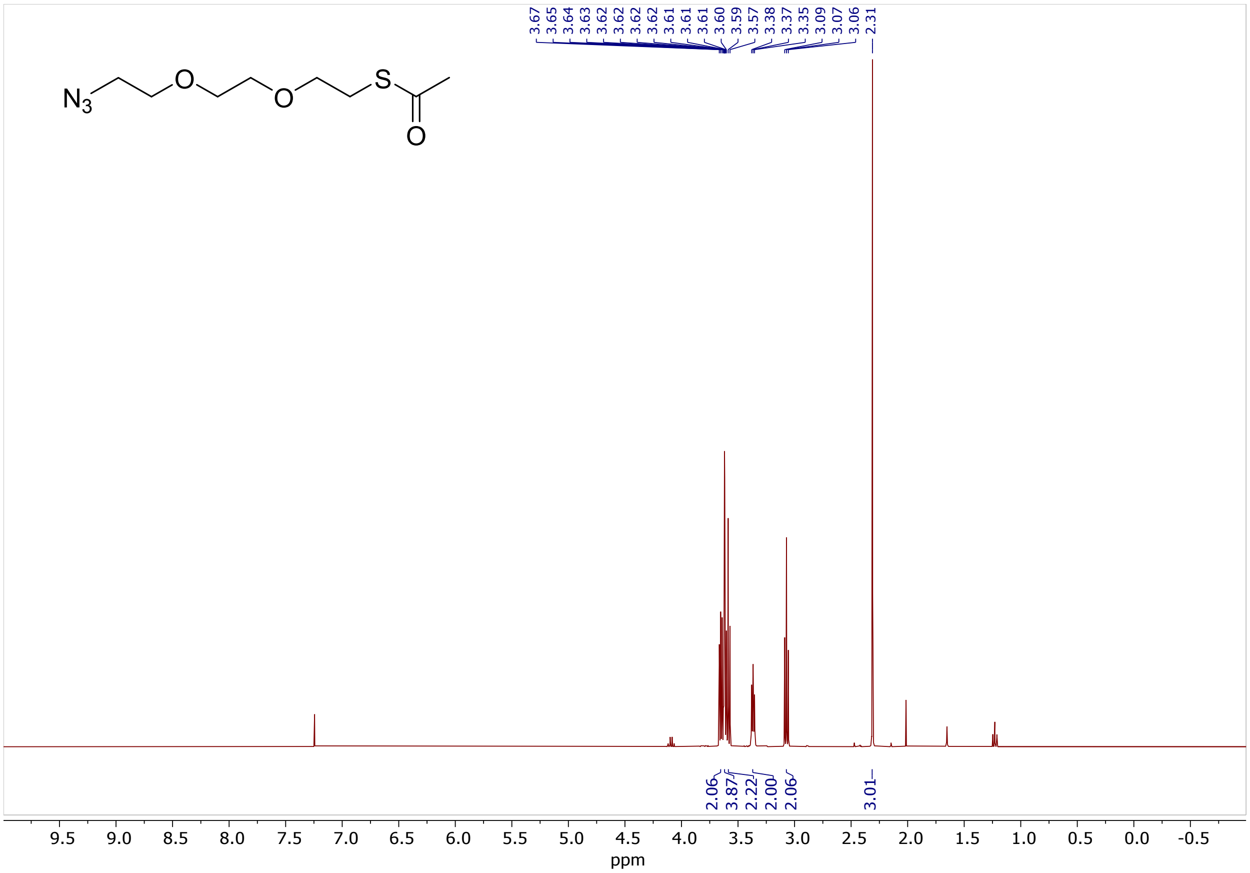

^{1}H-NMR (400 MHz, $CDCl_3$) spectrum of **21** (trace EtOAc)

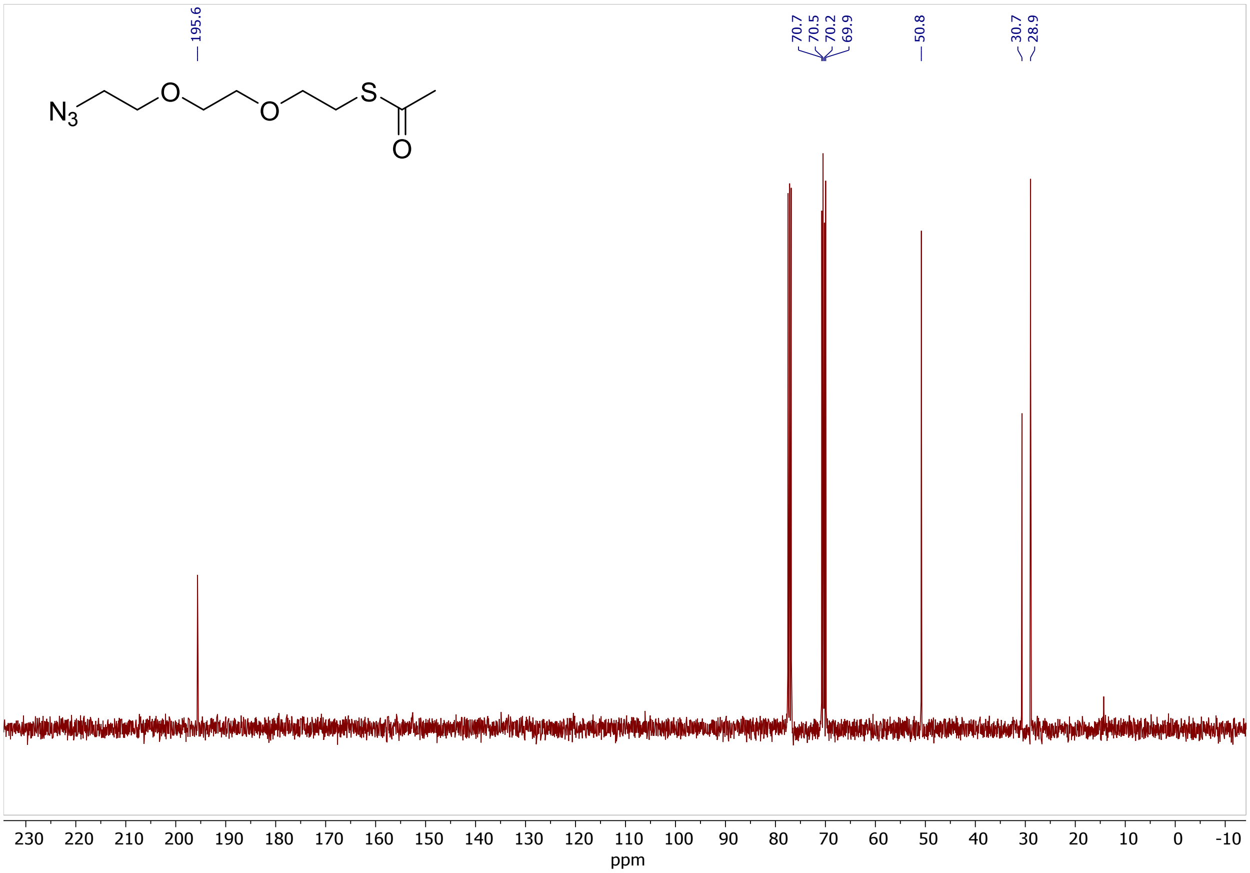

^{13}C{^{1}H}-NMR (101 MHz, $CDCl_3$) spectrum of **21** (trace EtOAc)

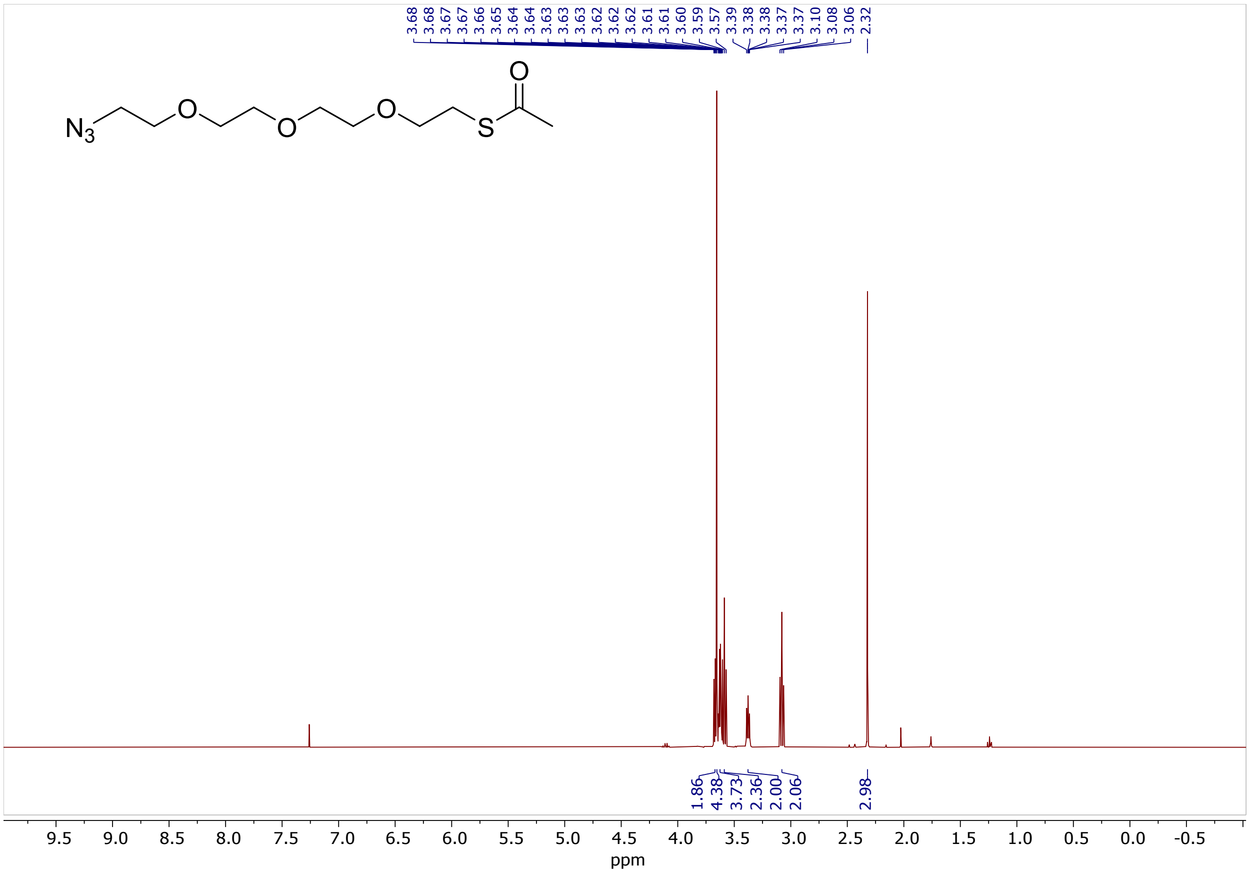

^{1}H-NMR (400 MHz, $CDCl_3$) spectrum of **22**

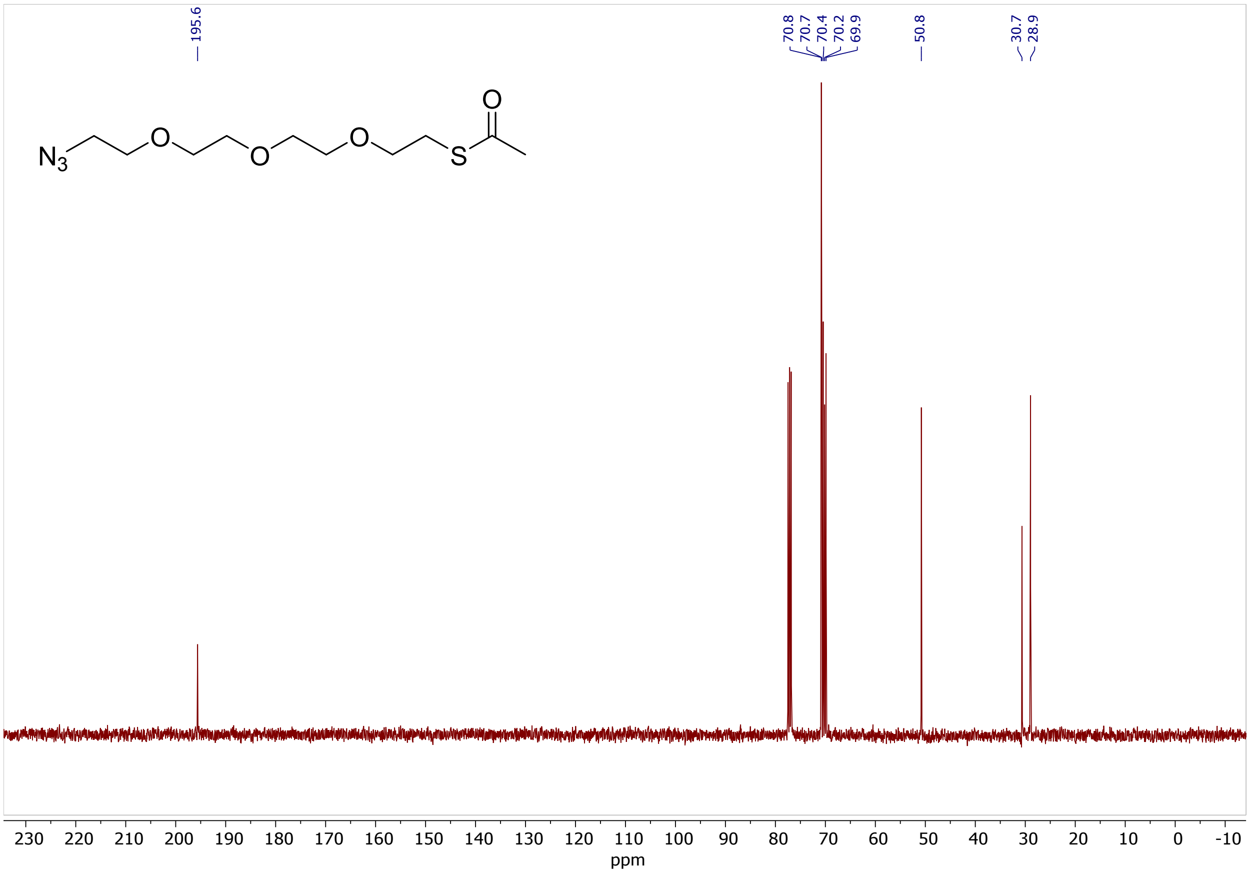

^{13}C{^{1}H}-NMR (101 MHz, $CDCl_3$) spectrum of **22**

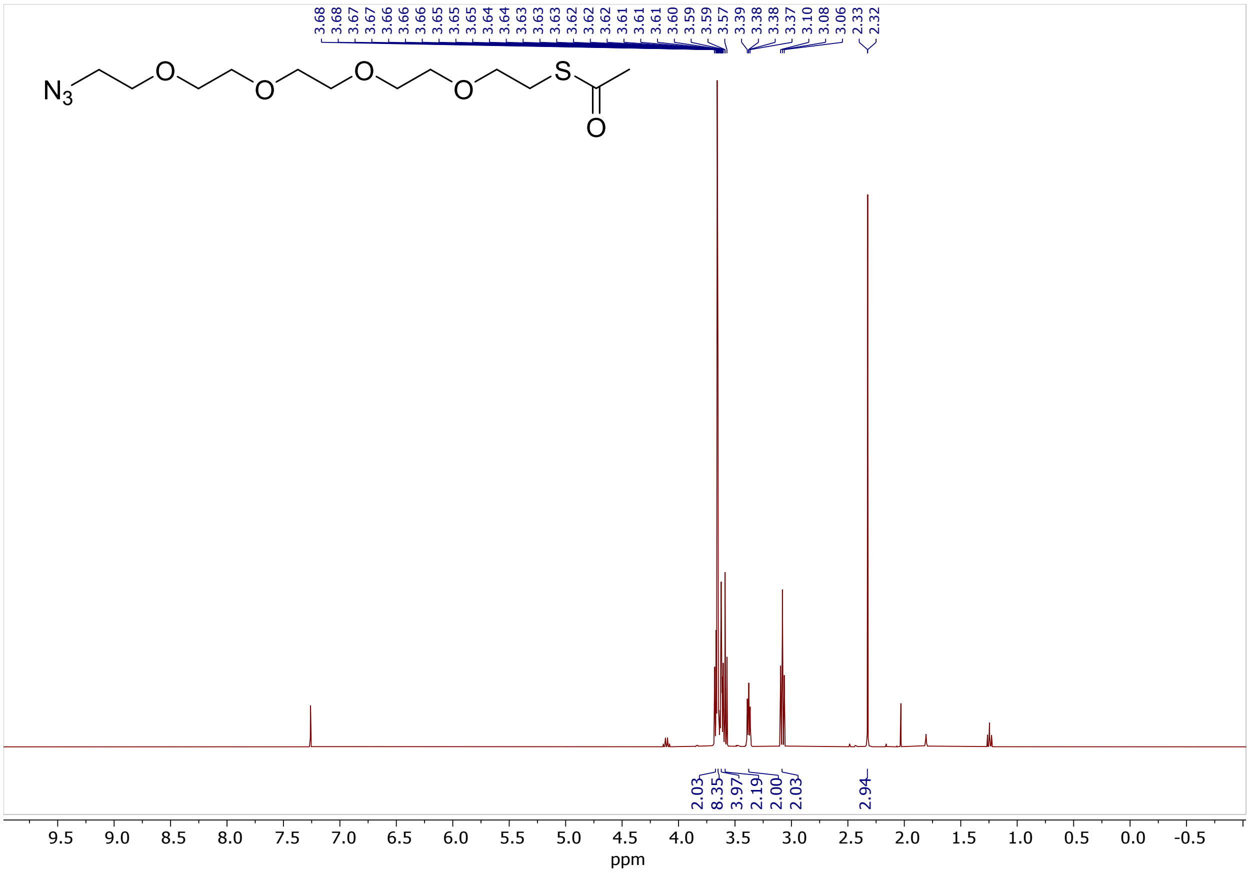

^{1}H-NMR (400 MHz, $CDCl_3$) spectrum of **23** (trace EtOAc)

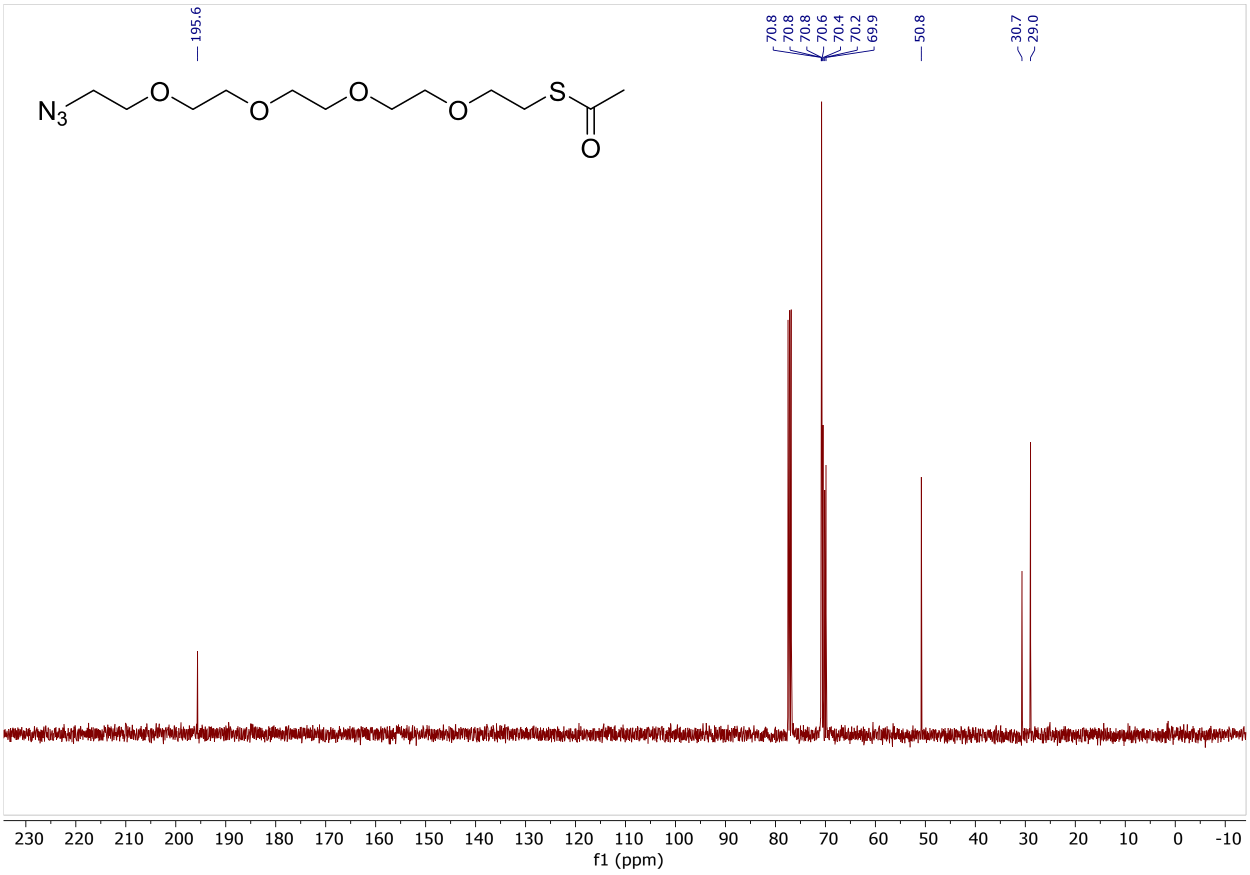

^{13}C{^{1}H}-NMR (101 MHz, $CDCl_3$) spectrum of **23** (trace EtOAc)

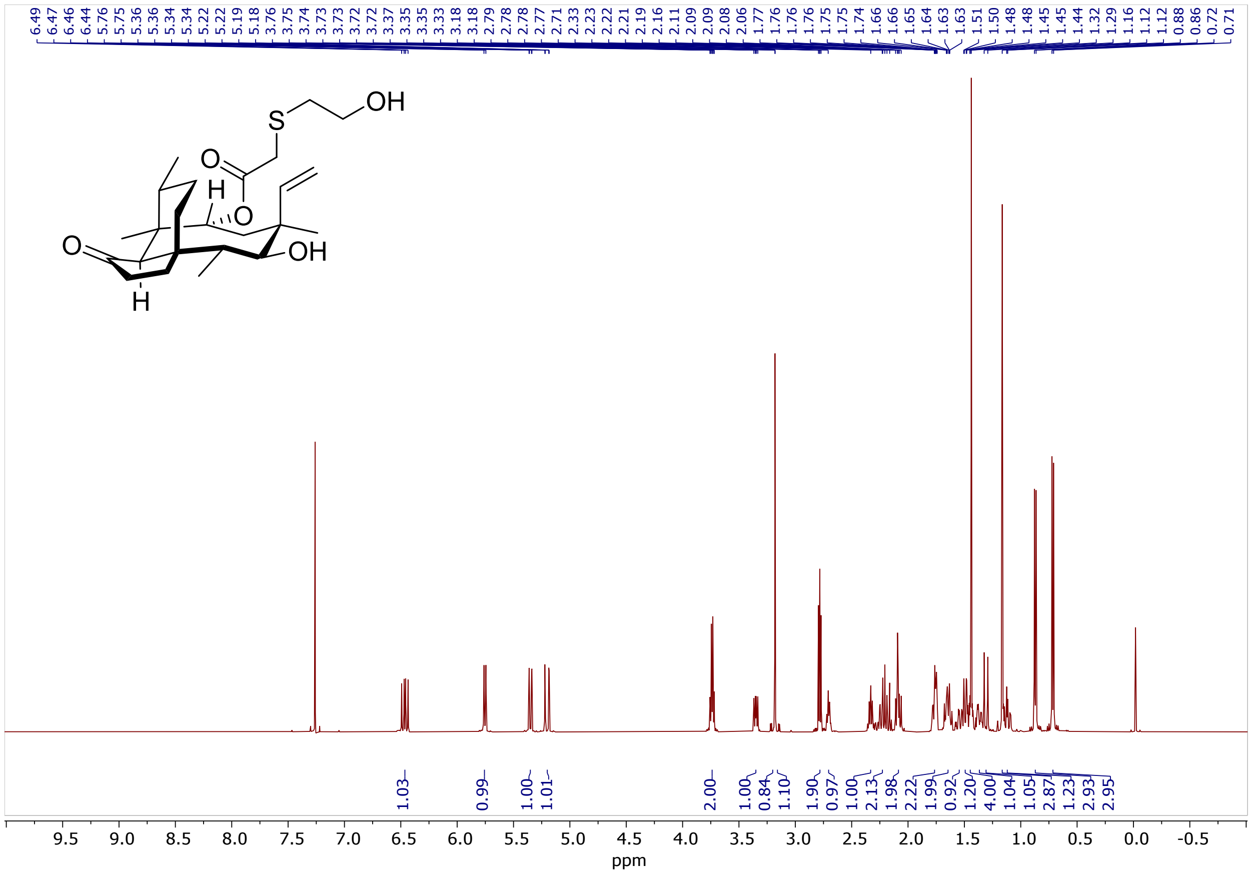

^{1}H-NMR (500 MHz, $CDCl_3$) spectrum of **25**

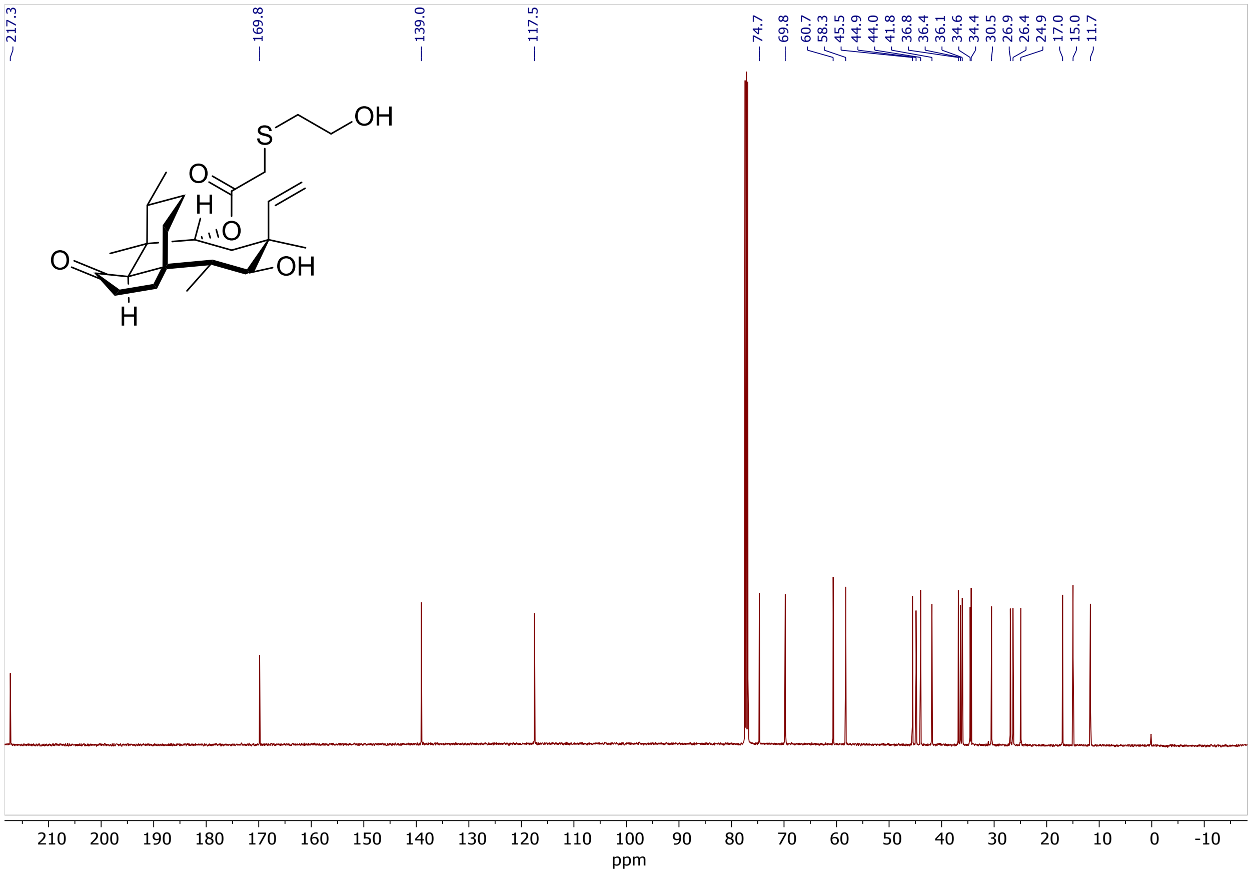

^{13}C{^{1}H}-NMR (126 MHz, $CDCl_3$) spectrum of **25**

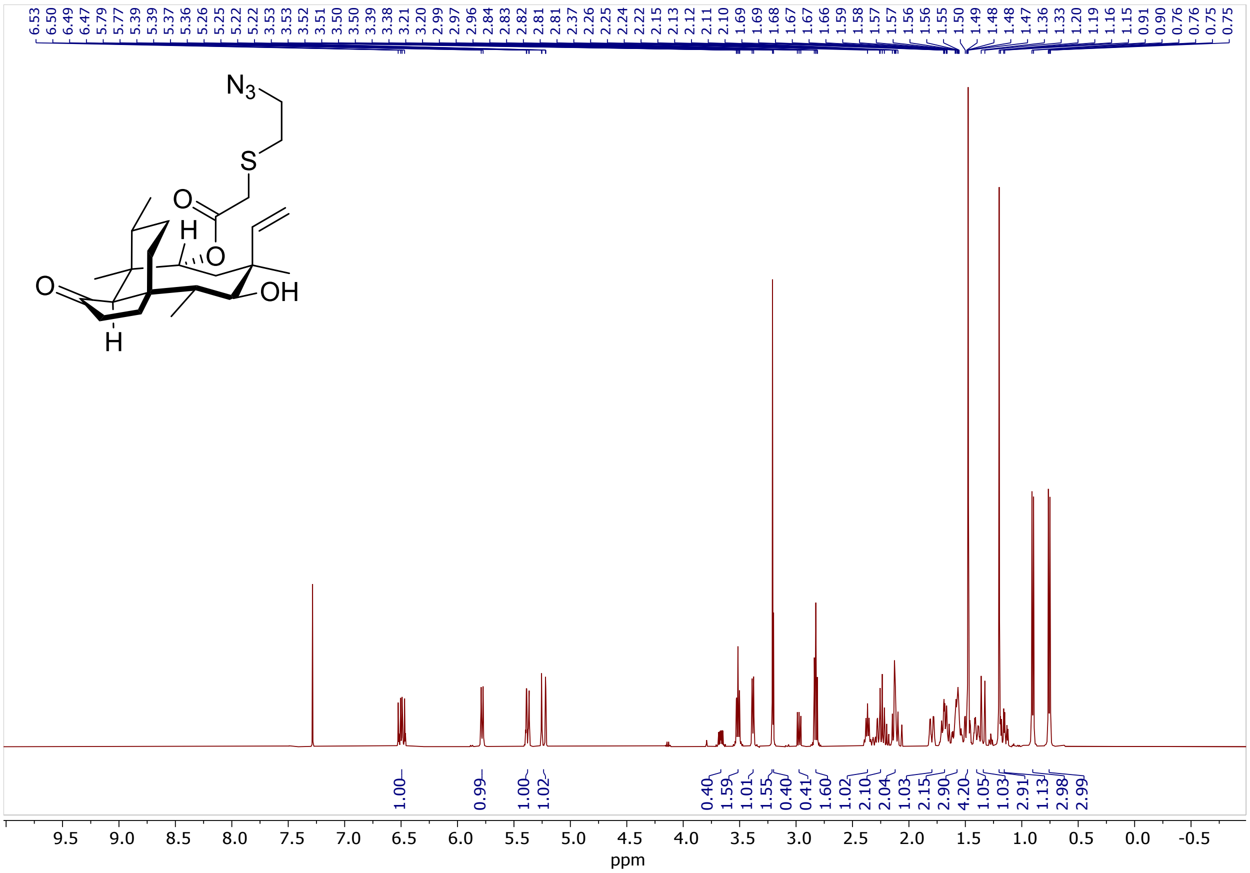

^{1}H-NMR (500 MHz, $CDCl_3$) spectrum of **28**

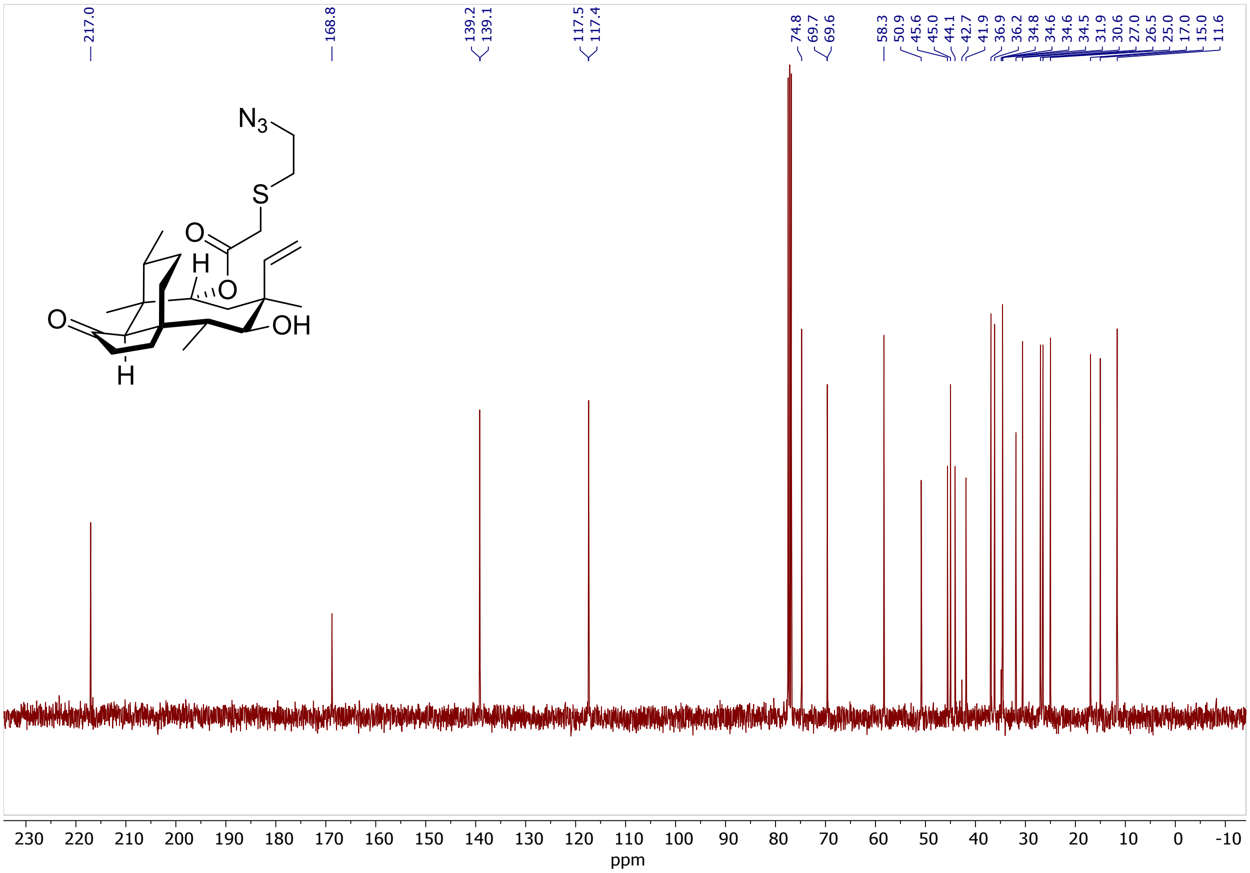

$^{13}C\{^{1}H\}$-NMR (101 MHz, $CDCl_3$) spectrum of **28**

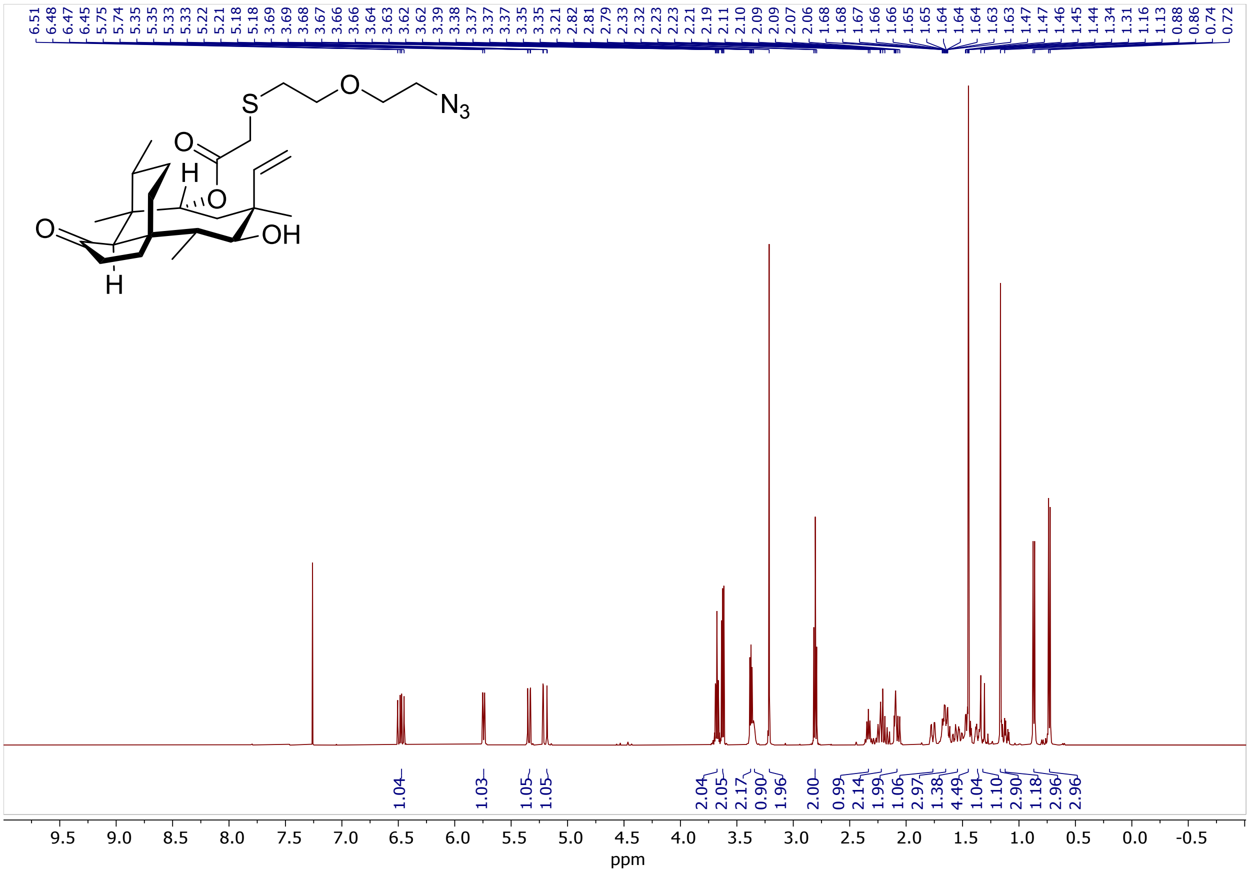

^{1}H-NMR (500 MHz, $CDCl_3$) spectrum of **29**

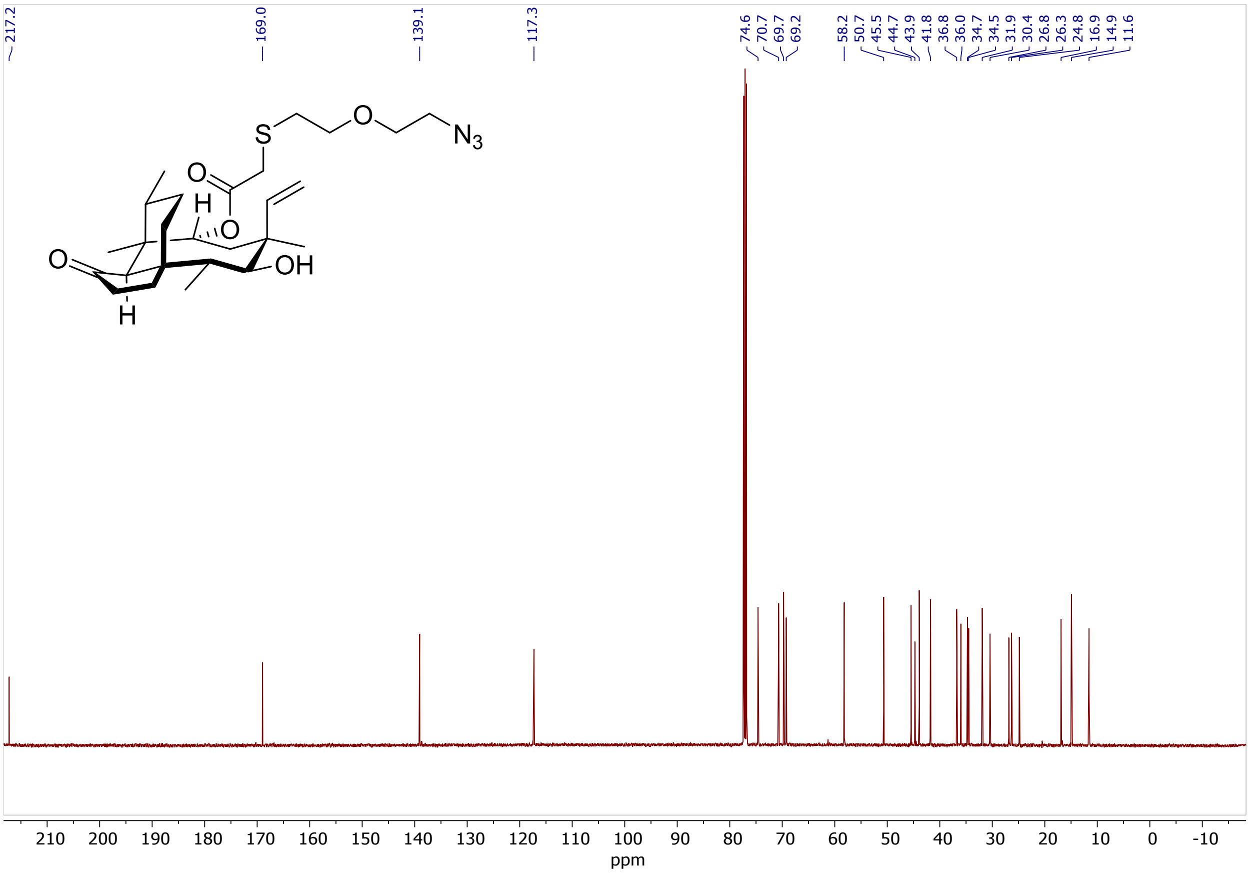

^{13}C{^{1}H}-NMR (126 MHz, $CDCl_3$) spectrum of **29**

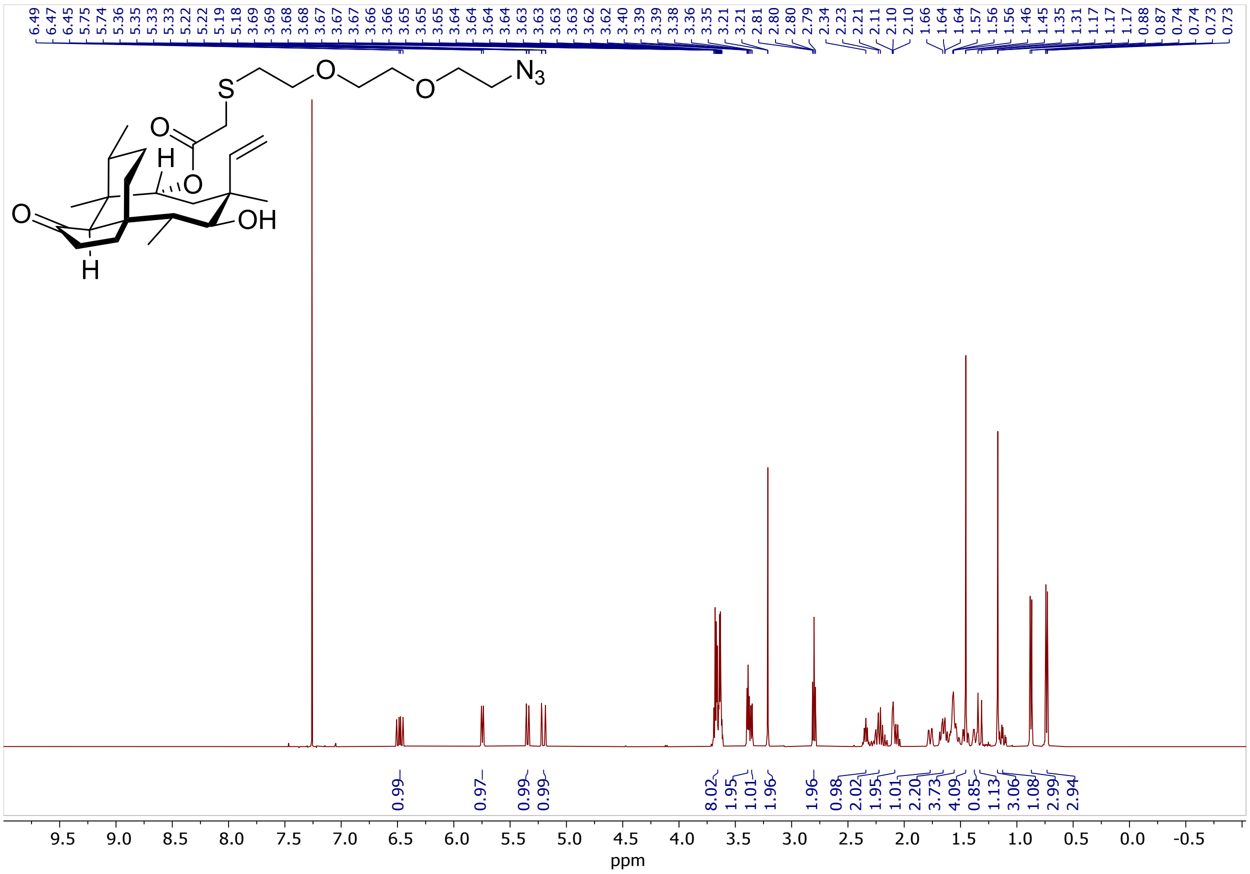

^{1}H-NMR (500 MHz, $CDCl_3$) spectrum of **30**

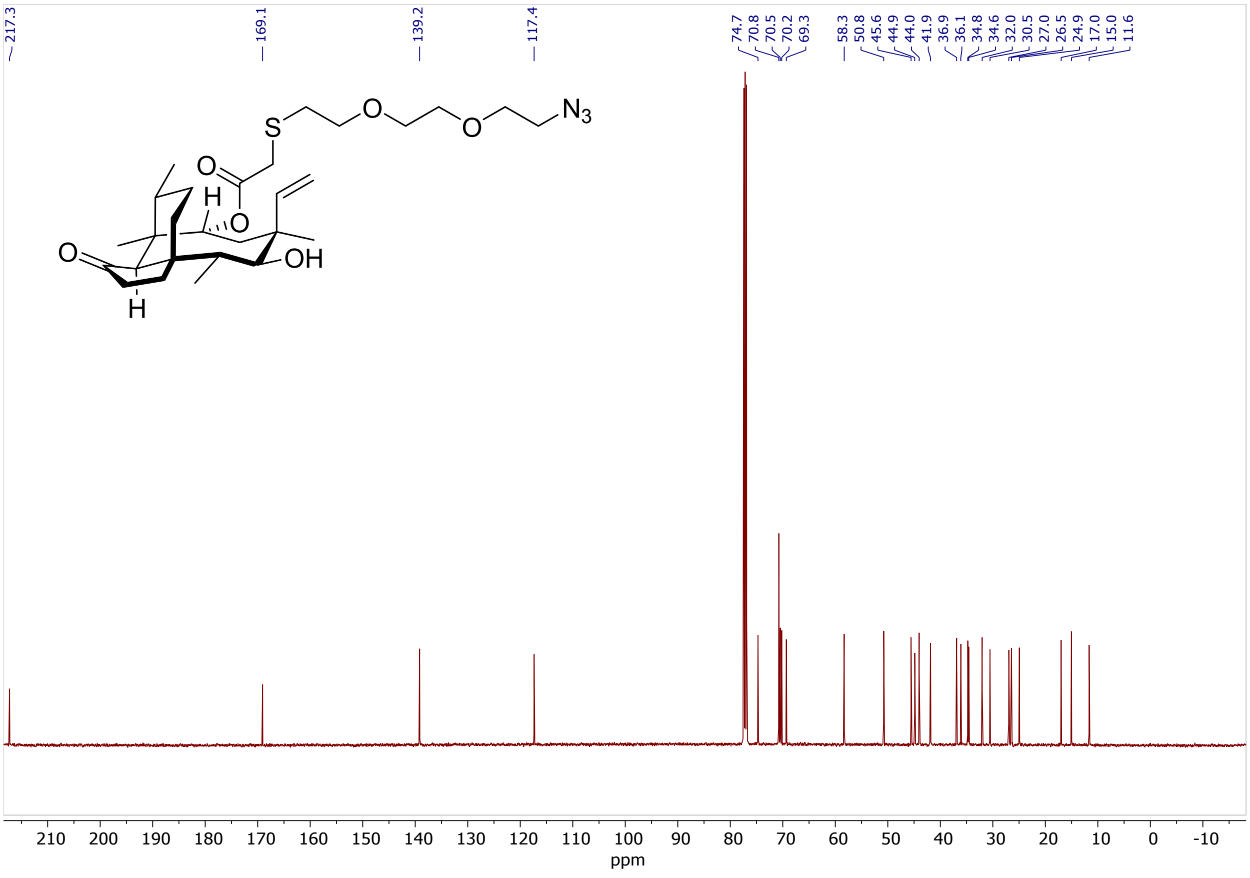

^{13}C{^{1}H}-NMR (126 MHz, $CDCl_3$) spectrum of **30**

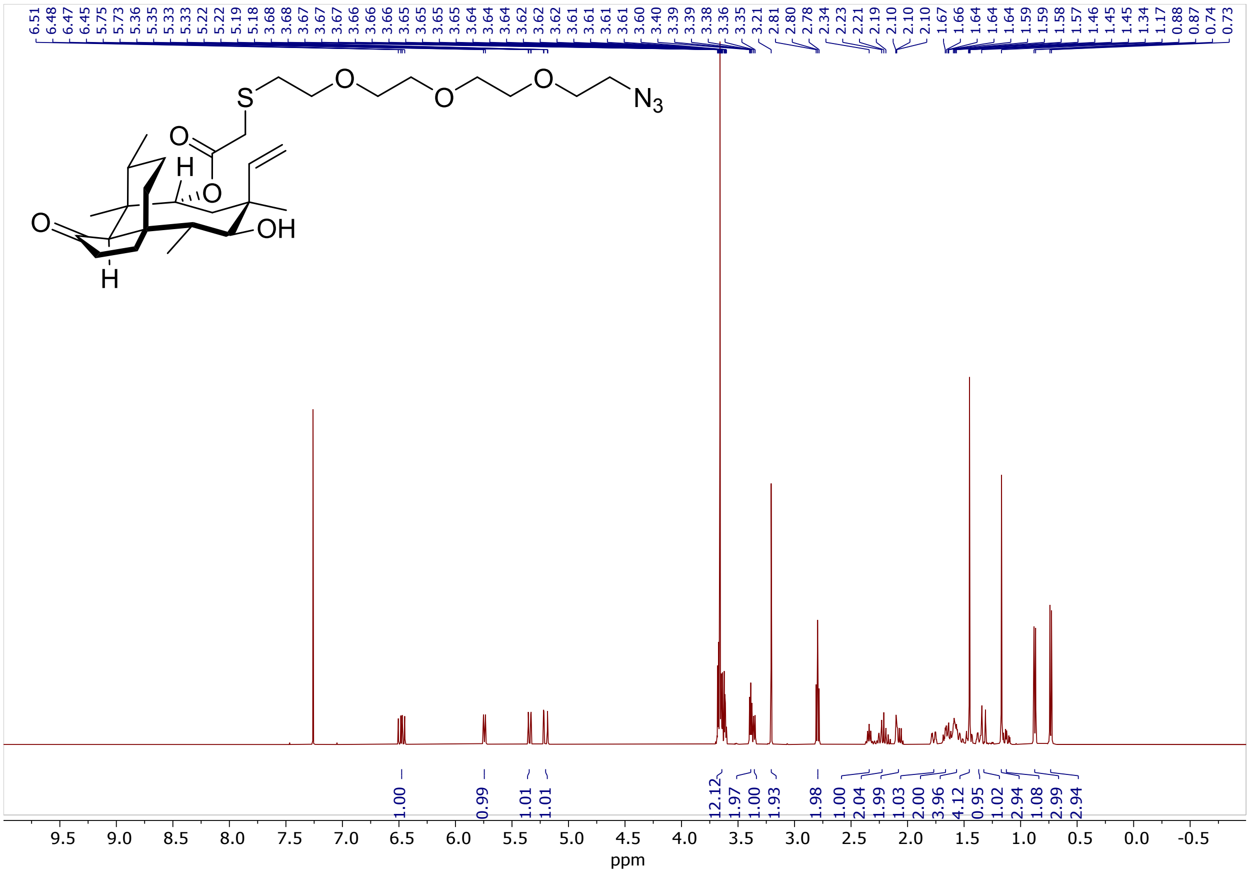

^{1}H-NMR (500 MHz, $CDCl_3$) spectrum of **31**

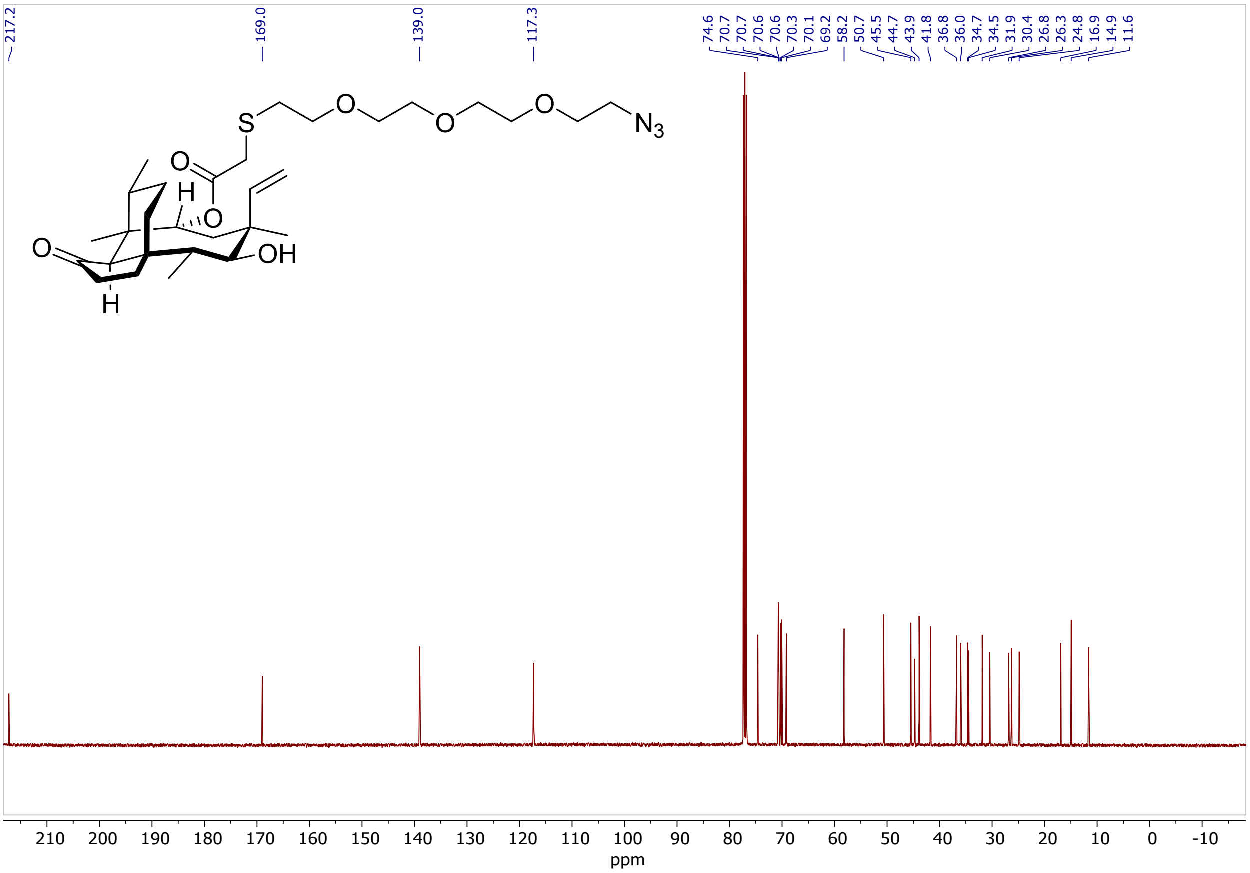

^{13}C{^{1}H}-NMR (126 MHz, $CDCl_3$) spectrum of **31**

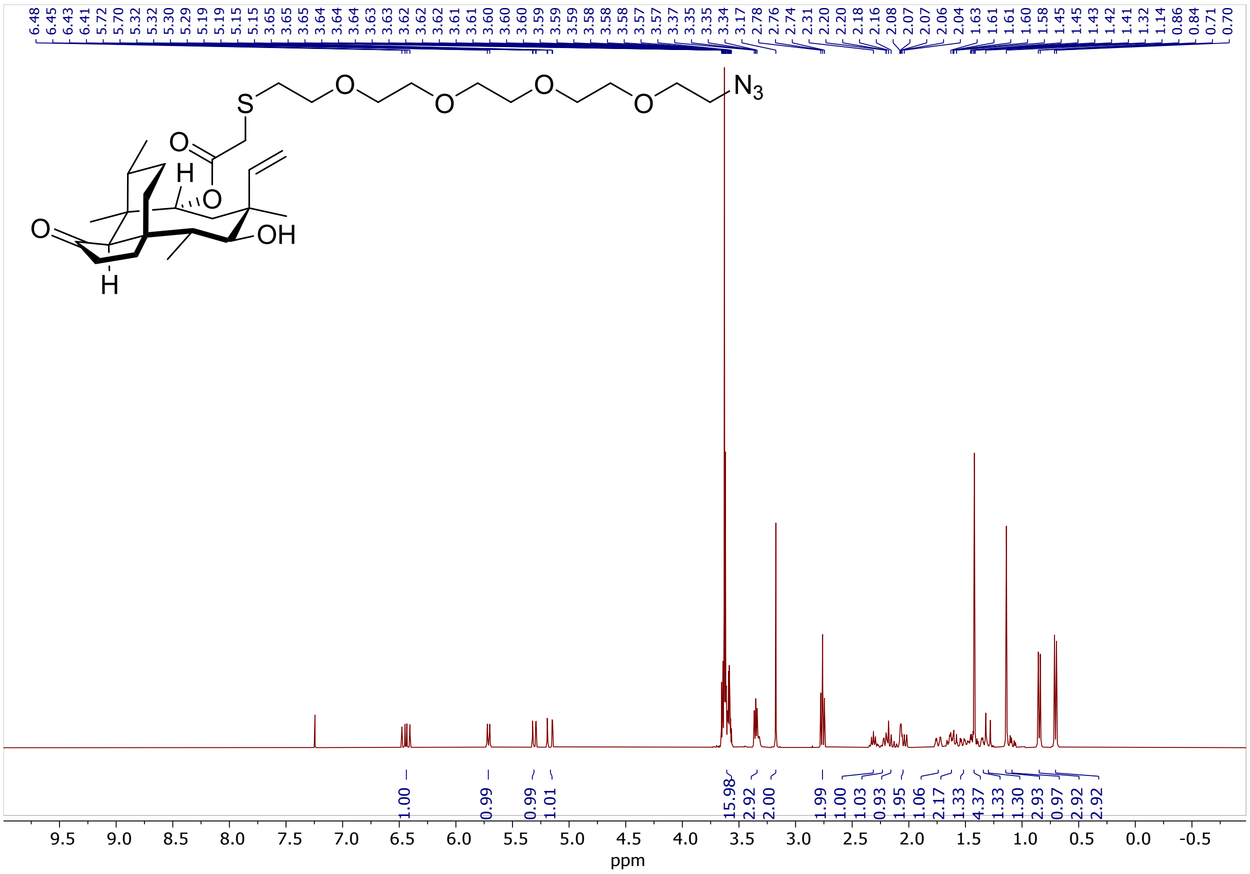

^{1}H-NMR (500 MHz, $CDCl_3$) spectrum of **32**

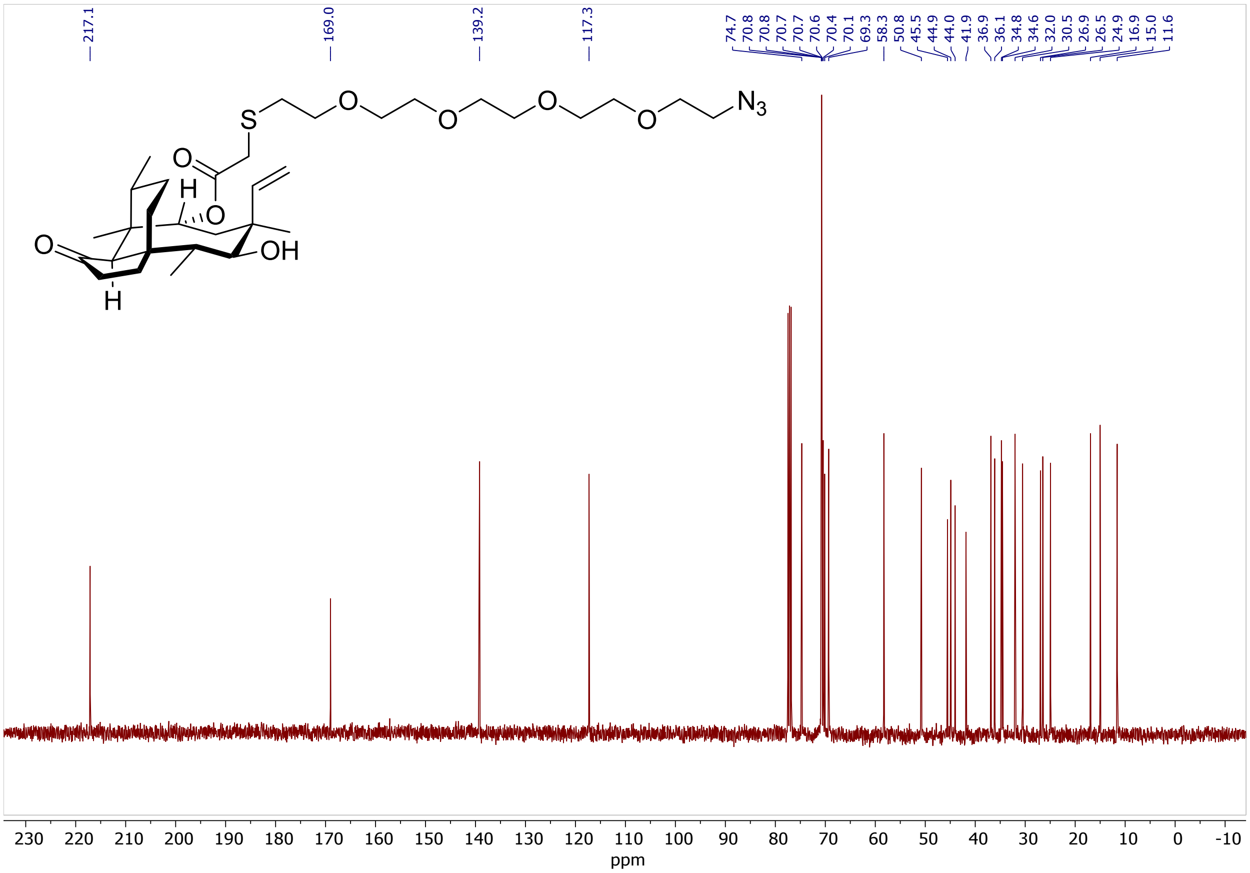

^{13}C{^{1}H}-NMR (126 MHz, $CDCl_3$) spectrum of **32**

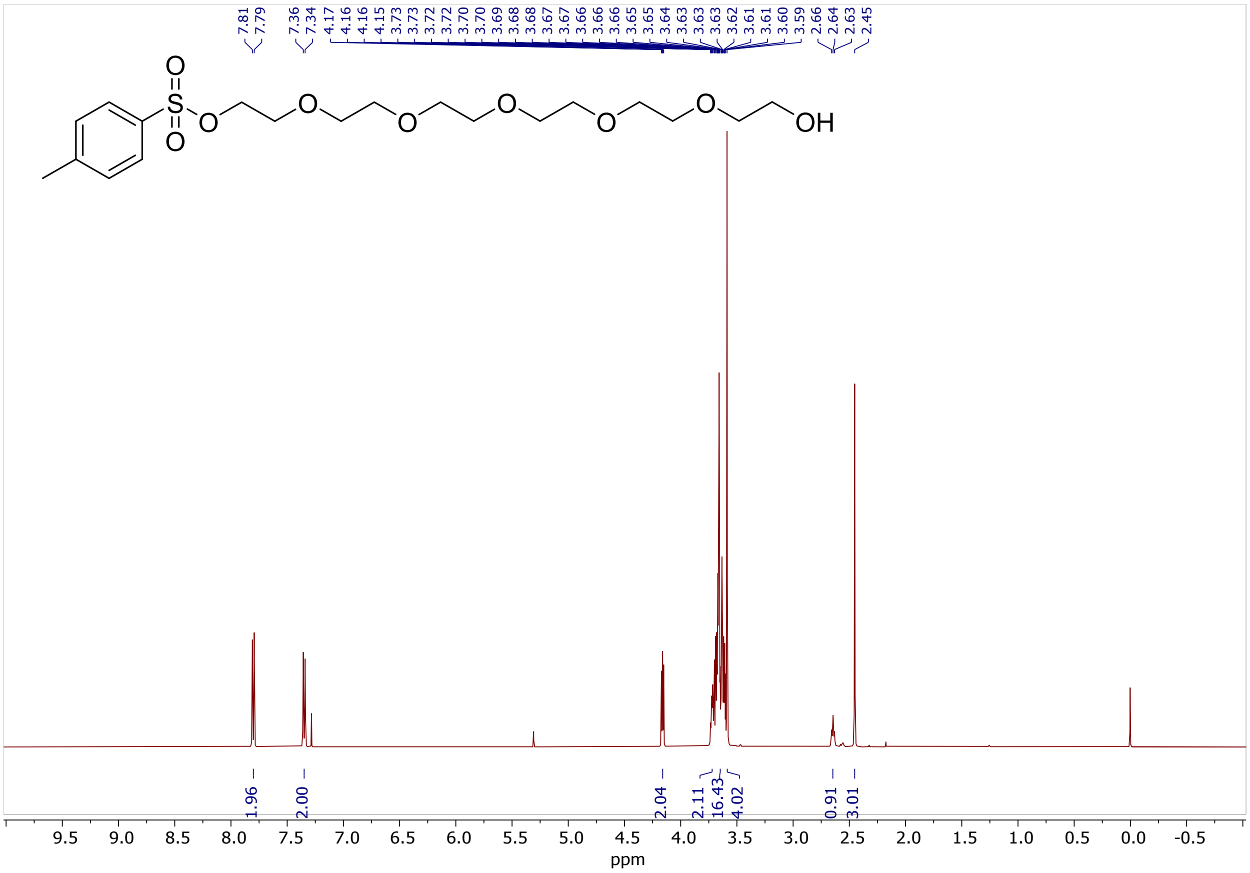

^{1}H-NMR (500 MHz, $CDCl_3$) spectrum of **35**

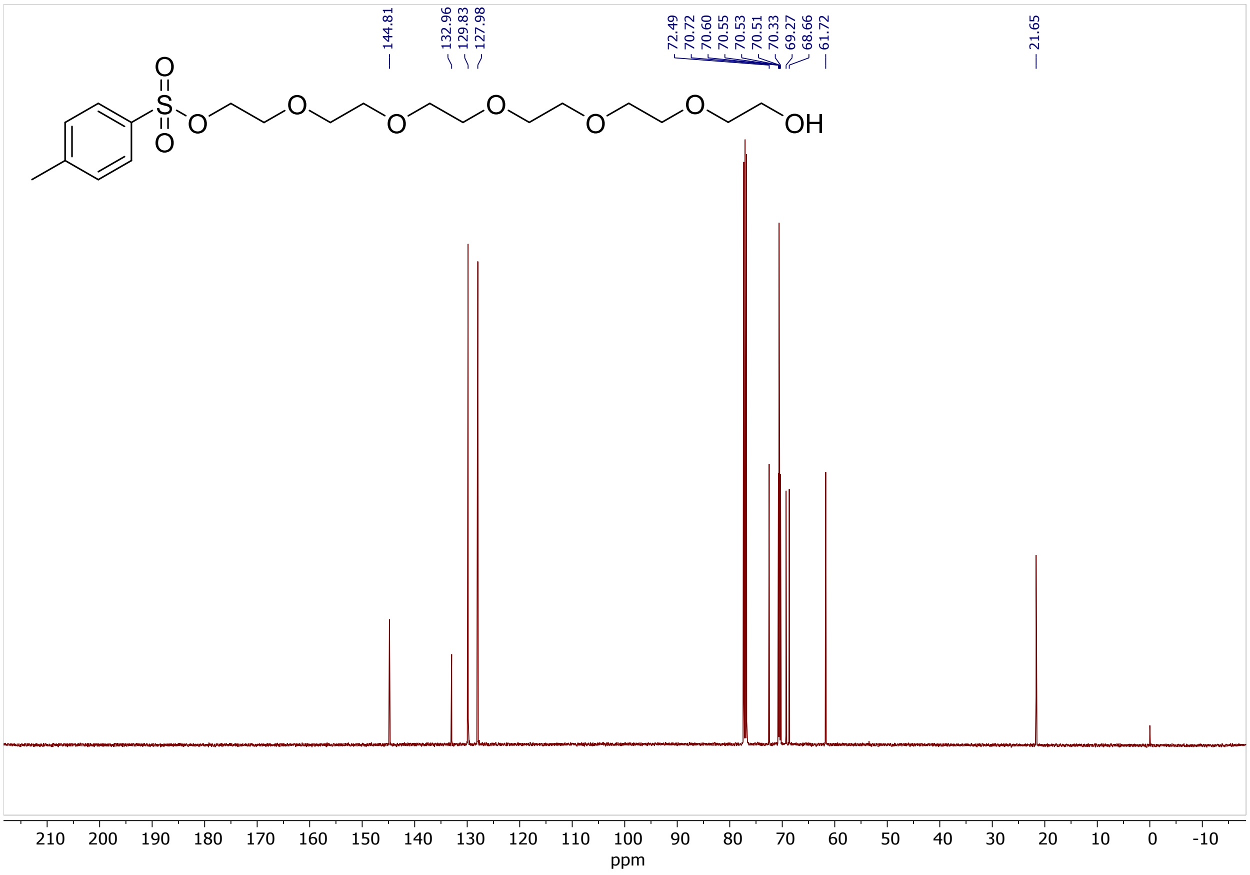

^{13}C{^{1}H}-NMR (126 MHz, $CDCl_3$) spectrum of **35**

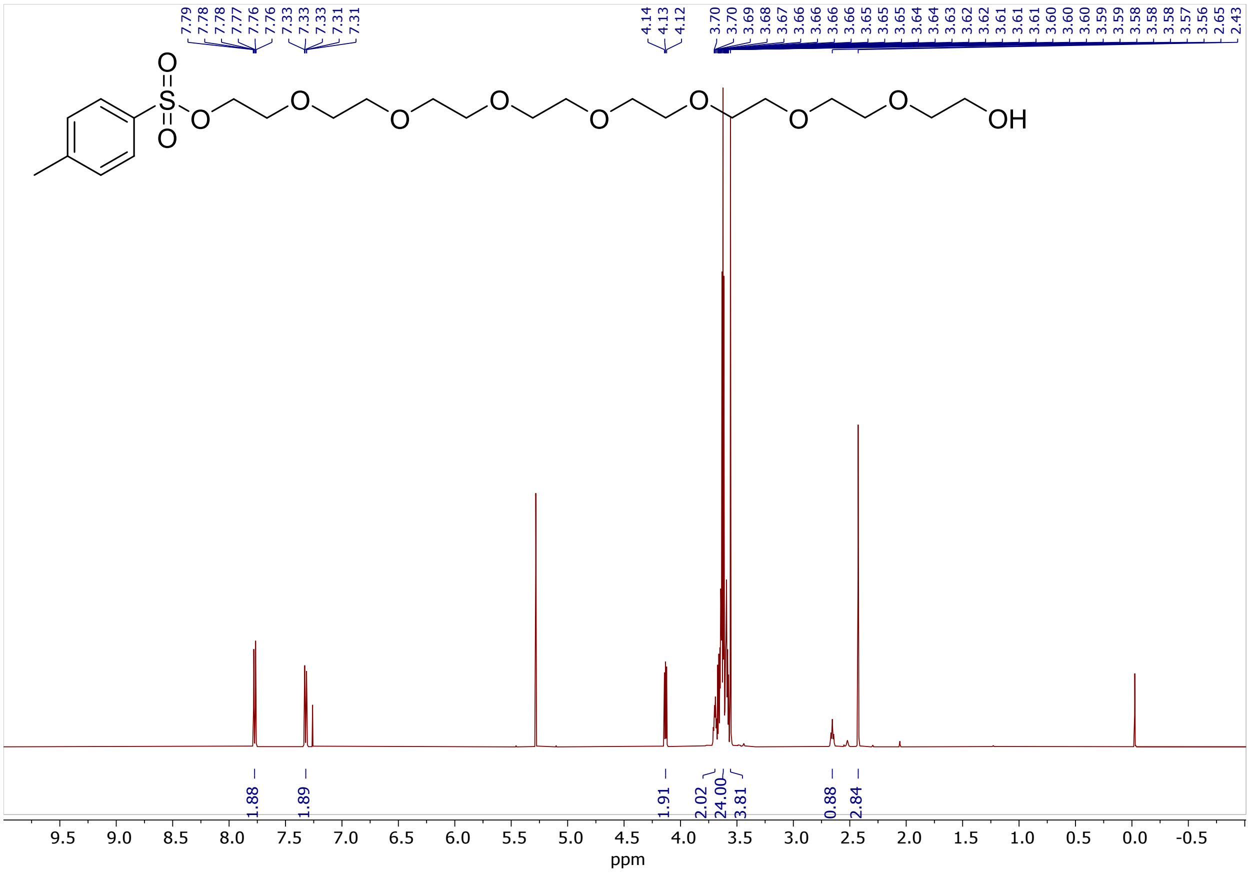

^{1}H-NMR (500 MHz, $CDCl_3$) spectrum of **36** (trace CH_2Cl_2)

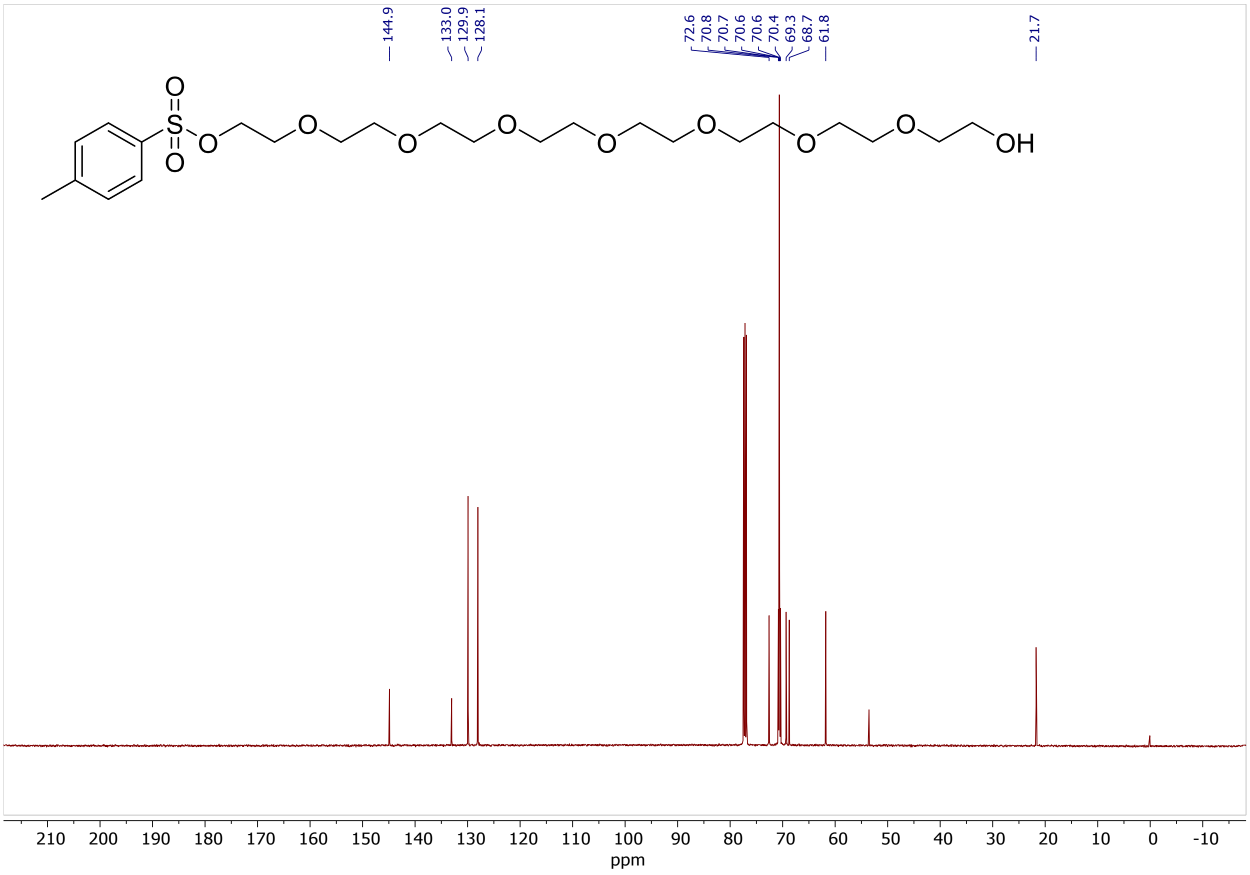

$^{13}C\{^{1}H\}$-NMR (126 MHz, $CDCl_3$) spectrum of **36** (trace CH_2Cl_2)

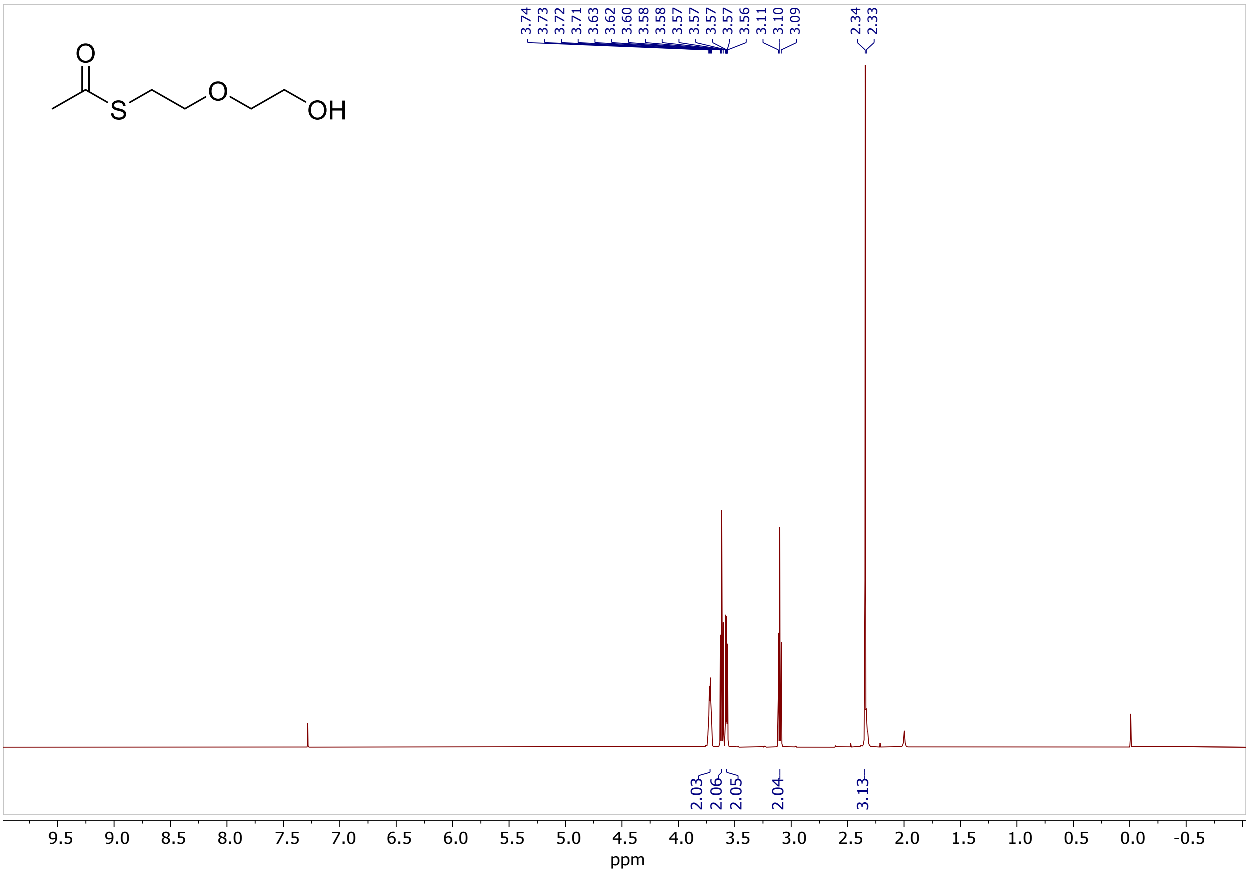

^{1}H-NMR (500 MHz, $CDCl_3$) spectrum of **37** (trace acetate)

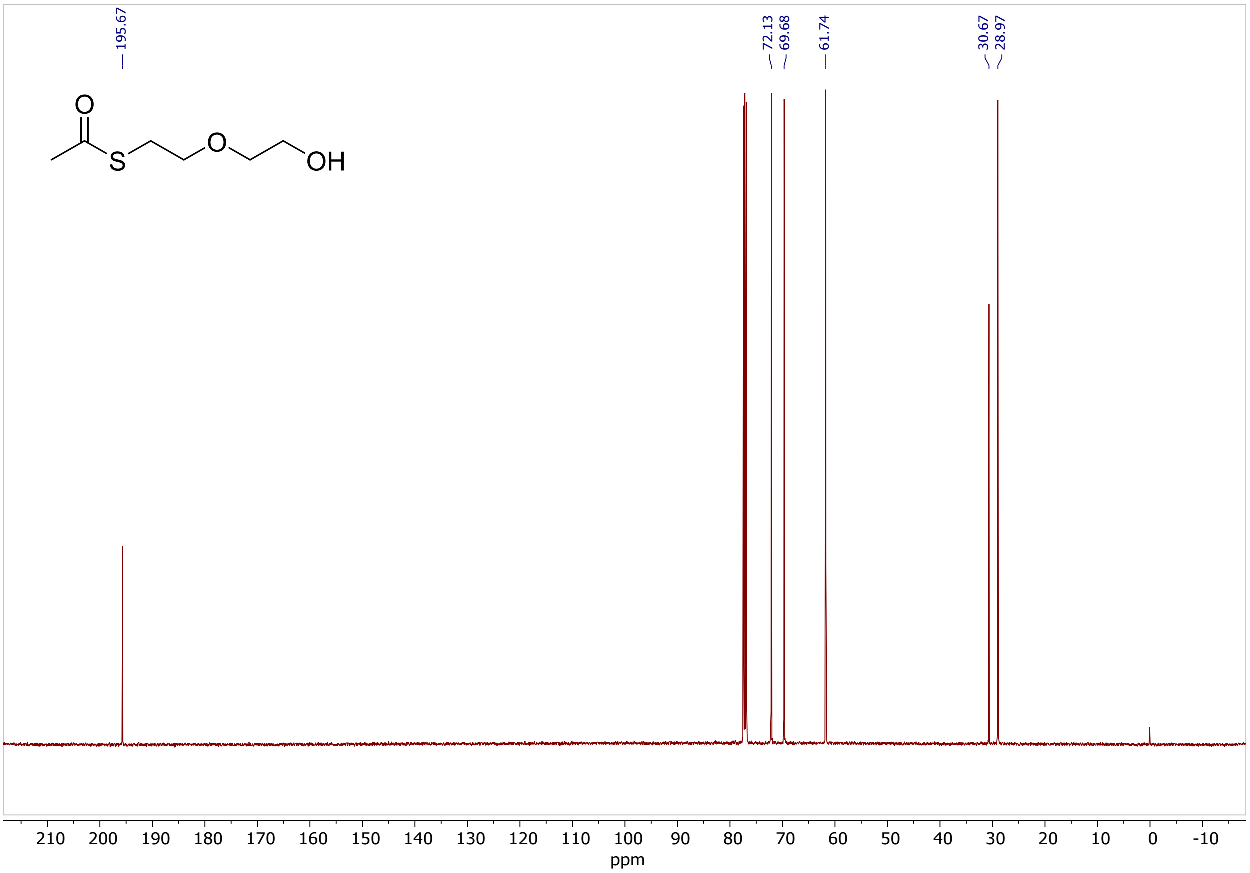

^{13}C{^{1}H}-NMR (126 MHz, $CDCl_3$) spectrum of **37** (trace acetate)

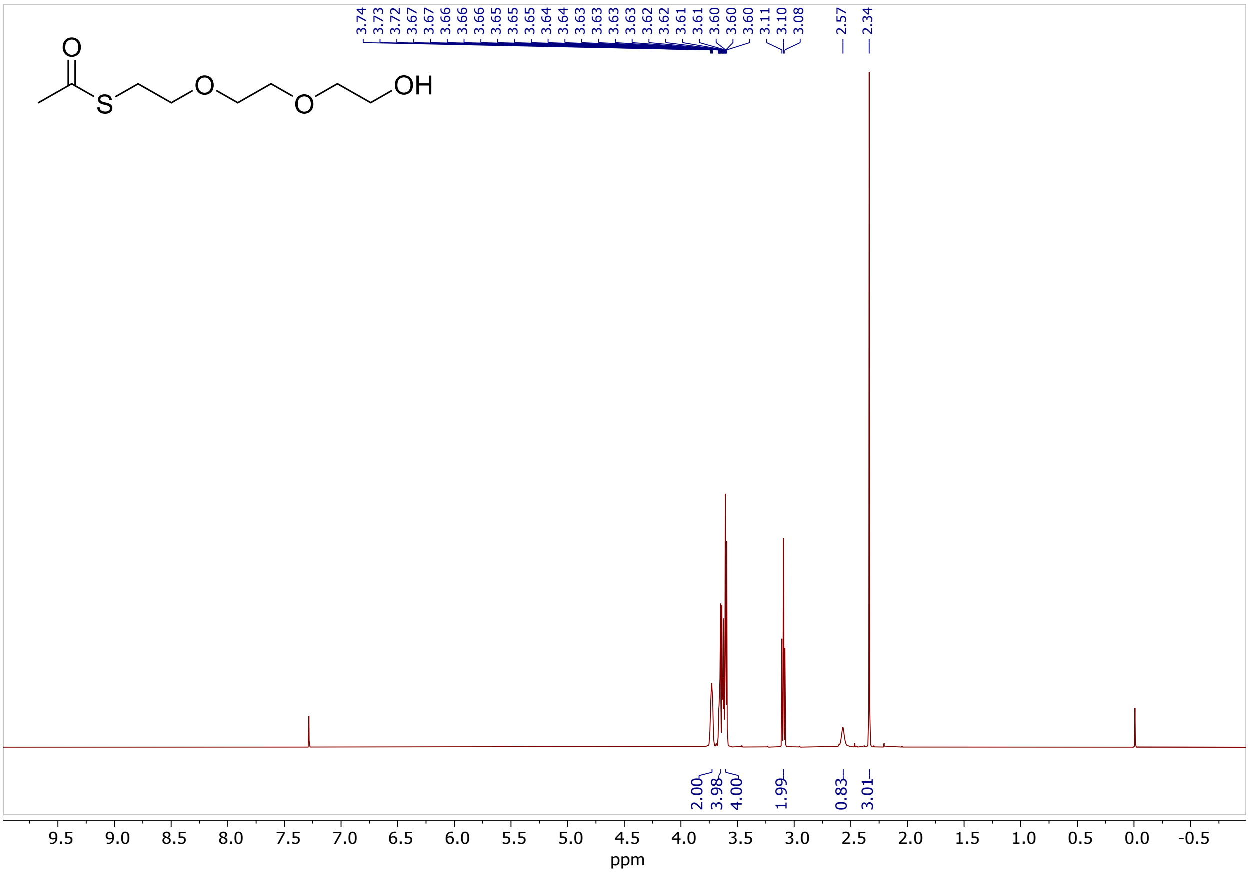

^{1}H-NMR (500 MHz, $CDCl_3$) spectrum of **38**

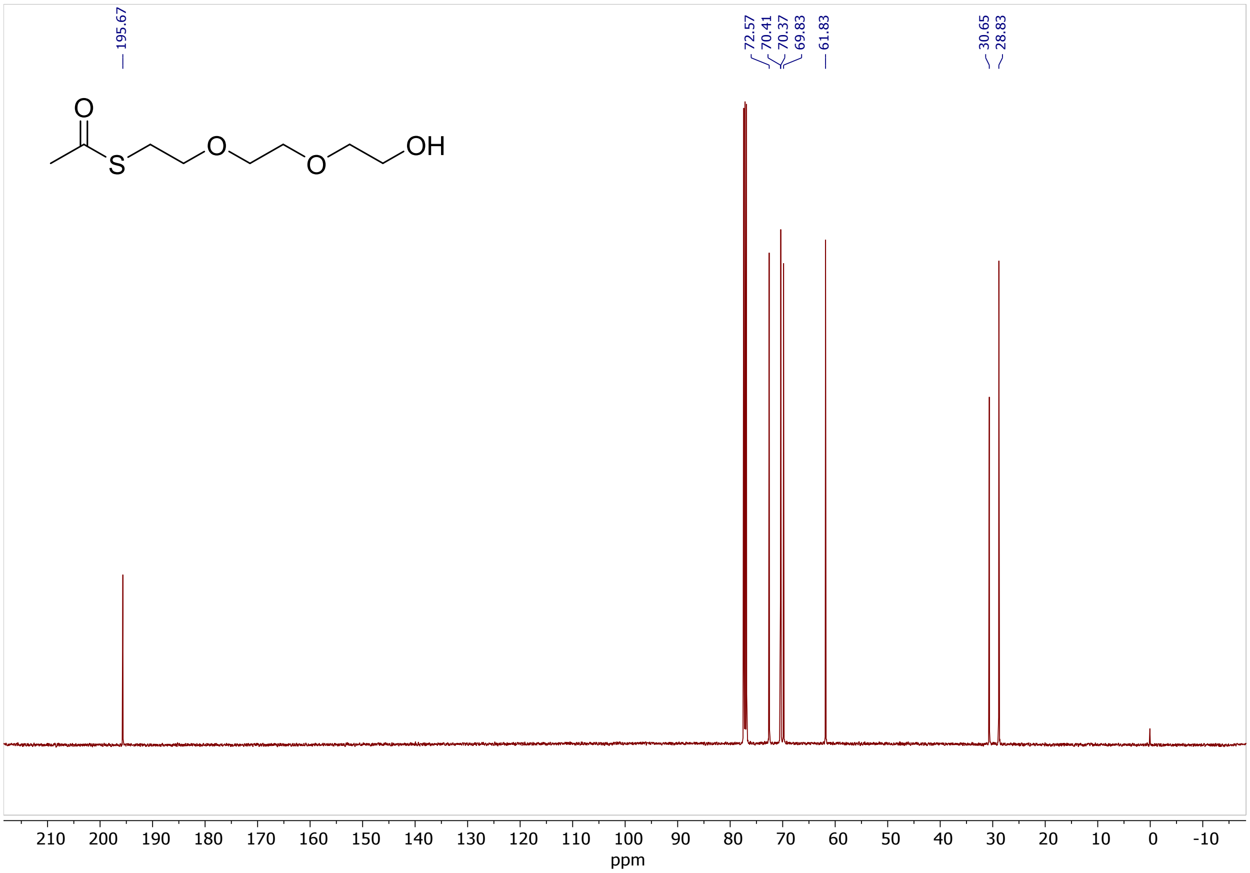

^{13}C{^{1}H}-NMR (126 MHz, $CDCl_3$) spectrum of **38**

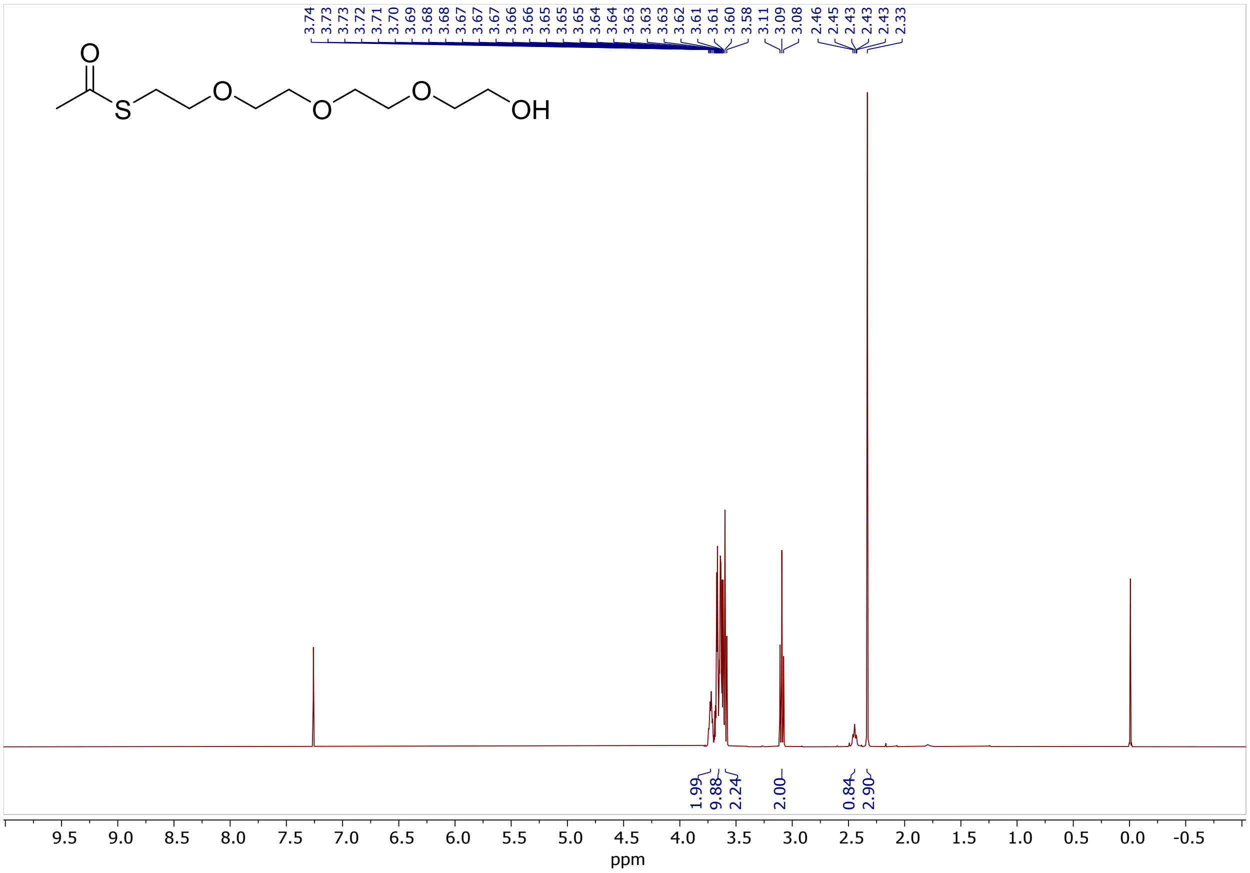

^{1}H-NMR (400 MHz, $CDCl_3$) spectrum of **39**

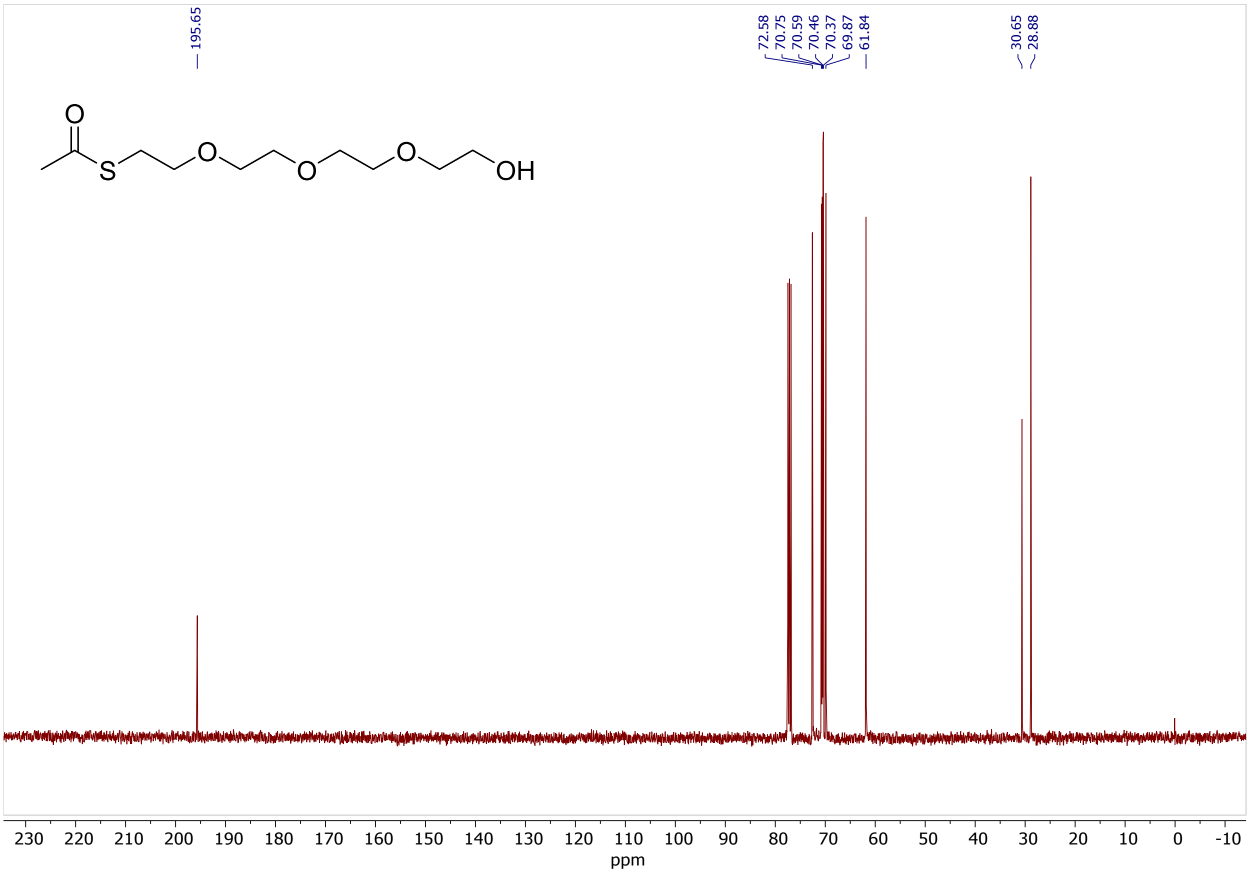

^{13}C{^{1}H}-NMR (101 MHz, $CDCl_3$) spectrum of **39**

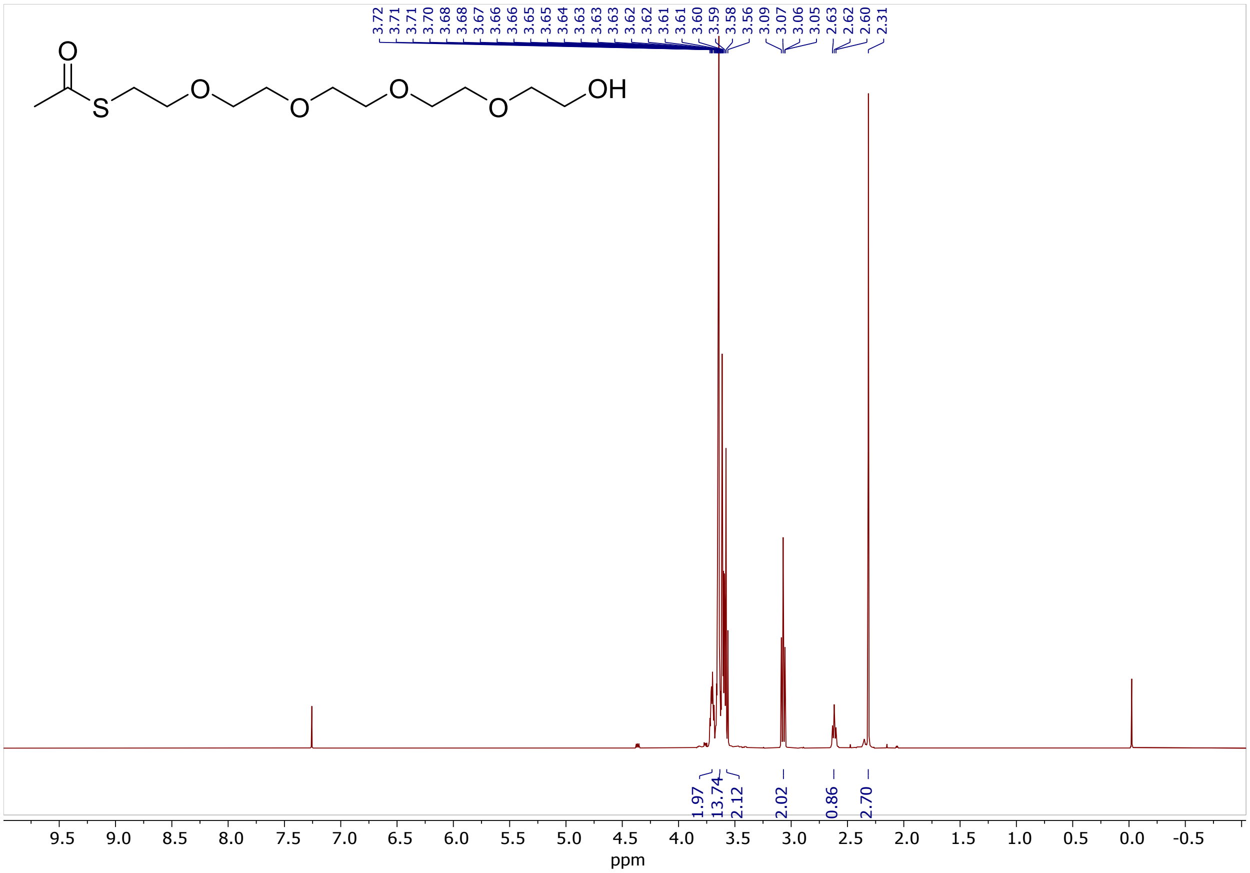

^{1}H-NMR (400 MHz, $CDCl_3$) spectrum of **40**

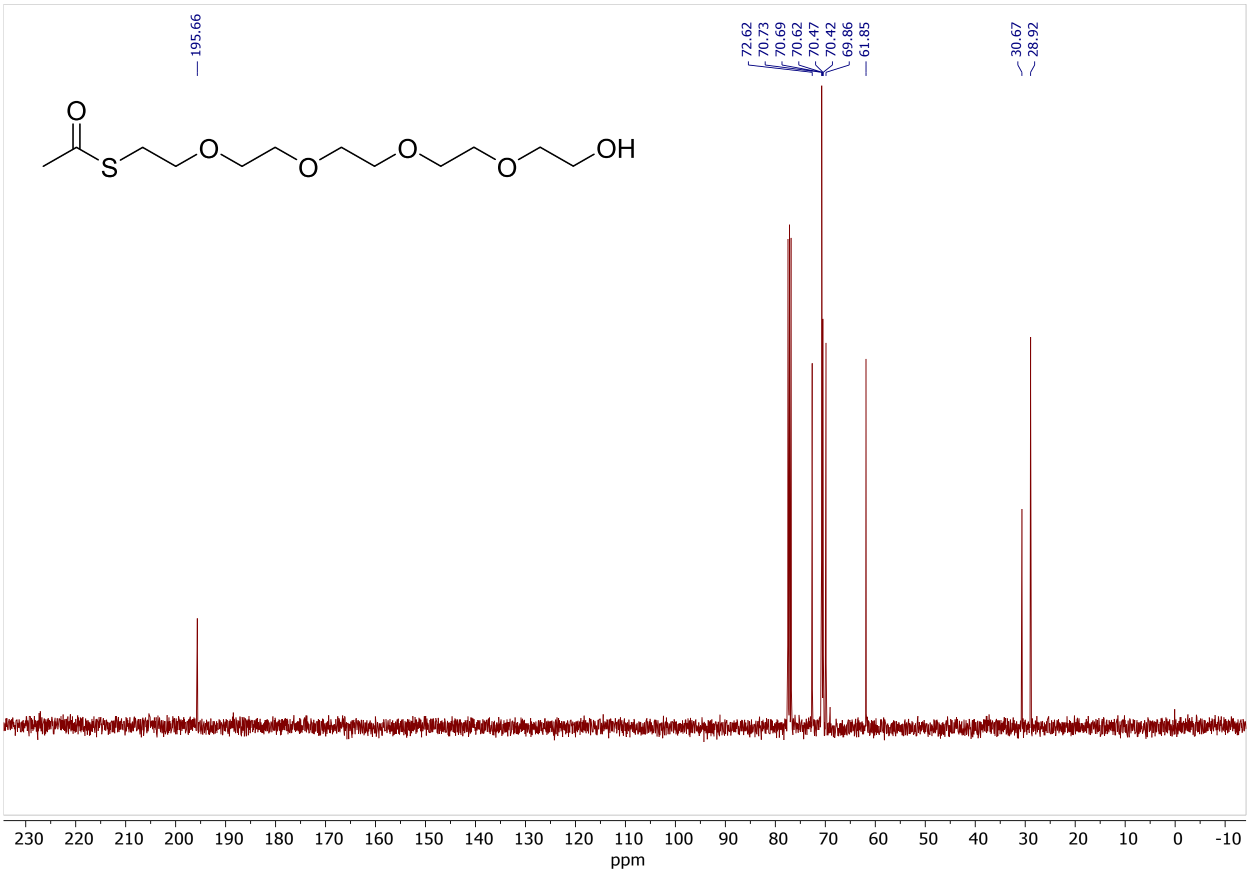

^{13}C{^{1}H}-NMR (101 MHz, $CDCl_3$) spectrum of **40**

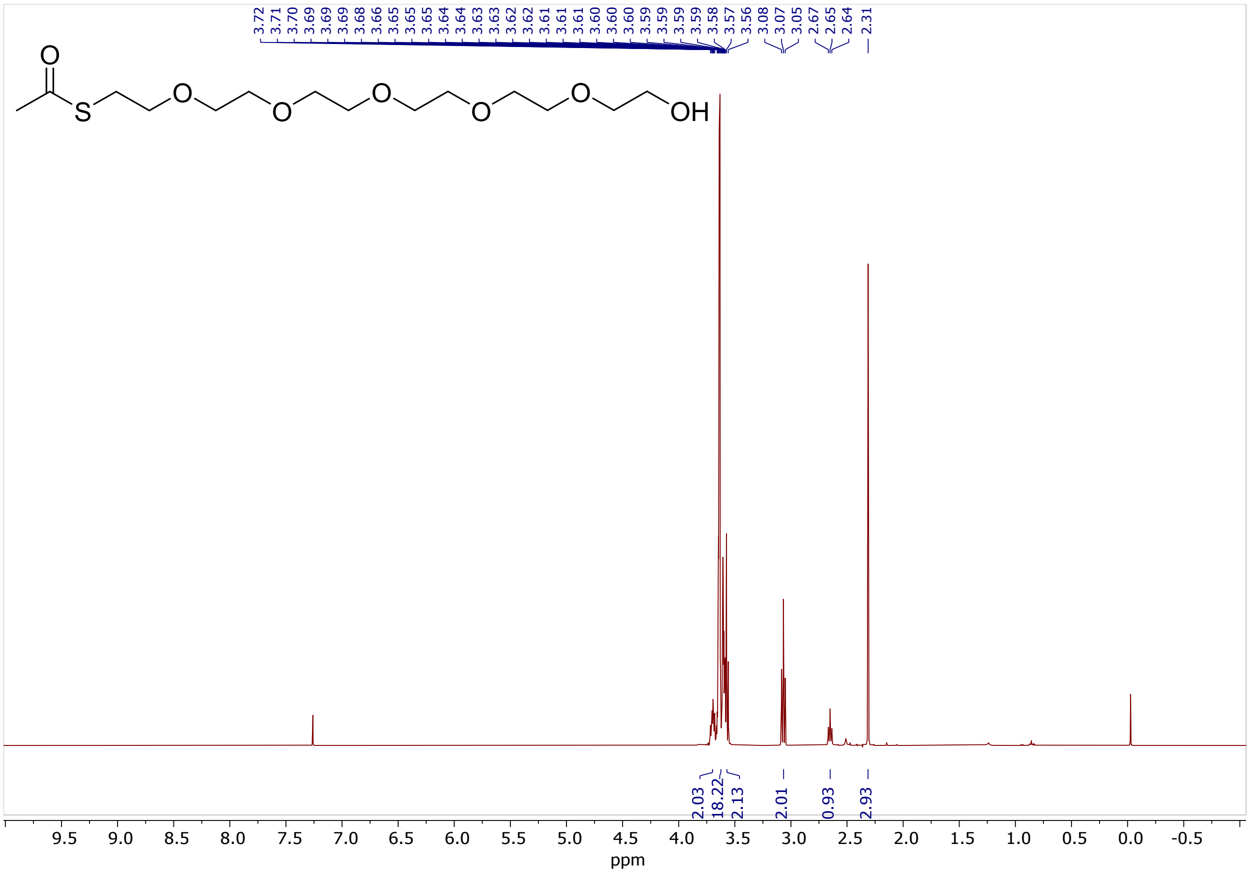

^1H-NMR (400 MHz, $CDCl_3$) spectrum of **41**

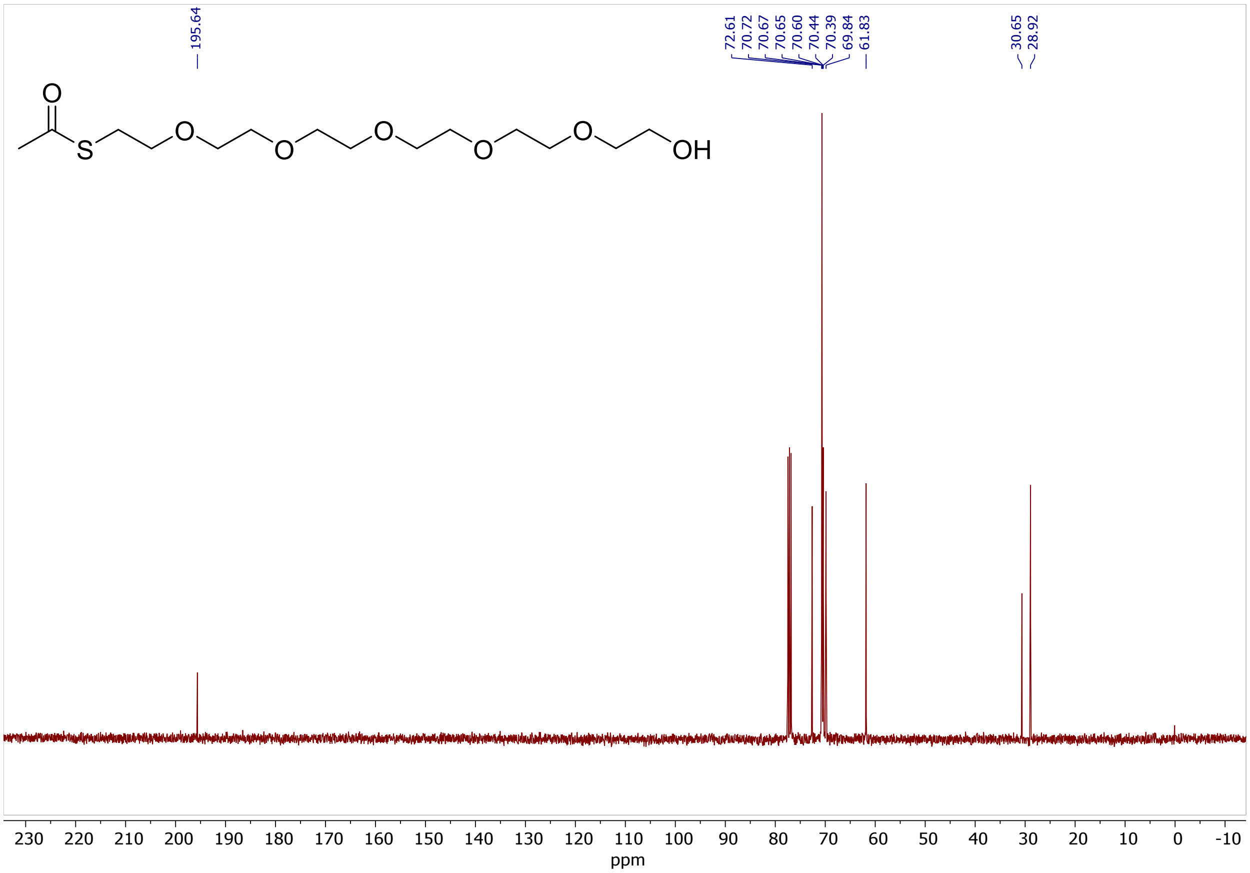

$^{13}C\{^1H\}$-NMR (101 MHz, $CDCl_3$) spectrum of **41**

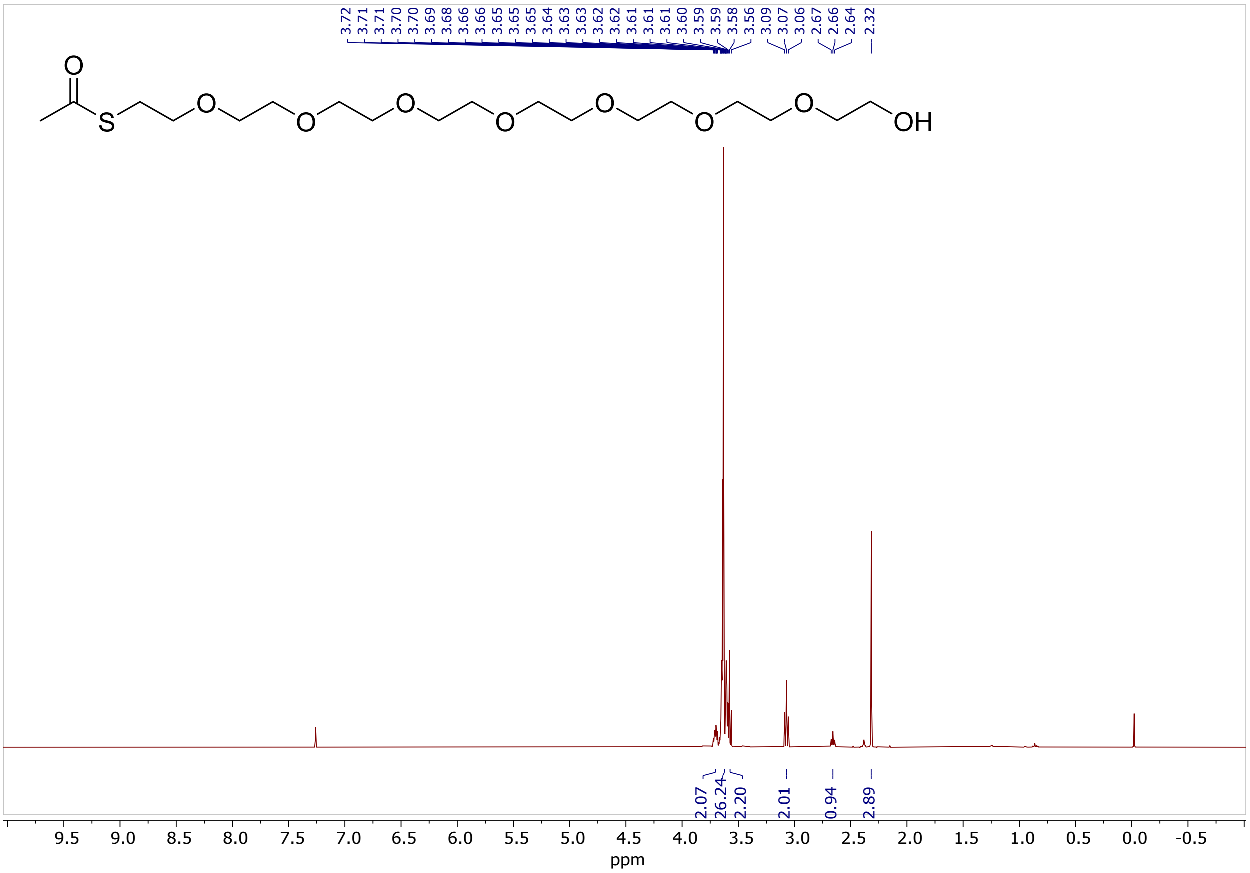

^{1}H-NMR (400 MHz, $CDCl_3$) spectrum of **42**

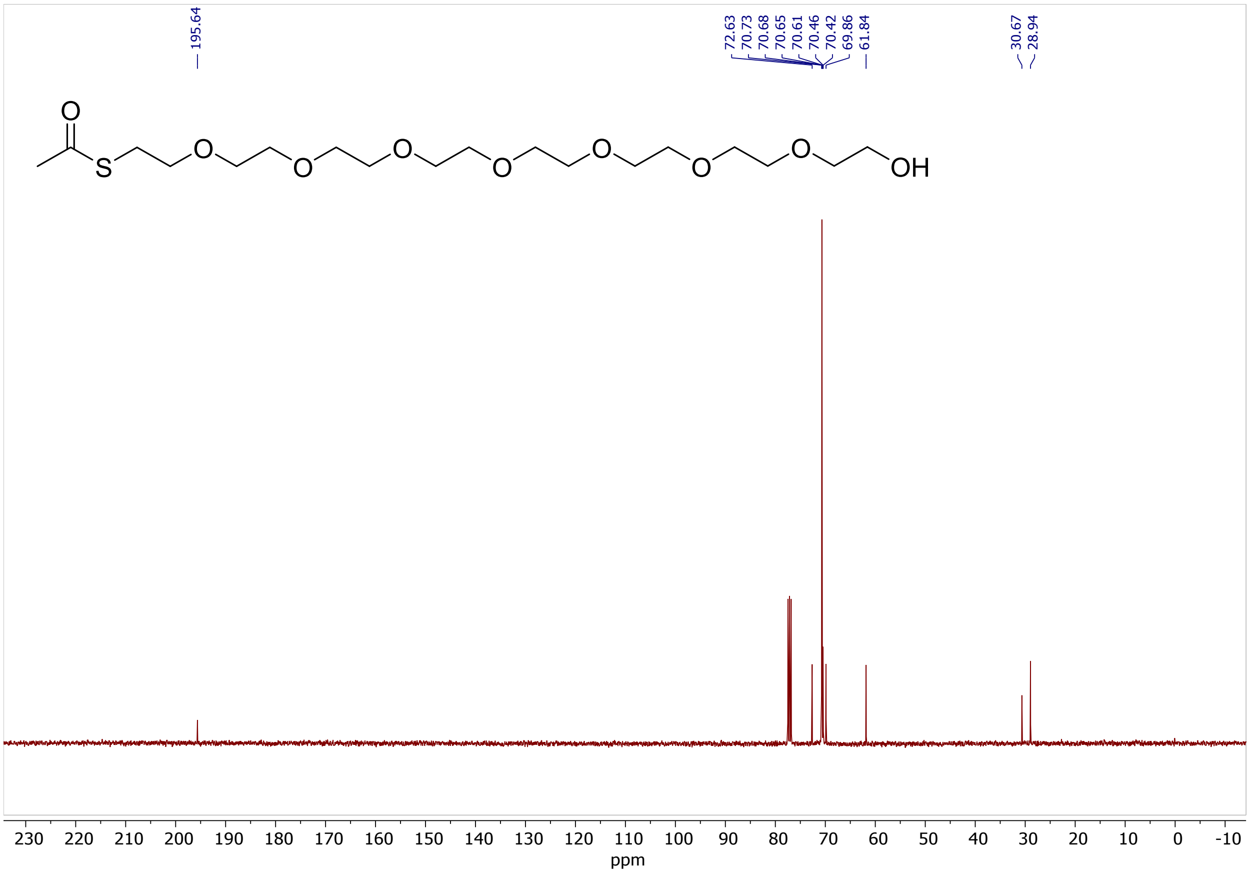

^{13}C{^{1}H}-NMR (101 MHz, $CDCl_3$) spectrum of **42**

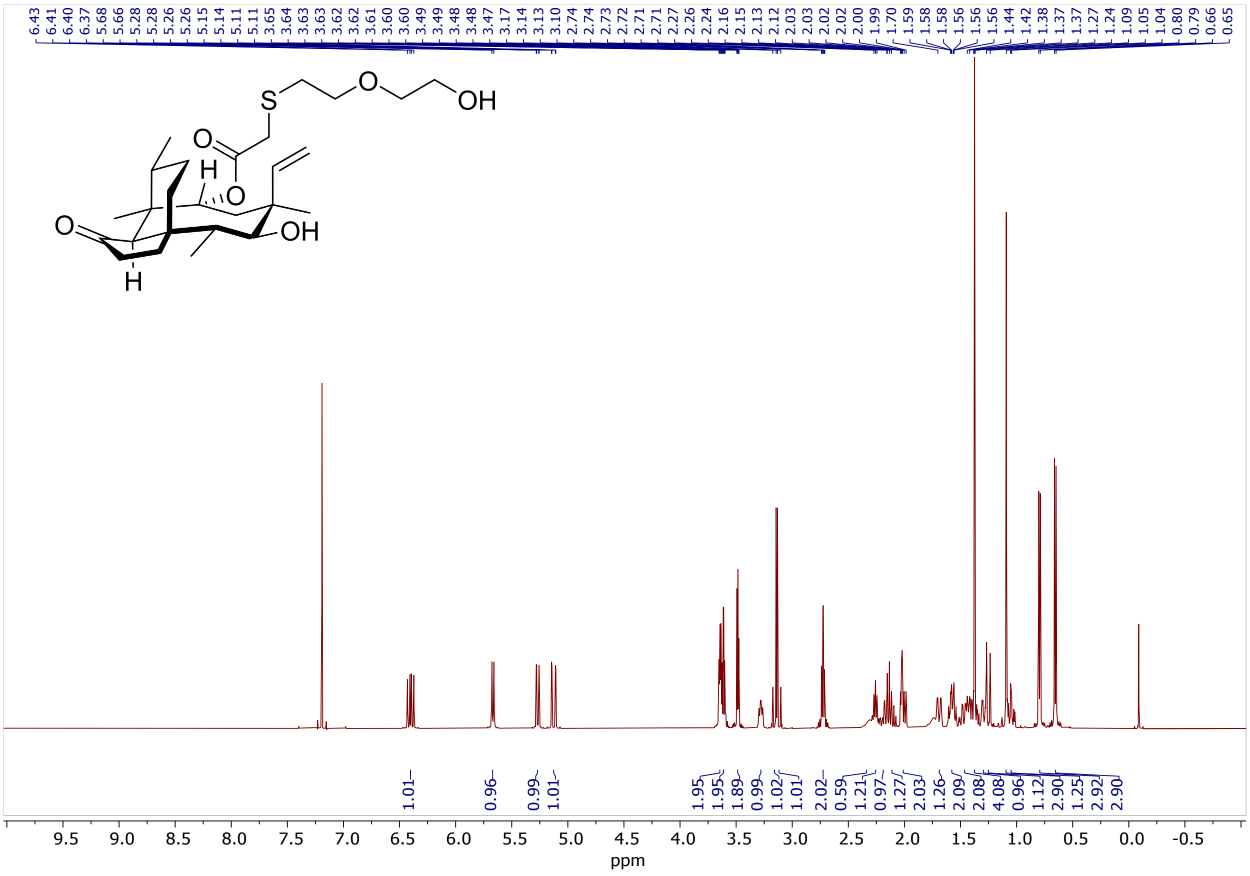

^{1}H-NMR (500 MHz, $CDCl_3$) spectrum of **43**

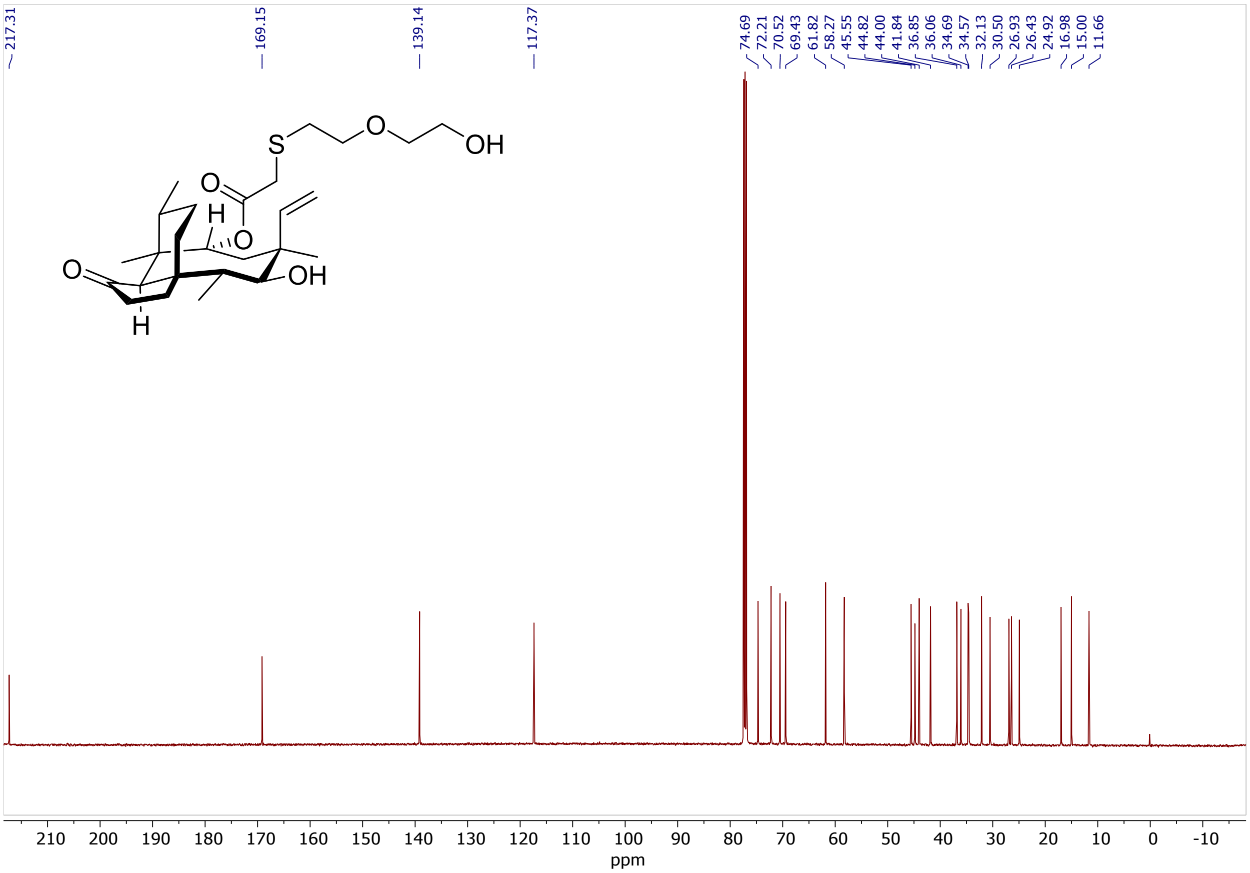

^{13}C{^{1}H}-NMR (126 MHz, $CDCl_3$) spectrum of **43**

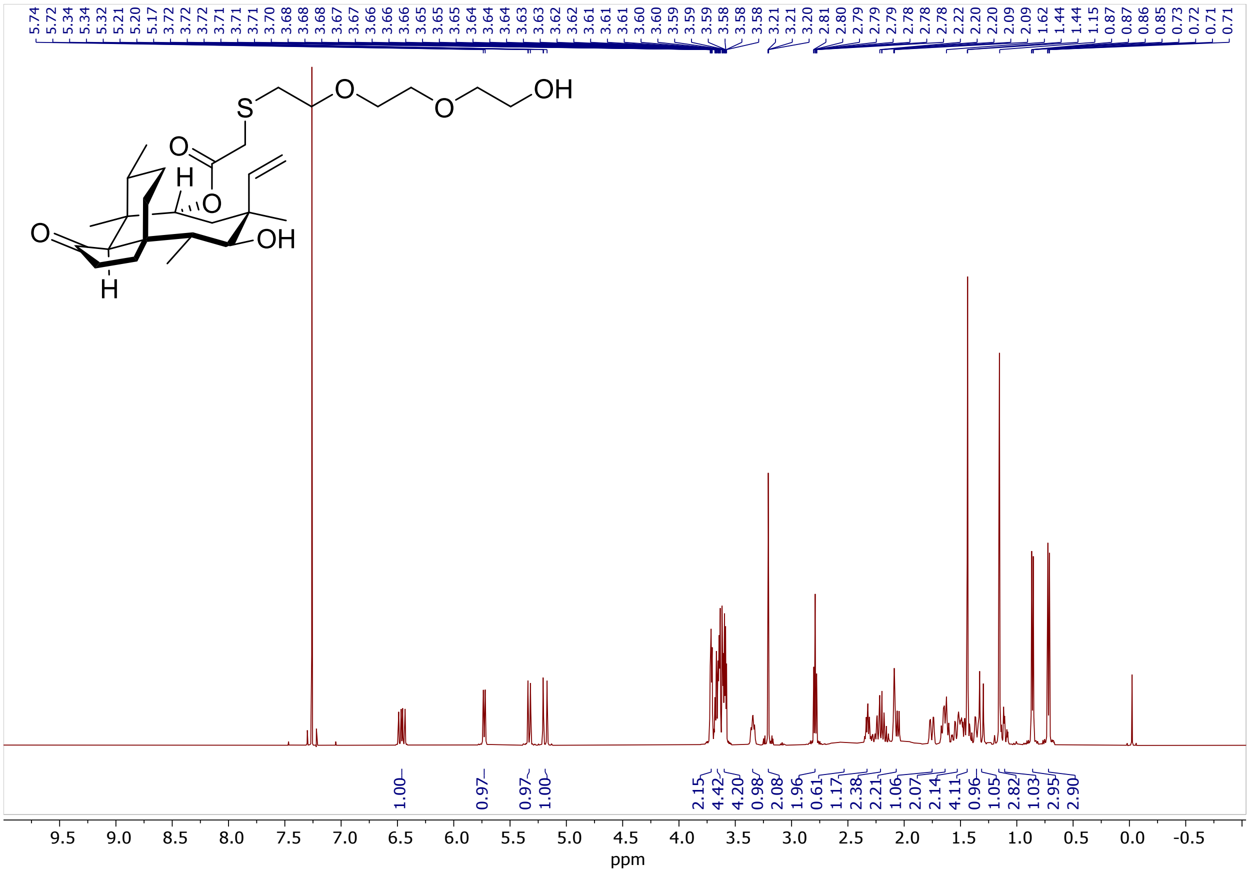

^{1}H-NMR (500 MHz, $CDCl_3$) spectrum of **44**

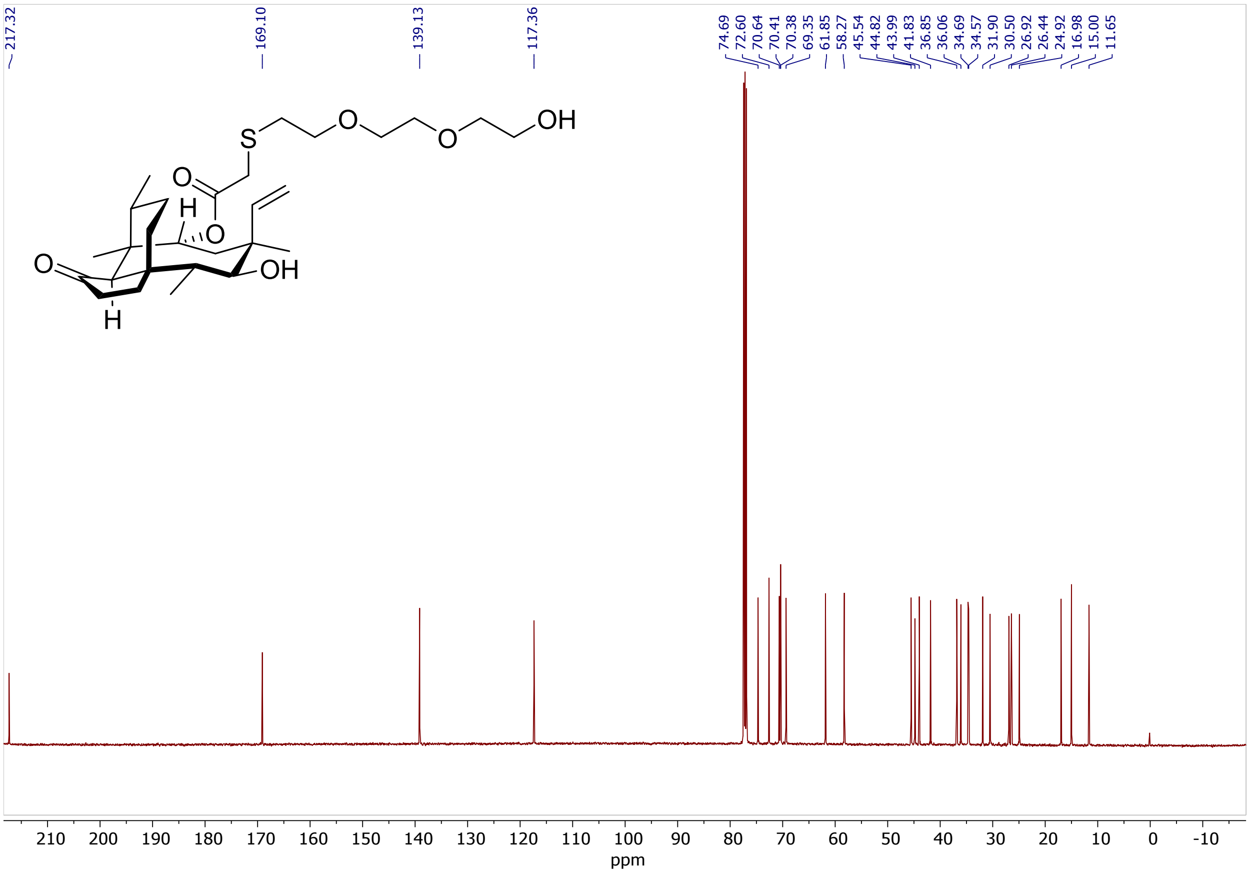

^{13}C{^{1}H}-NMR (126 MHz, $CDCl_3$) spectrum of **44**

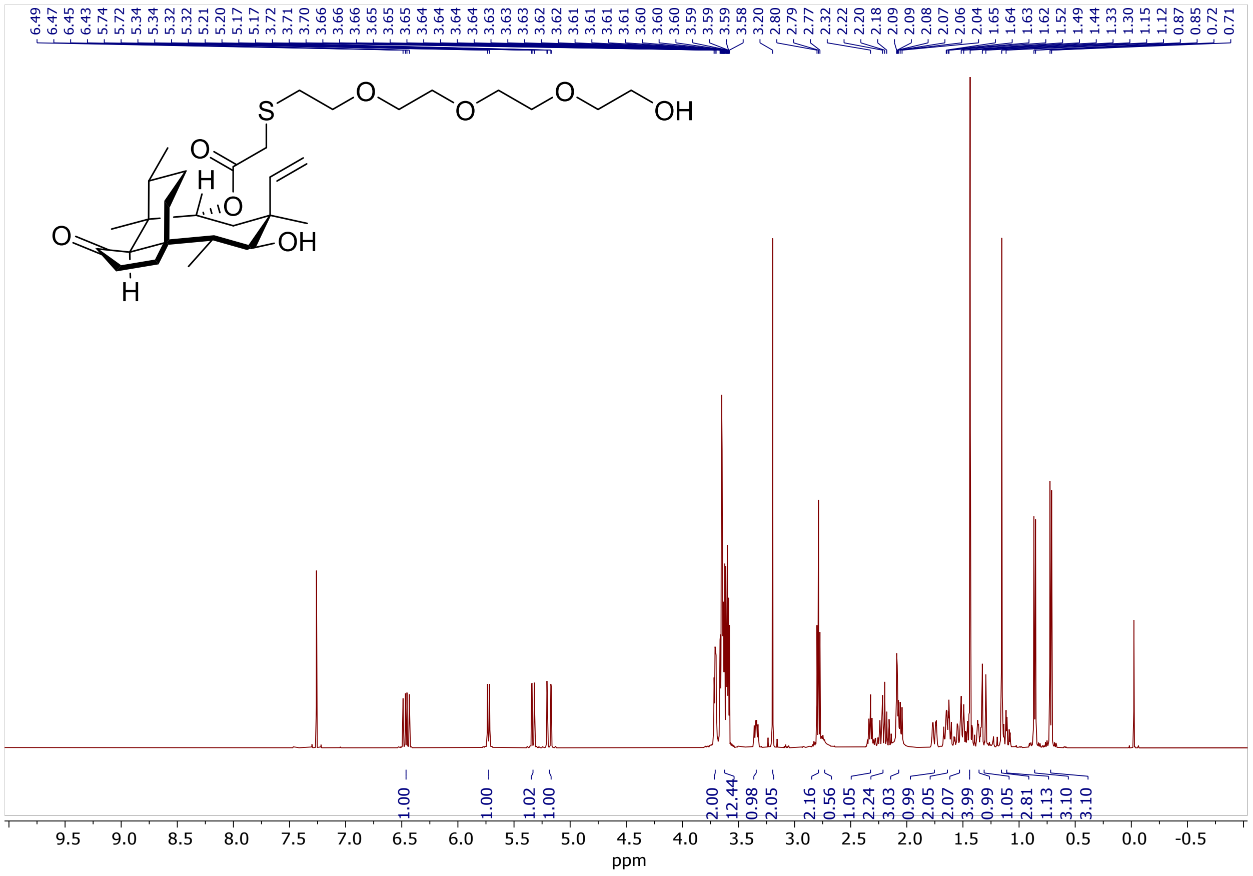

^{1}H-NMR (500 MHz, $CDCl_3$) spectrum of **45**

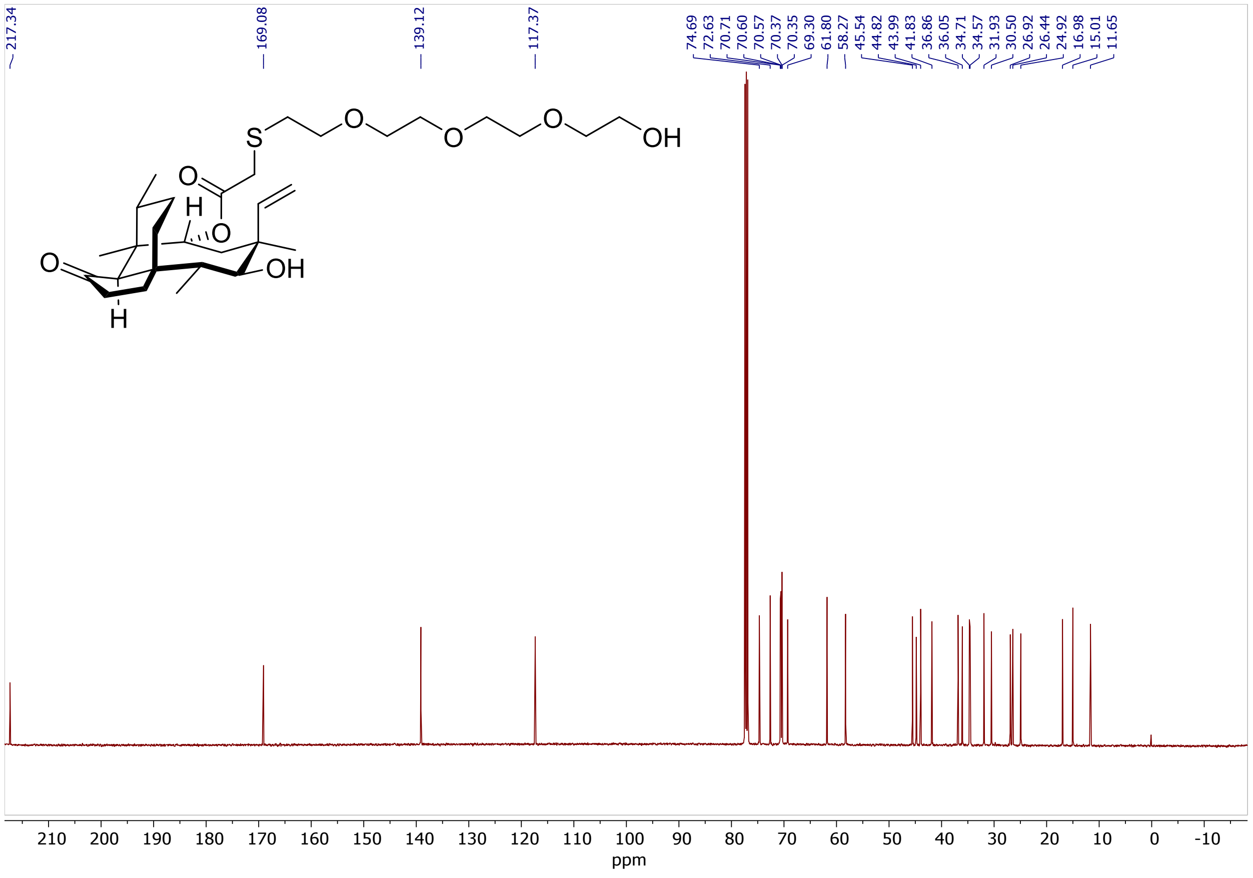

^{13}C{^{1}H}-NMR (126 MHz, $CDCl_3$) spectrum of **45**

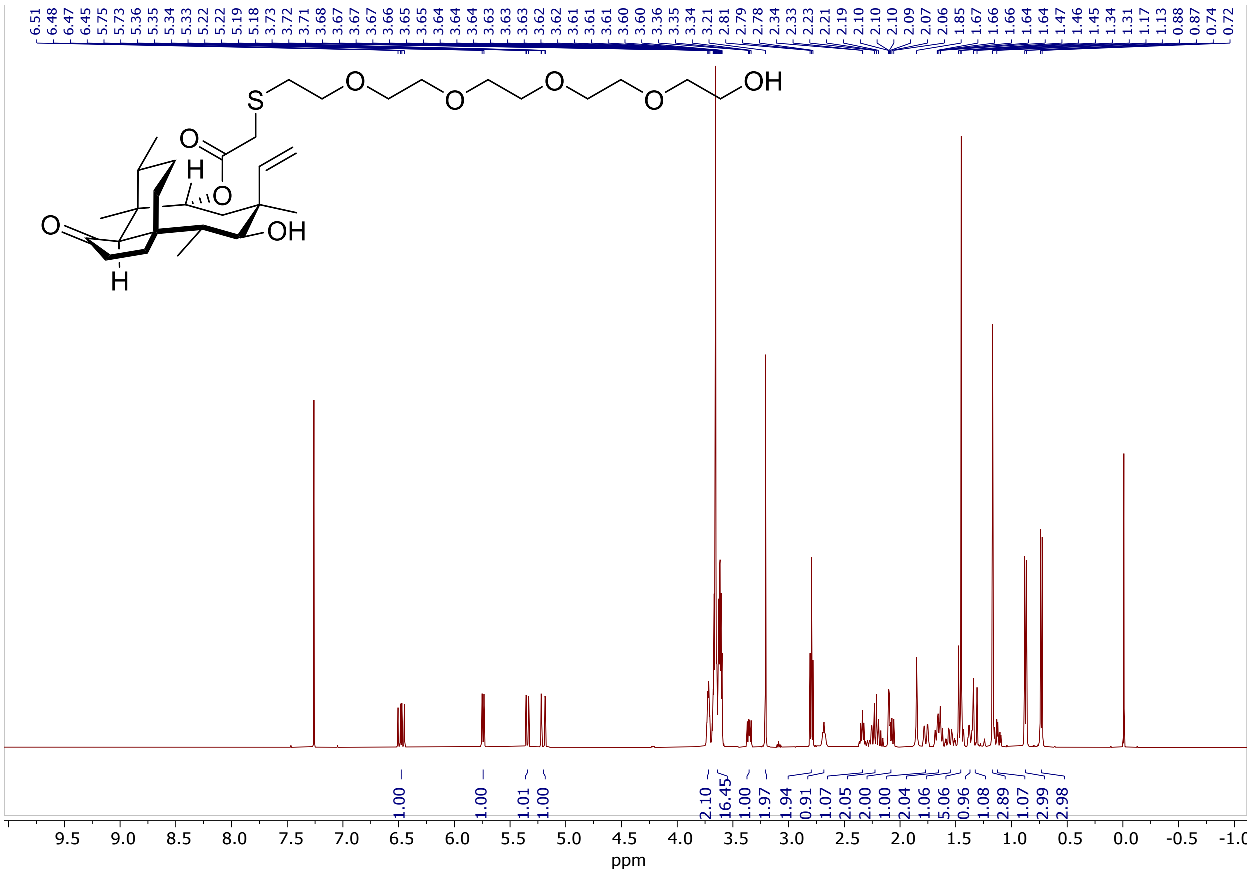

^{1}H-NMR (500 MHz, $CDCl_3$) spectrum of **46**

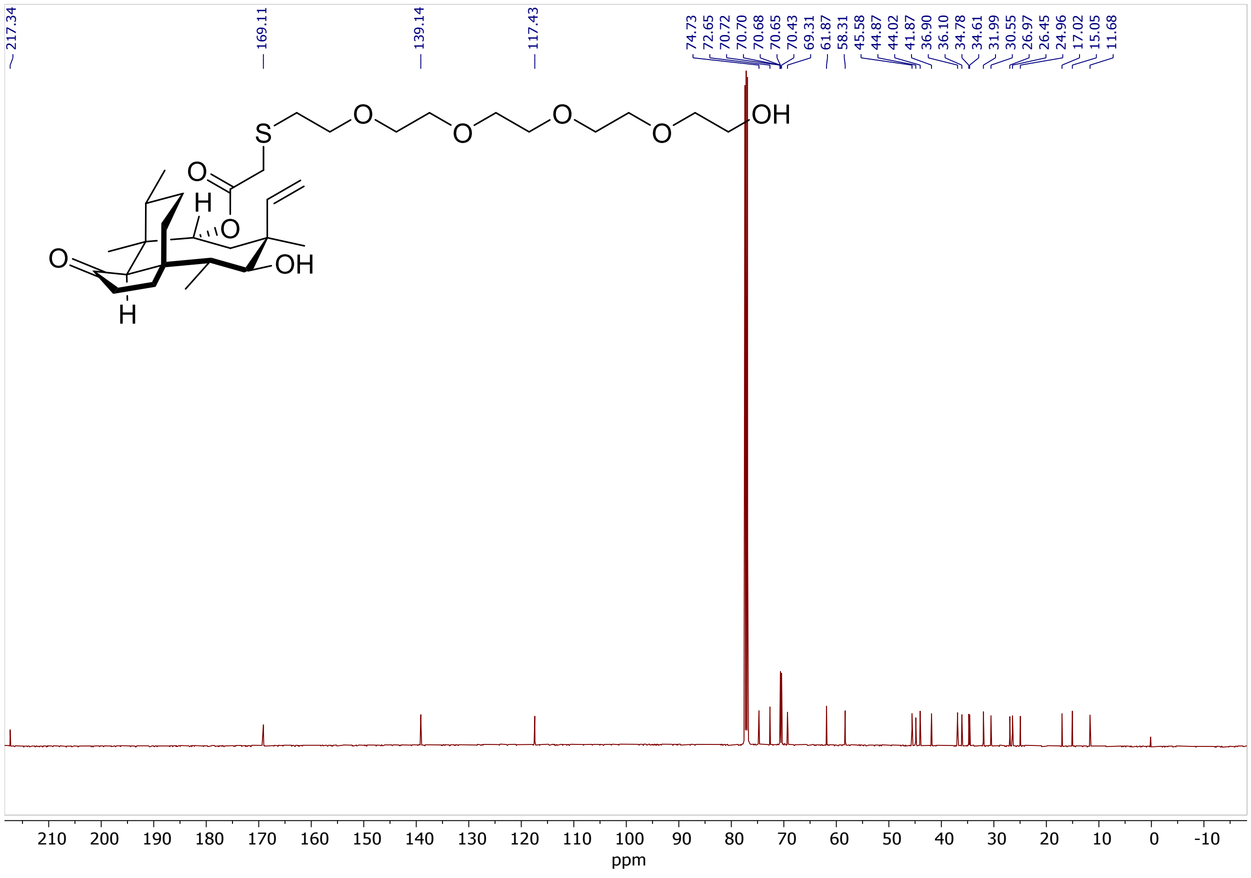

^{13}C{^{1}H}-NMR (126 MHz, $CDCl_3$) spectrum of **46**

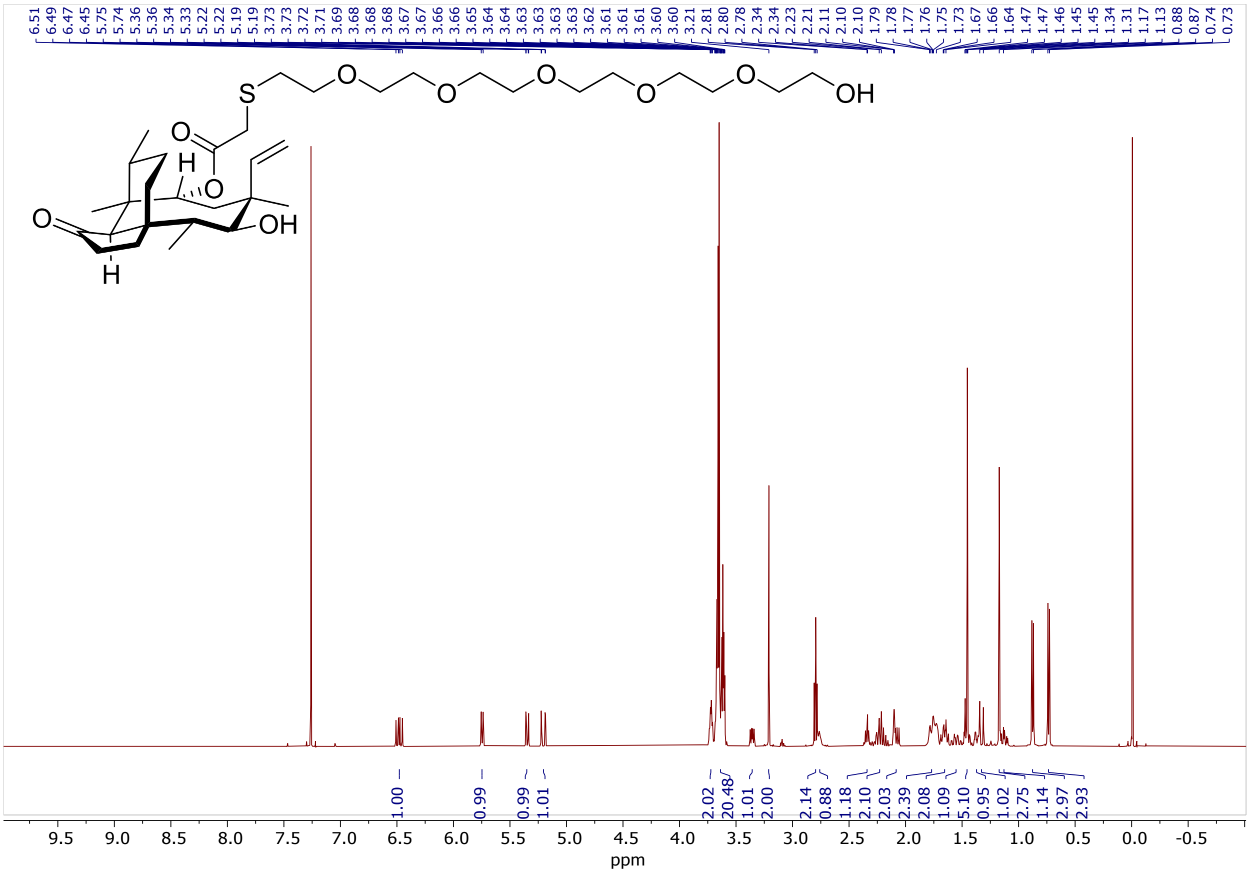

^{1}H-NMR (500 MHz, $CDCl_3$) spectrum of **47**

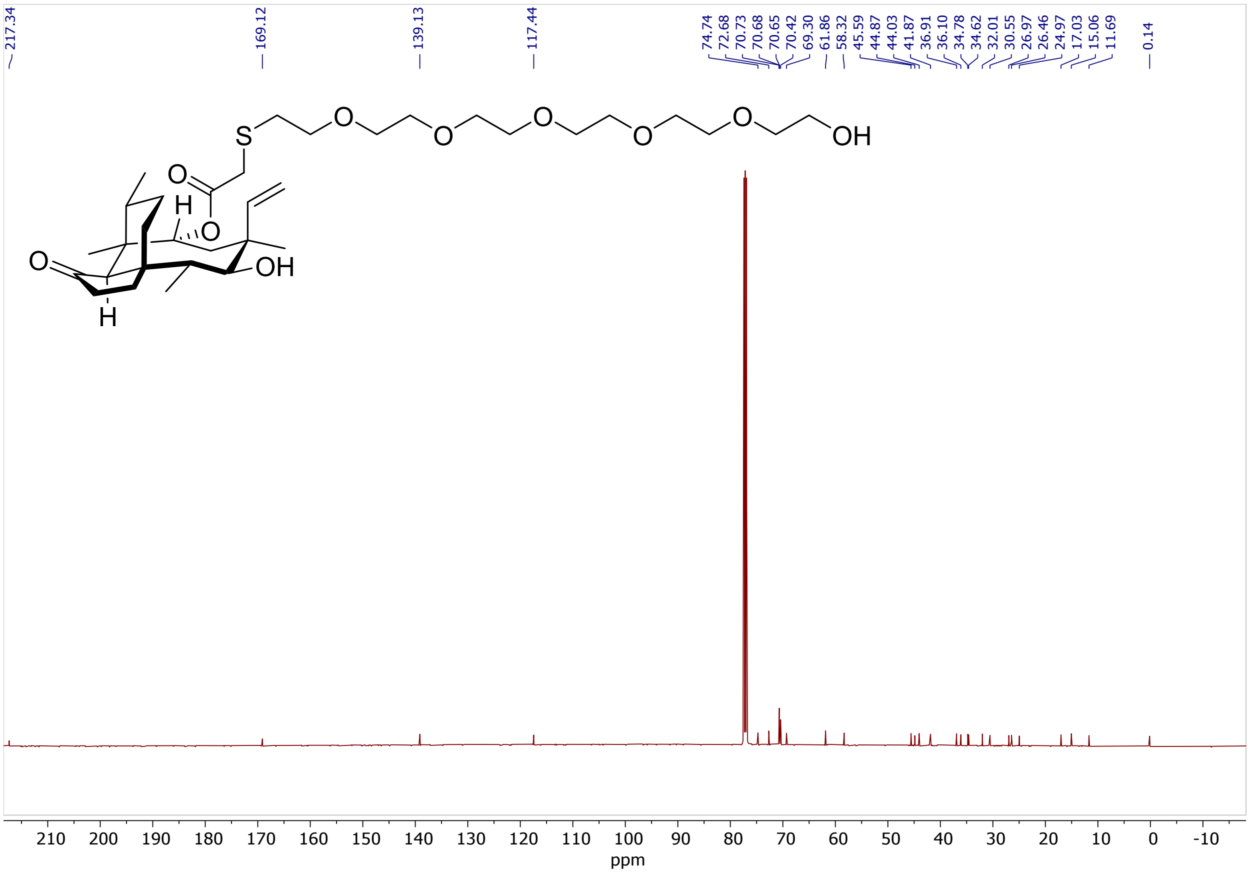

^{13}C{^{1}H}-NMR (126 MHz, $CDCl_3$) spectrum of **47**

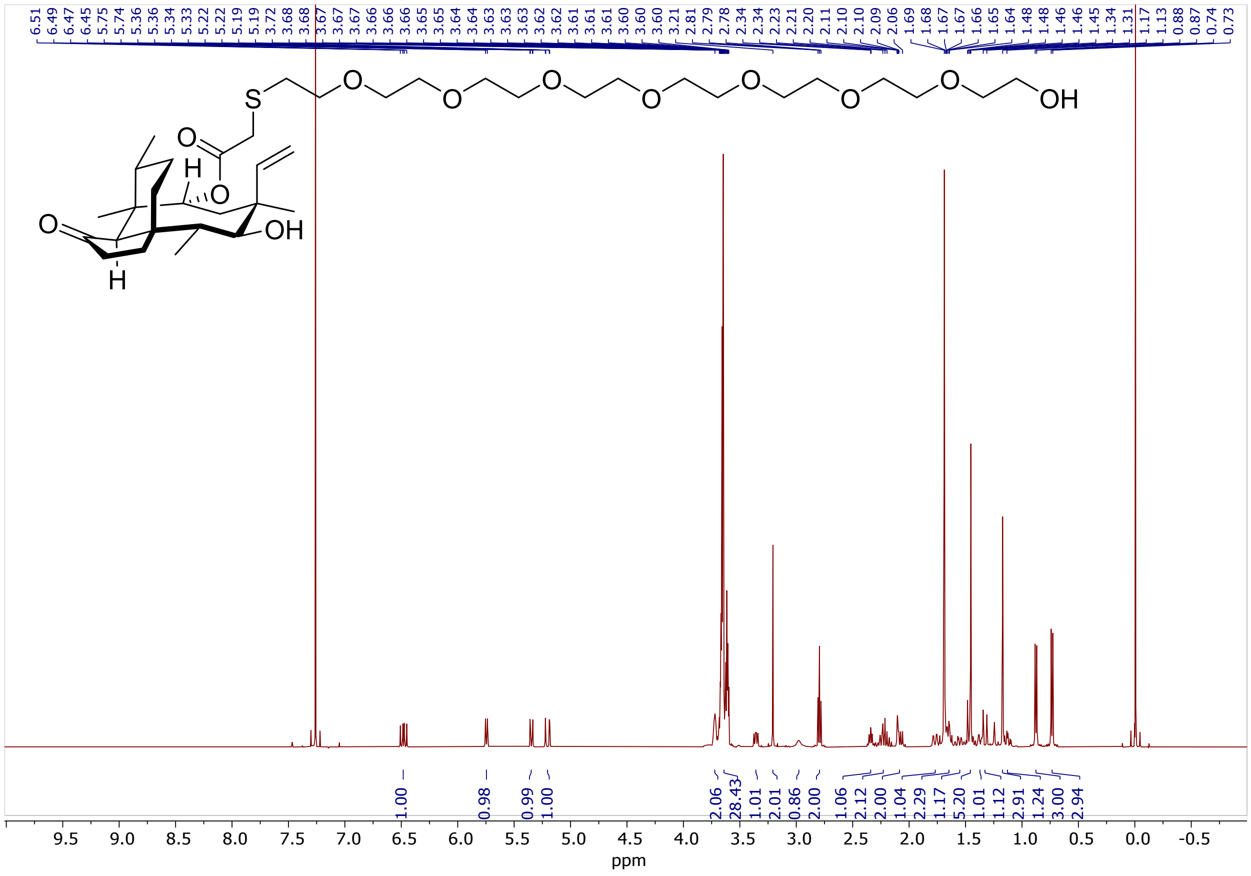

^{1}H-NMR (500 MHz, $CDCl_3$) spectrum of **48**

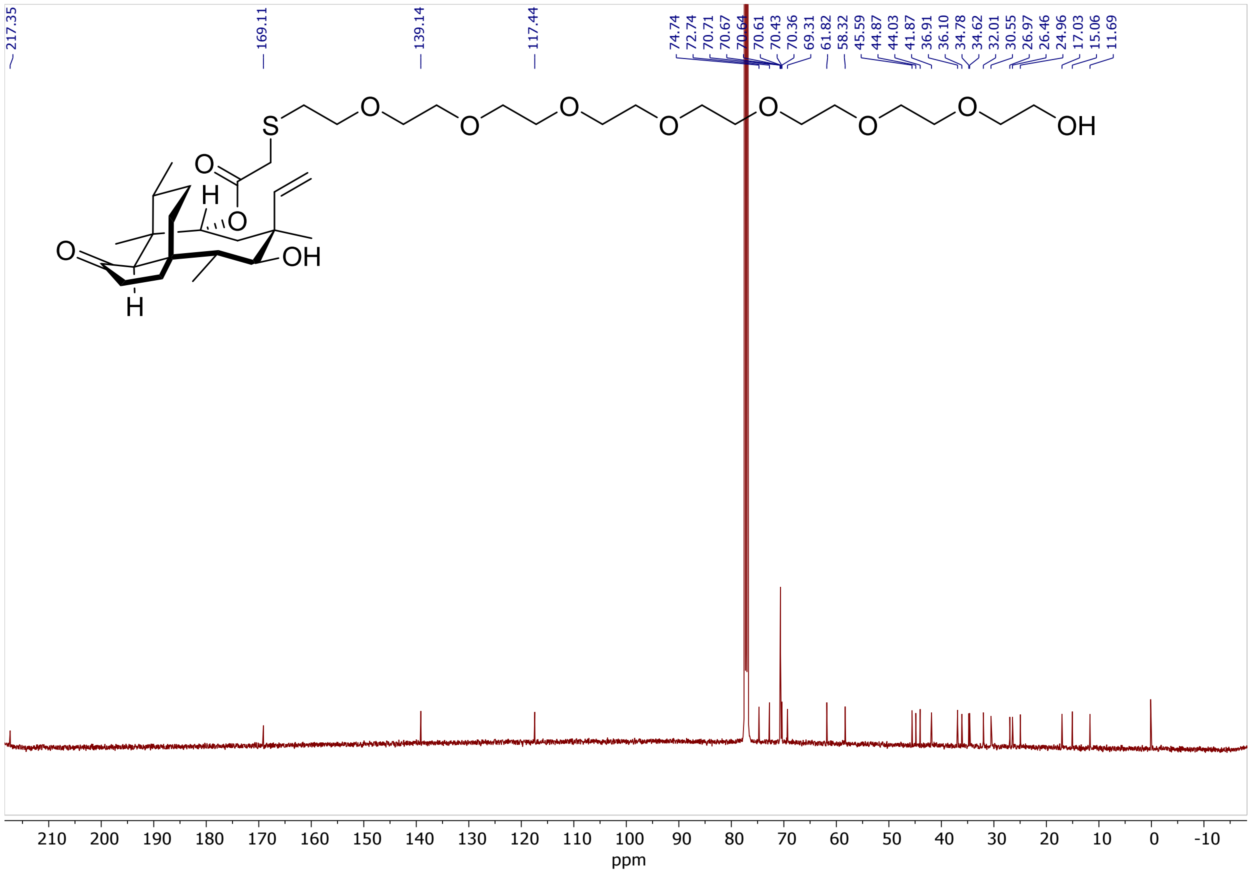

^{13}C{^{1}H}-NMR (126 MHz, $CDCl_3$) spectrum of **48**

References

1. F. Zhang, Z. Wu, P. Chen, J. Zhang, T. Wang, J. Zhou and H. Zhang, *Biorg. Med. Chem.*, 2020, **28**, 115228.
2. T. M. Geiger, M. Walz, C. Meyners, A. Kuehn, J. K. Dreizler, W. O. Sugiarto, E. V. S. Maciel, M. Zheng, F. Lermyte and F. Hausch, *Angew. Chem. Int. Ed.*, 2024, **63**, e202309706.
3. EP2842940, 2015.
4. L. M. Breiner, A. J. Briganti, J. P. McCord, M. E. Heifetz, S. Y. Philbrook, C. Slebodnick, A. M. Brown and A. N. Lowell, *Tetrahedron Chem*, 2022, **4**, 100034.
5. C. E. Katz, T. Ribelin, D. Withrow, Y. Basseri, A. K. Manukyan, A. Bermudez, C. G. Nuera, V. W. Day, D. R. Powell, J. L. Poutsma and J. Aubé, *J. Org. Chem.*, 2008, **73**, 3318-3327.
